# Neoantigen DNA vaccines are safe, feasible, and induce neoantigen-specific immune responses in triple-negative breast cancer patients

**DOI:** 10.1186/s13073-024-01388-3

**Published:** 2024-11-14

**Authors:** Xiuli Zhang, S. Peter Goedegebuure, Michael Y. Chen, Rashmi Mishra, Felicia Zhang, Yik Yeung Yu, Kartik Singhal, Lijin Li, Feng Gao, Nancy B. Myers, Tammi Vickery, Jasreet Hundal, Michael D. McLellan, Mark A. Sturmoski, Samuel W. Kim, Ina Chen, Jesse T. Davidson, Narendra V. Sankpal, Stephanie Myles, Rama Suresh, Cynthia X. Ma, Ademuyiwa Foluso, Andrea Wang-Gillam, Sherri Davies, Ian S. Hagemann, Elaine R. Mardis, Obi Griffith, Malachi Griffith, Christopher A. Miller, Ted H. Hansen, Timothy P. Fleming, Robert D. Schreiber, William E. Gillanders

**Affiliations:** 1 Department of Surgery, Washington University School of Medicine, Saint Louis, MO USA; 2 Department of Medicine, Washington University School of Medicine, Saint Louis, MO USA; 3 Bursky Center for Human Immunology & Immunotherapy Programs, Washington University School of Medicine, Saint Louis, MO USA; 4 McDonnell Genome Institute, Washington University School of Medicine, Saint Louis, MO USA; 5 Department of Pathology and Immunology, Washington University School of Medicine, Saint Louis, MO USA; 6 The Alvin J. Siteman Cancer Center at Barnes-Jewish Hospital and Washington University School of Medicine, Saint Louis, MO USA; 7 Current Affiliation: Department of Pediatrics, Nationwide Children’s Hospital and The Ohio State University College of Medicine, Columbus, OH USA

**Keywords:** Phase I, Clinical trial, TNBC, DNA Vaccine, Neoantigen, Immune response

## Abstract

**Background:**

Neoantigen vaccines can induce or enhance highly specific antitumor immune responses with minimal risk of autoimmunity. We have developed a neoantigen DNA vaccine platform capable of efficiently presenting both HLA class I and II epitopes and performed a phase 1 clinical trial in triple-negative breast cancer patients with persistent disease on surgical pathology following neoadjuvant chemotherapy, a patient population at high risk of disease recurrence.

**Methods:**

Expressed somatic mutations were identified by tumor/normal exome sequencing and tumor RNA sequencing. The pVACtools software suite of neoantigen prediction algorithms was used to identify and prioritize cancer neoantigens and facilitate vaccine design for manufacture in an academic GMP facility. Neoantigen DNA vaccines were administered via electroporation in the adjuvant setting (i.e., following surgical removal of the primary tumor and completion of standard of care therapy). Vaccines were monitored for safety and immune responses via ELISpot, intracellular cytokine production via flow cytometry, and TCR sequencing.

**Results:**

Eighteen subjects received three doses of a neoantigen DNA vaccine encoding on average 11 neoantigens per patient (range 4–20). The vaccinations were well tolerated with relatively few adverse events. Neoantigen-specific T cell responses were induced in 14/18 patients as measured by ELISpot and flow cytometry. At a median follow-up of 36 months, recurrence-free survival was 87.5% (95% CI: 72.7–100%) in the cohort of vaccinated patients.

**Conclusion:**

Our study demonstrates neoantigen DNA vaccines are safe, feasible, and capable of inducing neoantigen-specific immune responses.

**Clinical trial registration number:**

NCT02348320.

**Supplementary Information:**

The online version contains supplementary material available at 10.1186/s13073-024-01388-3.

## Background

Cancer neoantigens are mutant proteins/amino acid sequences expressed in tumors that can be recognized by the immune system. Cancer sequencing and related bioinformatics technologies have revolutionized our ability to identify cancer neoantigens. We performed one of the first preclinical studies to apply an immunogenomics approach to neoantigen identification [1]. This study demonstrated that cancer neoantigens are important targets of cancer immunoediting, and established the initial proof of concept that cancer exome sequencing and epitope prediction algorithms can be used to identify cancer neoantigens. In subsequent preclinical studies, we demonstrated that neoantigen vaccines can induce neoantigen-specific CD8 and CD4 T cell responses and antitumor immunity [2–5]. Other investigators have used similar strategies in the B16F10 (melanoma), TRAMP-C1 (prostate cancer), CT26, and MC-38 (colon cancer) mouse tumor models [6–8]. Because of the high mutational load and documented immunogenicity of human melanoma, initial clinical studies were carried out with melanoma patients using different vaccine platforms [9–11]. The first report of a neoantigen vaccine strategy in humans demonstrated that neoantigen dendritic cell vaccines are capable of generating neoantigen-specific T cell responses in human melanoma patients [9]. Two papers co-published in *Nature* by Ott et al. [11] and Sahin et al. [10] confirmed the potential of neoantigen vaccines in treating melanoma patients using neoantigen synthetic long peptide (SLP) and RNA neoantigen vaccine approaches, respectively. Additional studies have evaluated neoantigen vaccines in glioblastoma [12, 13], pancreatic cancer [14], and other cancer types as recently reviewed in [15]. The first clinical trials confirmed neoantigen vaccines are safe and capable of inducing neoantigen-specific and antitumor immunity [16].

Triple-negative breast cancer (TNBC) lacks expression of estrogen receptor, progesterone receptor, and HER2 gene amplification. TNBC is associated with an aggressive clinical course, and there are no targeted therapies available [17]. There is a strong rationale to target cancer neoantigens in TNBC. First, TNBC is a mutationally complex breast cancer subtype [18–20]. The relative abundance of somatic mutations in TNBC suggests that neoantigens that can be targeted by neoantigen vaccine therapy are more likely to be present [20]. Second, tumor-infiltrating lymphocytes (TILs) are more common in TNBC than in other breast cancer subtypes, and TILs are associated with improved outcomes in TNBC following adjuvant, or neoadjuvant chemotherapy [21–23]. The association between TILs and improved outcomes in TNBC highlights the importance of the adaptive immune system in the response to therapy. Third, several recent studies of chemotherapy combined with immune checkpoint inhibition in TNBC suggest that a percentage of patients with TNBC will benefit from combination immunotherapy with durable responses noted [24–27]. While the studies collectively suggest TNBC is an attractive candidate for neoantigen vaccine therapy, no neoantigen DNA vaccine studies have been reported in breast cancer to date.

Our efforts towards improving clinical outcomes in TNBC have focused on a neoantigen DNA vaccine strategy. The observation that direct administration of recombinant DNA can generate potent immune responses established the field of DNA vaccines in the early 1990s [28–33]. Since that time, DNA vaccines have remained an area of intense research interest, and vaccines targeting infectious disease agents and cancers have progressed into clinical trials. The DNA vaccine platform affords flexibility by allowing targeting of multiple neoantigens using a single polyepitope DNA vaccine. We have designed such a neoantigen DNA vaccine platform that also integrates a mutant ubiquitin molecule in order to promote epitope generation and display [5]. In the present study, we used a TriGrid electroporation device to administer the neoantigen DNA vaccines. Electroporation dramatically increases DNA uptake by muscle cells, antigen expression, and immunogenicity [34–37]. Of particular note, electroporation has now been used successfully in non-human primates and in human clinical trials, with responses at levels not previously observed with other DNA vaccine approaches and similar to or superior to those induced by live vectors [38–48].

We have completed a phase 1 clinical trial of a neoantigen DNA vaccine strategy in patients with persistent TNBC following neoadjuvant chemotherapy (NCT02348320). We report here on the safety, immunogenicity, and potential clinical impact of the neoantigen DNA vaccine strategy.

## Methods

### Clinical trial

**Fig. 1:**
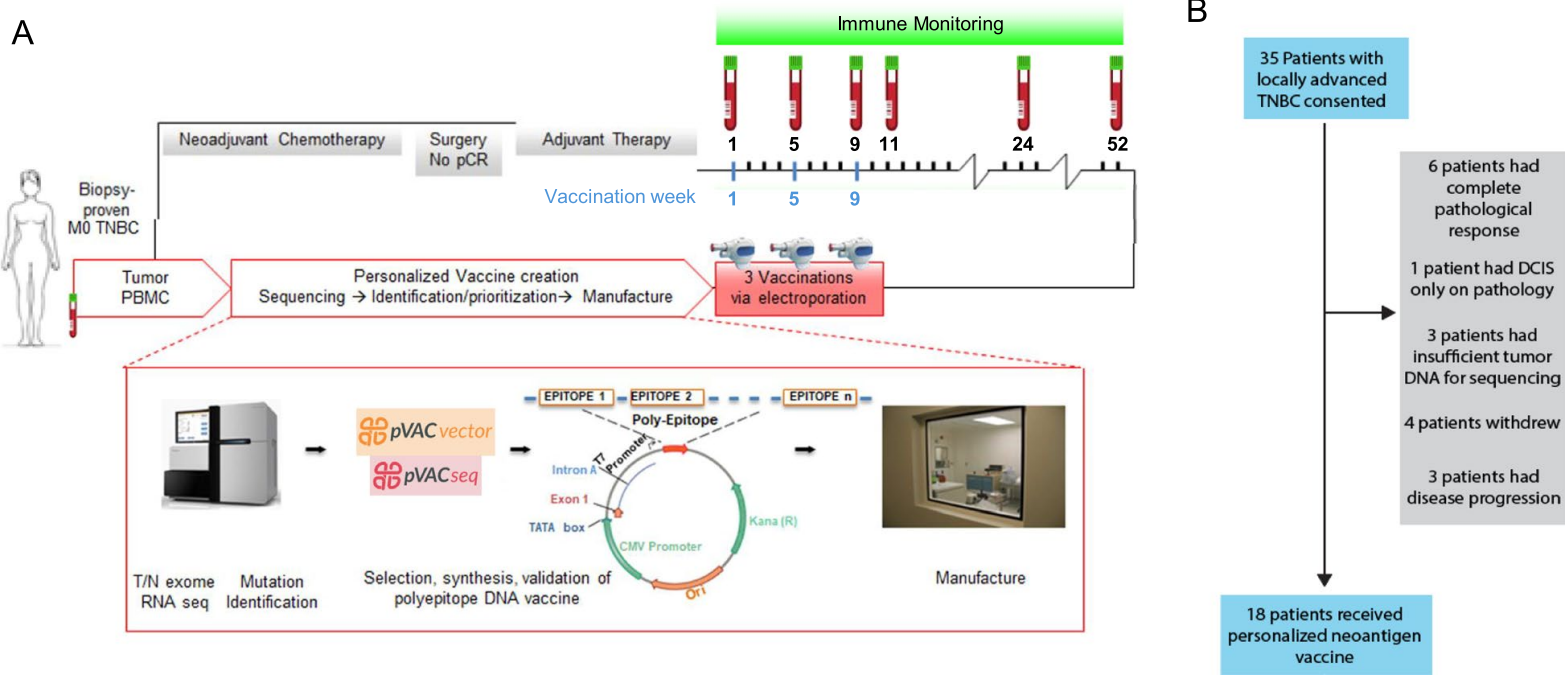
Design, manufacture, and administration of neoantigen DNA vaccines for TNBC patients. **A** Somatic mutations were identified by whole exome sequencing of tumor and germline DNA. Mutation expression was confirmed by tumor RNA-seq with cDNA capture. Candidate neoantigens were prioritized for inclusion in the vaccines on the basis of HLA binding predictions by pVAC-seq (Methods). Neoantigen DNA vaccines were administered intramuscularly using a TriGrid electroporation device. Peripheral blood was drawn prior at each vaccination timepoint and at selected timepoints after all vaccinations as indicated in **A**. **B** 35 patients with locally advanced TNBC were consented. Patients were excluded due to complete pathological response after neoadjuvant chemotherapy (NAC), insufficient tumor, patient withdrawal, and disease recurrence. 18 patients received personalized neoantigen DNA vaccines

The clinical protocol was reviewed and approved by the Institutional Review Board at Washington University School of Medicine. Patients were consented over a period of 35 months from June 2015 to February 2018. Patients with persistent TNBC following neoadjuvant chemotherapy were eligible for participation. Patients with evidence of metastatic breast cancer or autoimmune disorders were excluded. Subjects enrolled into the protocol provided germline (peripheral blood) and tumor DNA samples, and consent for tumor/normal exome sequencing and data sharing in a controlled access database (dbGaP). Tumor biopsies (12 patients) or tissue obtained at the time of surgery (6 patients), and matched PBMCs were subjected to nucleic acid isolation followed by tumor/normal exome sequencing to identify somatic mutations resulting in altered protein/amino acid sequences (Additional file 1: Fig. S1A, Fig. 1A). After initial consent, some subjects were determined to be ineligible or were excluded (insufficient tumor material for sequencing, patient withdrawal, and/or disease recurrence, Fig. 1). Ultimately 18 subjects out of 35 consented patients received a neoantigen DNA vaccine.

All subjects were vaccinated with 4 mg of neoantigen DNA vaccine at day 1, day 29 ± 7, and day 57 ± 7. Each neoantigen DNA vaccine was administered intramuscularly using a TriGrid™ electroporation device (ICHOR Medical Systems, San Diego, CA). Peripheral blood was drawn prior to each vaccination and after vaccination. Peripheral blood mononuclear cells (PBMC) were isolated through density centrifugation using Ficoll-Paque PLUS (GE Healthcare Bio-Science AB, Sweden) and cryopreserved as cell suspensions. Each subject was monitored through follow-up visits at weeks 11, 24, and 52, with additional follow-up visits or telephone contact annually thereafter. The primary objective of the clinical trial was to evaluate the safety of the neoantigen DNA vaccine strategy. Safety was closely monitored after vaccination with eight or more clinical and laboratory assessments in the first 6 months of the trial. Toxicity was graded according to the National Cancer Institute Common Terminology Criteria for Adverse Events (CTCAE) version 4.0. The secondary objective was to evaluate the immunogenicity of the neoantigen DNA vaccine strategy as measured by ELISpot analysis and multi-parametric flow cytometry, both surrogates for CD8 T cell function.

### Tissue procurement and nucleic acid isolation

Archival tumor samples were obtained. H&E-stained sections were scored by a pathologist for tumor content and necrosis. Tissue blocks with over 60% tumor purity were selected, if available. DNA from PBMC was extracted using the QIAamp DNA Mini Kit (Qiagen Sciences, Maryland), and DNA and RNA were extracted from tumor tissues using the AllPrep DNA/RNA FFPE Kit (Qiagen Sciences). DNA and RNA quality were determined using an Agilent Bioanalyzer (Agilent, Santa Clara, CA), and quantitated using a Qubit Fluorometer (Life Technologies, Carlsbad, CA).

### Exome sequencing

For each subject, tumor/normal DNA samples were processed for whole exome sequencing. Libraries were prepared using the KAPA Biosystems NGS kit (Roche Sequencing and Life Science, Indianapolis, IN) and captured using the IDT xGen Exome v1 panel (Integrated DNA Technologies, Inc., Coralville, IA) using the manufacturer-recommended procedure. Sequence data [77] were generated as either 2 × 101 bp or 2 × 126 bp read pairs on an Illumina HiSeq instrument. DNA library preparation and sequencing were performed in a CLIA-compliant space. Sequence alignment and somatic variant calling were performed as described previously [49], using an ensemble of callers and stringent filtering, followed by variant effect prediction using VEP [50].

### cDNA-capture sequencing

RNA samples were prepared using the Illumina TruSeq Stranded kit to produce cDNA, followed by cDNA capture with the IDT xGen Exome v1 panel. Both steps followed manufacturer-recommended protocols with the exception of skipping the ribodepletion step on samples with low RNA yields (BRC45 and BRC10). Sequencing was performed on an Illumina HiSeq instrument, producing either 2 × 101 bp or 2 × 126 bp paired-end reads. Reads were trimmed and aligned with HISAT version 2.0.5 [51], and expression was quantified using kallisto version 0.43.1 [52] and transcripts from Ensembl release 95 [53].

### HLA typing

All patients’ HLA class I haplotypes were determined by PCR-SSOP (ProImmune, Sarasota, FL) using PBMC.

### Neoantigen identification

We used the pVACtools [54] pipeline to identify and shortlist potential high-affinity neoantigens resulting from somatic missense mutations detected by exome sequencing. Briefly, amino acid substitutions corresponding to each of the coding missense mutations were translated into a 25-mer amino acid FASTA sequence, with up to 12 amino acids flanking the substituted amino acid on each side. For each patient, the 25-mer amino acid sequences were then evaluated through all HLA class I peptide-binding algorithms available in pVACtools to predict high affinity mutated (MT) (8–11-mer) peptides expected to bind to the patient’s HLA alleles. Matching WT sequences were also evaluated to calculate differences in binding affinities. Mutant peptides were prioritized by binding affinity (median IC50 value across multiple algorithms typically < 500 nm), sequence coverage, expression (of the gene transcript and mutant allele), variant allele fraction (preferring clonal variants to subclonal), and whether HLA anchor positions coincided with the mutation. For those candidates with a missense mutation in one of the HLA anchor positions, the fold change between mutant and wild-type peptides was used to prioritize candidates with a fold change > 1. Screening was performed for incidental matches with the wild-type proteome, and where appropriate peptides arising from known breast cancer driver genes were prioritized. This produced a high-confidence list of high-affinity HLA class I binding neoantigen candidates for experimental validation. The list was discussed at a weekly Immunogenomics Tumor Board meeting that included physician scientists, genome scientists, and immunologists. The top neoantigen candidates were selected for inclusion in each vaccine. Additional file 2: Table S1 contains all of the mutations that result in candidate neoantigens after application of the prediction algorithm with the mutations included in the patient’s vaccine highlighted in bold. The table indicates the relevant genes and amino acid changes and indicates the known cancer driver genes. Additional file 2: Table S2 indicates whether the annotated gene is found in a list of known cancer driver genes derived from large pan-cancer studies of mutational recurrence [55]. We also indicated how many driver and passenger mutated genes are incorporated into each vaccine.

### Neoantigen DNA vaccine design and manufacture

Polyepitope inserts encoding prioritized neoantigens and the sequence for Ub^mut^, a mutated (G76V) ubiquitin [56] fused to the N-terminus of the polyepitope construct were synthesized by Blue Heron Biotech (Bothell, WA) and subsequently cloned into the pING vector [57]. Plasmid DNA was amplified in *E. coli* DH5α (Blue Heron) and the transformed bacteria were shipped to the Biologic Therapy Core Facility (BTCF) at Washington University School of Medicine. Bacterial cultures were expanded at the BTCF followed by lysis and DNA extraction. Each DNA vaccine was vialed at a concentration of 2 mg/mL. Before release, each neoantigen DNA vaccine underwent rigorous product release testing to assure purity, identity, and sterility. The ability to express mRNA in mammalian cells was also confirmed. The results of the product release tests were documented in a Certificate of Analysis which was reviewed and approved by both the principal investigator and BTCF staff. Actual vaccine production from the time tissue was obtained to the approval of the Certificate of Analysis in the GMP Facility varied from 3 to 5 months.

### Peptides

Peptides for immune monitoring were obtained in lyophilized form at > 95% purity (Peptide 2.0 Inc., Chantilly, VA). Peptides were dissolved in sterile water or in 4% DMSO dependent on the amino acid sequence. Typically, three peptides of 15 to 16 amino acids in length overlapping by 11 amino acids were pooled to encompass the ~ 25 amino acid candidate neoantigen epitope encoded by the DNA vaccines.

### ELISpot assay

**Fig. 2:**
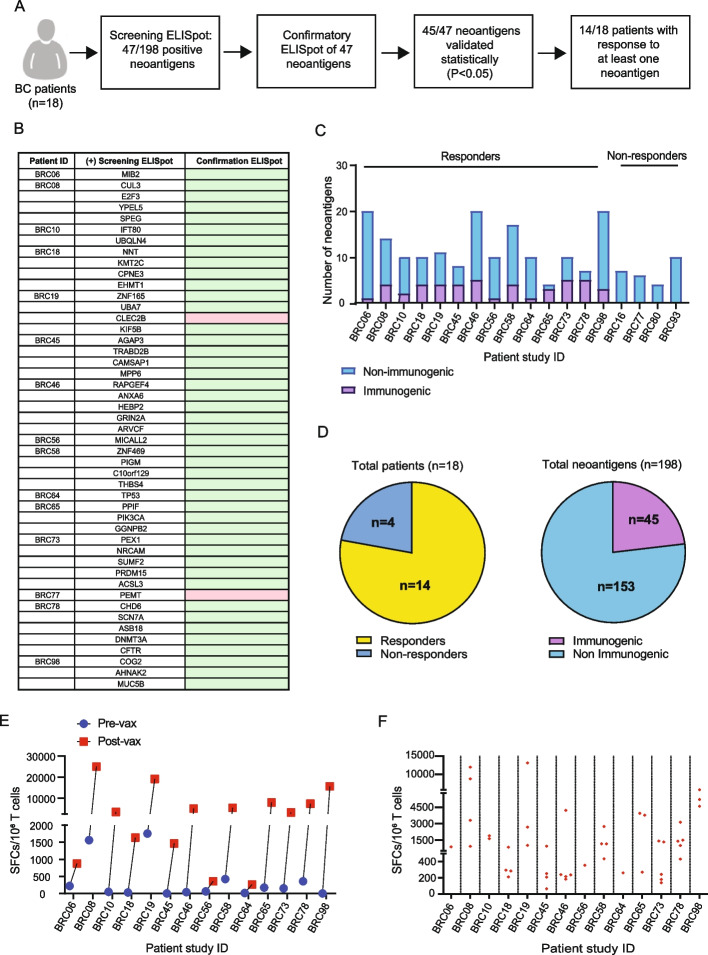
Neoantigen DNA vaccines induce neoantigen-specific immune responses. **A** Overview of ELISpot immune monitoring assays including a screening ELISpot assay followed by confirmatory ELISpot assay. The response to 45/47 neoantigens was statistically validated in the “confirmatory ELISpot.” Patients who responded to at least one neoantigen were considered to be responders. **B** List of all neoantigens conferring immune responses, as assessed by both screening and confirmatory IFN-γ ELISpot assay. Patient-derived PBMCs were stimulated with pooled candidate neoantigens for 12 days. On day 12, cells were harvested and stimulated in IFN-γ ELISpot assays (screening ELISpot) with autologous, irradiated PBMC pulsed with overlapping peptide pools of neoantigens or individual overlapping peptides. ELISpot assays were repeated for those neoantigens that elicited an immune response (confirmatory ELISpot), and confirmatory ELISpot demonstrated that almost all neoantigens were statically validated using Student’s *t*-test (indicated by the green color), with the exception of two neoantigens (pink color). **C** Breakdown of the number of immunogenic vs non-immunogenic neoantigens per patient. **D** The number of patients responding to at least one neoantigen (responders), and the number of immunogenic vs non-immunogenic antigens based on a total of 198 candidate neoantigens for all patients. **E** The cumulative number of spot-forming cells for all neoantigens per patient comparing pre- and post-vaccination analysis of T cells after 12-days stimulation with neoantigen peptides (*P* < 0.05 for all patients). **F** The number of SFC for each immunogenic neoantigen post vaccination and culture

2 × 10^5^ PBMCs were plated in each well of a 96-well round bottom plate with RPMI (with 5% human serum, 10 units/mL penicillin–streptomycin, 10 mM HEPES buffer, 2 mM L-glutamine, 1 × non-essential amino acid). Pooled overlapping peptides corresponding to prioritized neoantigens were used to stimulate PBMCs at 25 µM, and 50 U/mL IL2 was added every 2 days. Control PBMCs were stimulated with peptides corresponding to known viral antigens. On day 12, the peptide-specific immune reactivity of the T cells was determined by IFN-γ ELISpot assay as follows. Cultured T cells were stimulated with peptide-pulsed, irradiated autologous PBMC in the ELISpot plate followed by 20 h of incubation at 37 °C. Developed spots were counted in an ELISpot reader (C.T.L., Shaker Heights, OH). Positive results were repeated at least once to confirm the results as indicated in Fig. 2B.

### Flow cytometry

PBMCs were stimulated with overlapping peptides for 12 days with IL2 expansion as described under ELISpot assay. On day 12, cultured cells were stimulated with peptide-pulsed, irradiated autologous PBMCs overnight with brefeldin A added during the last 3 h. Intracellular staining for IFN-γ was performed by following the protocol from BioLegend. The following anti-human monoclonal antibodies (mAb) were used for cell staining: live/dead AF488 (ThermoFisher Scientific, Waltham, MA), CD4-PerCP-Cy5.5 (clone: RPA-T4), CD8-PE (clone: HIT8a), IFN-γ-APC (clone B27). All antibodies were obtained from BD Bioscience (San Jose, CA). Samples were analyzed on a FACSCalibur (BD Biosciences, Franklin Lakes, NJ, USA), and data were analyzed using FlowJo v10.7.

### Tetramer staining

MHC-I tetramers were prepared by the Immune Monitoring Laboratory at the Bursky Center for Human Immunology and Immunotherapy, Washington University School of Medicine. Peptide-MHC complexes were multimerized by the addition of streptavidin APC conjugate (Invitrogen, Eugene, OR, USA) and PE streptavidin (BioLegend, CA) followed by adding D-biotin after incubation. Multimer and live/dead AF488 were used to stain peptide-stimulated T cells. Tetramer-positive live cells were sorted and sent for scTCRseq.

### Sample preparation and DNA sequencing for TCR

Peptides with demonstrated immunogenicity by ELISpot assay were used to stimulate PBMCs as described above under ELISpot. Control PBMCs were stimulated with irrelevant peptides or media only. On day 12, cells were harvested and genomic DNA was extracted and purified from cells using the QIAGEN Blood and Tissue Kit (Qiagen, Germantown, MD). TCRVβ CDR3 regions were amplified and sequenced using ImmunoSEQ (Adaptive Biotechnologies, Seattle, WA). Data [77] were analyzed with ImmunoSEQ software and GraphPad Prism 9. Alternatively,

RNA was extracted instead of DNA using the RNeasy Plus Kit (Qiagen, Germantown, MD). Bulk RNA was stored in nuclease-free water at − 80 °C until subsequent use. cDNA libraries of each sample’s TCR Vα and Vβ chains were prepared using an amplification bias-controlled multiplex PCR using the SMART-Seq Human TCR (with UMIs) kit (Takara Bio, San Jose, CA) according to the manufacturer’s instructions. Sequencing was performed on NovaSeq6000 or NovaSeq X Plus instruments (Illumina, San Diego, CA). Bulk TCR sequencing data generated with the SMART-Seq Human TCR kit were processed using the Cogent NGS Immune Profiler software v1.6 (Takara Bio, San Jose, CA) with default parameters and receptor type argument (-r) “TCRv2” to carry out UMI based calling and quantification of clonotypes. Analysis of clonotype frequencies was restricted to TRB CDR3 sequences.

### ScTCR-seq

In parallel with bulk TCR sequencing, cultured T cells were first incubated with TotalSeqC (Biolegend, San Diego, CA) using barcoded anti-CD3 (clone UCTH1), anti-CD4 (clone SK3), and anti-CD8 (clone SK1), and tetramer. Barcoded samples were subsequently prepared for single-cell sequencing with Illumina10 × Genomics processing. 5′ 10 × sc/snRNAseq (GEX) library was generated with T cell V(D)J enrichment. For BRC58, FASTQ files [77] were aligned to GRCh38–2020 and GRCh38-alts-ensembl- 5.0.0 references provided by 10X Genomics, using Cellranger v7.0.1. For BRC78, FASTQ files were aligned to GRCh38–2020 and GRCh38-alts-ensembl- 7.0.1. All data was processed through Seurat v4.3 [58]. Barcodes with fewer than 100 features or more than 10% mitochondrial genes were removed. Further filtering was done to remove low-quality clusters (based on high mitochondrial reads, low nFeatures, and low nCounts), and analysis was restricted to clusters with CD3D expression. For BRC78, clusters were classified as CD4 or CD8, or unclassified T cells based on CD4 and CD8A gene expression. TCR clonotypes were added to the Seurat object using scRepertoire (v1.7.1) [59]. Clonotypes where only the alpha or beta CDR3 was detected were merged with clonotypes where both CDR3s were detected if either the alpha or beta CDR3 matched.

TCR clones, human PBMC transduction, and TCR-engineered T cell functional validation. scTCR-seq identified PIGM-specific TCR α and β chains of the PIGM-specific TCR were generated by PCR and cloned into a modified version of pLL3.7 (Addgene, Watertown, MA) followed by insertion of a GFP gene. The resulting plasmid pLl1-TRAV19J28C-P2A-TRBC29DJ1-2C1-P2A-GFP (hereafter referred to as PIGM-TCR-GFP) was transfected into 293 Tcells with pMD2.G and psPAX2 (Addgene, Watertown, MA) to generate recombinant lentiviruses. 5 × 10^6^ 293T cells were pre-seeded onto a 6-well plate and transfected with 12 µg PIGM- lentiviral DNA [60]. Viral supernatants were harvested, passed through a 0.45-µM filter, and concentrated by ultracentrifugation at 20,000 rpm for 2 h. Virus pellets were resuspended in T cell culture medium, and viral titers were determined by infecting Jurkat cells with serially diluted doses of virus. For transduction of human T cells, PBMCs were first activated by PHA for 48 h, and then transduced with the concentrated lentiviral supernatant with a multiplicity of infection (MOI) of 10–15 in a total volume of 0.2 mL T cell medium containing 8 µg/mL polybrene (Sigma), and then spun at 1000 × g for 2 h at room temperature. Transduction efficiency was analyzed at 3 or 4 days post-transduction, and the cells were evaluated by flow cytometry for GFP expression. PIGM-TCR-GFP transduced and un-transduced T cells were used to assess recognition of PIGM peptide by INF-γ ELISpot.

### Statistical analyses

The data analysis for this study was descriptive in nature. Neoantigen-specific immune responses assessed by ELISpot assay were analyzed using Student’s *t*-test to compare baseline and post-vaccination PBMC samples. Recurrence-free survival (RFS) was defined as time from the first injection of neoantigen DNA vaccine to the date of relapse or death, whichever occurred first. Those patients alive and relapse-free were censored at the date of the last contact. The distribution of RFS was estimated using the Kaplan–Meier product-limit method and compared by log-rank test. All the analyses were performed using GraphPad Prism software (GraphPad, San Diego, CA, USA) or SAS 9.4 (SAS Institutes, Cary, NC).

## Results

### Treatment with personalized neoantigen DNA vaccines is feasible

Neoantigen DNA vaccines were designed and manufactured while subjects underwent standard therapy (Fig. 1A). Tumor RNA sequencing (using cDNA capture) was performed to assess the expression of somatic mutations. After completion of adjuvant therapy, subjects received three neoantigen DNA vaccinations via electroporation at monthly intervals. A total of 35 patients with TNBC consented to the trial (Fig. 1B). 17 subjects were ineligible and/or not treated for the following reasons: complete pathologic response to standard of care neoadjuvant therapy (*n* = 6), DCIS only in pathology (*n* = 1), insufficient tumor tissue (*n* = 3), patient preference (*n* = 4), or disease recurrence (*n* = 3). Neoantigen DNA vaccines were administered to 18 subjects (Fig. 1B, Table 1).

**Table 1:** Patient baseline characteristics and immune and clinical responses to neoantigen DNA vaccination

Patient ID	Age (years)	Race	Stage	Treatment		No. of epitopes in vaccine	Neoantigen immune response	Recurrence after vaccine (Y/N)
				**Neoadjuvant**	**Adjuvant**			
BRC06	50	White	cT2N0M0	TC	AC	20	Y	N
BRC08	56	White	cT2N0M0	TP	RT	14	Y	N
BRC10	61	AA	cT2N0M0	TC	RT	10	Y	N
BRC16	60	AA	cT3N0M0	TC	Capecitabine	7	N	Y
BRC18	36	White	cT2-3N0M0	ACT	Metformin	10	Y	N
BRC19	53	AA	cT2N1M0	TC	RT	11	Y	N
BRC45	68	AA	cT1N1M0	TC	RT	8	Y	N
BRC46	68	AA	cT2N1bM0	TP	RT/EC	20	Y	N
BRC56	38	White	T3N0M0	ACTP	RT	10	Y	N
BRC58	53	White	cT2N1M0	TP	RT	17	Y	N
BRC64	52	AA	cT3N1M0	ACT	RT	10	Y	N
BRC65	51	White	cT2N1M0	ACT	RT/capecitabine	4	Y	N
BRC73	63	White	cT2N0M0	ACTP	RT	10	Y	N
BRC77	43	White	T3N1M0	TC	RT	6	N	Y
BRC78	33	White	cT1N1M0	ACT	RT/GC	7	Y	N
BRC80	69	White	cT2N1M0	TC	RT/capecitabine	4	N	N
BRC93	54	White	cT2N1M0	ACTP	RT/capecitabine	10	N	N
BRC98	49	White	cT2N1M0	TP	RT	20	N	N

The full names of medications used in neoadjuvant and adjuvant treatments, listed in columns 5 and 6, are as follows: *TC* Taxotere and cyclophosphamide, *TP* Taxotere, trastuzumab, and pertuzumab, *ACT* Adriamycin, cyclophosphamide, and taxane, *RT* Radiation therapy, *GC* Gemcitabine and cisplatin, *ACTP* Adriamycin, cyclophosphamide, taxane, and pertuzumab, *EC* Epirubicin and cyclophosphamide, *AC* Adriamycin and cyclophosphamide

### Neoantigen identification and vaccine design

Tumor RNA sequencing [77] showed a median of 21.5 mutations resulting in altered protein/amino acid sequences being expressed per patient sample. Of these, a median of 8 mutations gave rise to candidate neoantigens with a predicted binding score < 500 nM (Additional file 1: Fig. S1A). The median number of neoantigens ultimately included in the neoantigen DNA vaccines was 10 (range 4–20, Additional file 1: Fig. S1A, Table 1) and reflects the decision to relax binding and expression thresholds slightly to identify additional neoantigens for inclusion in some cases. 97% of the candidate neoantigens were the result of missense mutations, with the remaining neoantigens being the result of insertion/deletion or frameshift mutations (Additional file 1: Fig. S1B). Mutations in TP53 were common, and candidate neoantigens related to TP53 mutations were present in 14/18 subjects (78%), although the location of the TP53 mutations differed among subjects (Additional file 1: Fig. S1C). Mutations in other genes that are commonly found in TNBC (such as SOX17, KMT2D, and PIK3R1) were much less frequently observed (≤ 17%). In only one patient, BRC58, was a pathogenic frameshift deletion mutation observed in BRCA1/2. Of note, the data in Additional file 1: Fig. S1B includes genetic alterations in cancer-related genes ordered by frequency of occurrence. Not all genetic alterations from such genes met the criteria for inclusion in the neoantigen DNA vaccines.

### Induction of neoantigen-specific immune responses

Immune monitoring was performed in vaccinated patients using PBMC collected at baseline and post-vaccination. A tiered process was followed in which all PBMC samples were first screened for neoantigen-specific immune responses by IFNγ ELISpot (Fig. 2A). Immune monitoring was conducted in an unbiased manner by using overlapping peptides corresponding to each neoantigen included in the vaccine (typically three peptides of 15 to 16 amino acids overlapping by 11 amino acids). Positive controls included a pool of viral peptides (Additional file 2: Table S3). Neoantigen-specific responses were assessed in baseline and post-vaccination PBMC after in vitro culture for 12 days with the overlapping peptides (OP), followed by IFNγ ELISpot assay. Neoantigens eliciting a response in the initial screening ELISpot were retested by confirmatory ELISpot. The response to the neoantigens was statistically validated in the confirmatory ELISpot. Of the 47 neoantigens that induced an initial ELISpot response in post-vaccination PBMC, 45 neoantigens were confirmed to be immunogenic in the repeat ELISpot (Fig. 2B). Patients who responded to at least one neoantigen were considered to be responders. Overall, 14 of the 18 patients showed a response to at least one neoantigen (Fig. 2C, D), and 45 (23%) of the 198 total neoantigens from all 18 patients conferred immunogenicity. Given that the ELISpot analyses were performed after in vitro culture, it is hard to definitively discriminate between de novo and expanded responses. For 10/14 patients the cumulative number of SFCs pre-vaccination was less than 250 (with SFCs = 0 in 7/14 patients), but post-vaccination and culture the cumulative spot numbers per million T cells ranged from 250 to over 20,000 (Fig. 2E). Broken down by individual neoantigen, the magnitude of the response to individual neoantigens varied widely post vaccination and culture (Fig. 2F).

**Fig. 3:**
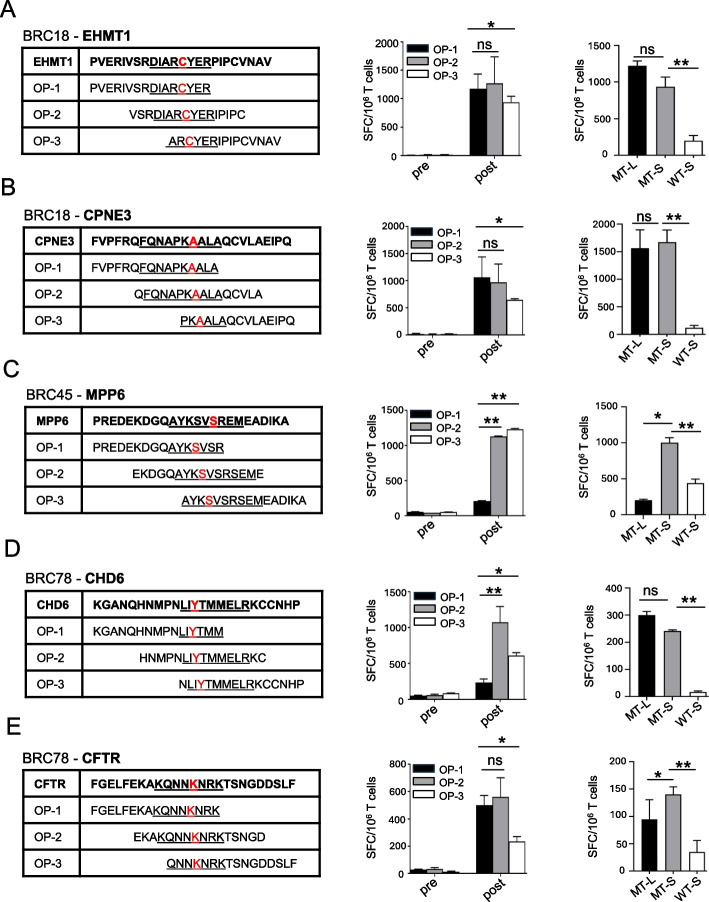
Specificity of immune responses to predicted candidate neoantigens before and after vaccination. PBMC at baseline (pre-vax) and after vaccination (2 weeks post vaccination) were stimulated with pooled OP encoding two candidate neoantigens for 12 days. PBMCs were selected based on the relative strength of the immune response (as assessed by ELISpot), the predicted binding of the corresponding minimal epitope, and the availability of PBMC. For each patient, T cell IFN-γ ELISpot assays against pooled (MT-L) and individual OP (OP1–3), as well as the minimal predicted neoantigen (MT-S) and matching wild type peptide (WT-S) were performed on day 12 by co-culturing stimulated T cells overnight with autologous, irradiated PBMC pulsed with peptide. The sequence of individual OP from representative patients is listed with the minimal predicted MHC class I epitope underlined and the missense mutation indicated in red. Panels **A**–**E** show IFN-γ secretion ELISpot assays for patients BRC18 (**A**, **B**), patient BRC45 (**C**), and patient BRC78 (**D**, **E**). Different OPs are indicated in color in the bar graphs (black: OP-1; gray: OP-2; white: OP-3). The negative controls in the ELISpot assays included responder T cells cultured with no peptide (the number of spot-forming cells per 10^6^ cells was 10–120). The background without peptide was subtracted from the experimental condition in each case. Data are presented as means ± SEM (*n* = 2–3 wells per peptide in ELISpot assay) and are representative of three independent experiments. Samples were compared using unpaired, Student’s *t*-test (*, *P* < 0.05; **, *P* < 0.01; ns, no significant difference); SFC, spot-forming cells. ELISpot experiments were performed in duplicate or triplicate wells per condition

Epitope deconvolution analysis using individual long peptides provided additional insight into the specificity of neoantigen-specific T cell responses. While very little immune reactivity was observed in pre-vaccination T cells after in vitro sensitization, T cells from post-vaccination blood draws showed substantial but differential activity to OPs after culture. Specifically, in some instances, e.g., Fig. 3C, E and Additional file 1: Fig. S2A, the highest T cell reactivity was observed against the OP2 that included the entire predicted MHC class I epitope, whereas OPs incorporating only part of the predicted minimal epitope were poorly recognized. However, in other cases, e.g., Fig. 3A, B and Additional file 1: Fig. S2B, the responses to individual OPs were more balanced, suggesting that the response was a mixed response against more than the minimal predicted epitope, mediated by perhaps both CD8 and CD4 T cells. T cell responses were also tested against minimal peptides corresponding to the predicted mutant and matching wild type MHC class I epitope. The minimal mutant peptides elicited equal or better reactivity than the 15/16-mer OP, whereas the matching wild-type peptide generally elicited little to no reactivity (Fig. 3). In some cases, the response to the predicted mutant MHC class I epitope was trending lower than that against the OP (Fig. 3A, D), suggesting the OP encoded additional epitopes outside of the minimal predicted epitope. This may suggest that the predictions failed to prioritize the most immunogenic MHC class I epitope. Alternatively, it could indicate that the response to the OPs is a mixed response mediated by both CD8 and CD4 T cells.

**Fig. 4:**
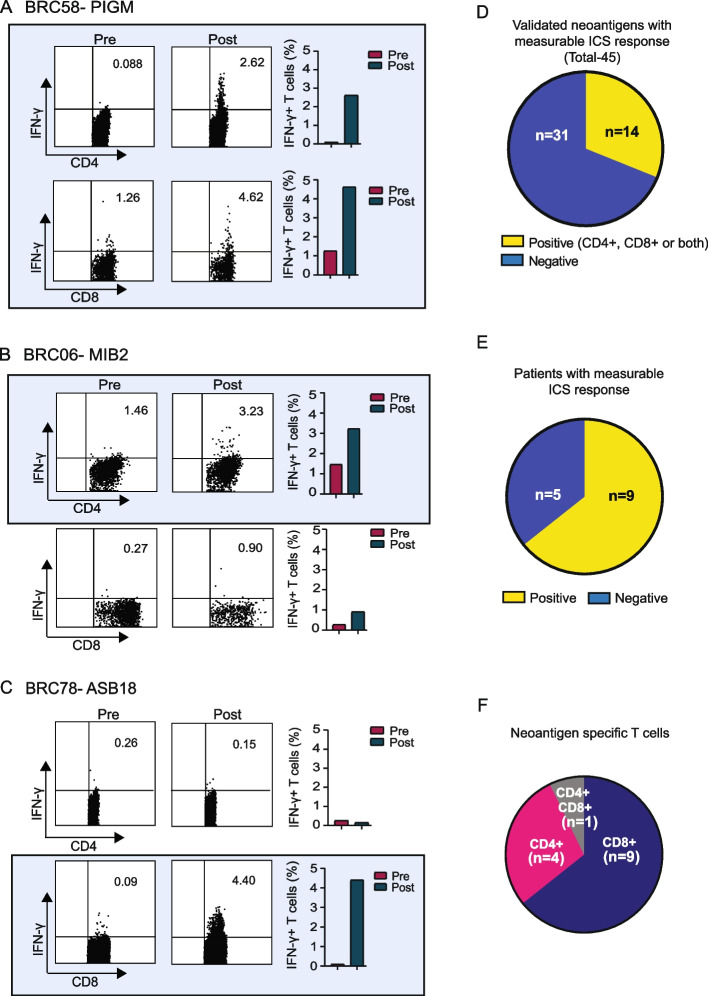
Neoantigen vaccines elicit both CD4 and CD8 T cell responses. (**A**–**C** Examples of increased intracellular IFN-γ production after vaccination and in vitro culture with neoantigen peptide in three patients. Bar graphs reflect the percent IFN-γ + cells per T cell subset and blood draw. **D** Breakdown of positive vs negative neoantigens by ICS. **E** Breakdown of ICS data by patients and **F** T cell subset with regard to immunogenicity. Increases between pre- and post-vaccination are considered positive when a twofold or greater increase is observed in percent positive cells, with a minimum percent positive of ≥ 1% in the post-vaccination sample. All positive neoantigen responses are indicated by blue shading

Analysis of intracellular IFN-γ expression by flow cytometry after neoantigen stimulation showed specific responses in both CD4 and CD8 T cells (Fig. 4 and Additional file 1: Fig. S3). We considered a neoantigen to be positive on the ICS assay if there was at least a twofold increase in the percentage of ICS-positive cells from pre- to post-vaccination, and a minimum of ≥ 1% ICS-positive cells in the post-vaccination sample. Using these criteria, 14 of 45 validated neoantigens were positive. Of note, we consider these criteria for defining a positive result to be rigorous; the number of positive neoantigens increases from 14 to 30 if only the twofold increase in the percentage of ICS-positive cells from pre- to post-vaccination criteria is used without a required minimum value of 1% ICS-positive cells.

### Paired scTCR-seq and bulk TCR-seq demonstrate the expansion of neoantigen-specific TCRs following vaccination

**Fig. 5:**
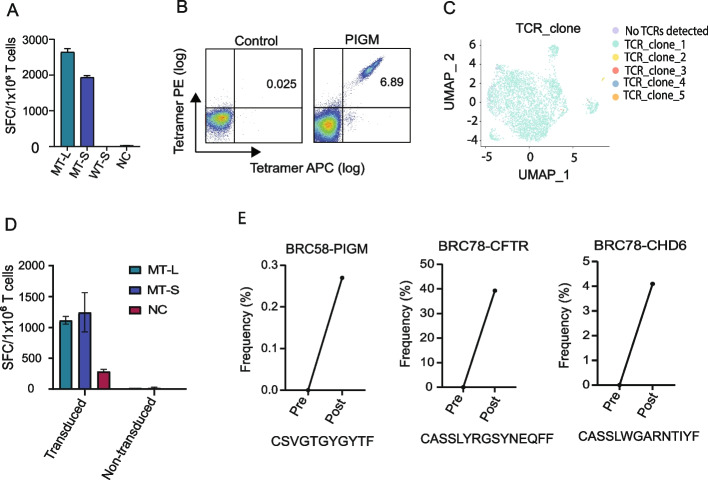
T cell receptor sequencing analysis confirms neoantigen vaccination expands neoantigen-specific T cells. **A** PBMCs from patient BRC58 were stimulated with pooled PIGM OPs for 12 days. T cell IFN-γ ELISpot assay against pooled OPs (MT-L), as well as the minimal predicted neoantigen (MT-S) and matching wild-type peptide (WT-S) were performed on day 12 by co-culturing stimulated T cells overnight with autologous, irradiated PBMC pulsed with peptide. Cells were rescued from ELISpot plates and were continuously cultured for another 12 days for subsequent tetramer-based TCR analysis. **B** Tetramer staining of cells. Tetramer + cells were sorted and subjected to scTCR-seq. **C** UMAP plot of tetramer-sorted cells, showing an almost monoclonal population. TCR genes of the dominant TCR clone were transduced into naïve PBMC of BRC58, activated with PHA for 48 h. Transduced cells were expanded for 96 h and tested for recognition of the PIGM neoantigen by ELISpot. **D** IFN-γ ELISpot data showing TCR-transduced but not untransduced cells recognize the PIGM neoantigen. NC refers to unpulsed PBMC. **E** Bulk TCR-seq was performed on cells collected before and 2 weeks post vaccination that were cultured with neoantigen as listed above for three patients. The CDR3 of the dominant TCRVβ is shown for each patient

To determine if vaccination with neoantigen DNA vaccines results in the expansion of neoantigen-specific T cell receptors, paired single-cell TCR-seq (scTCR-seq) and bulk TCR-seq was performed in three cases. Specifically, T cells from BRC78 stimulated with neoantigens CHD6 or CFTR (Fig. 3D, E), and T cells from BRC58 stimulated with neoantigen PIGM (Fig. 5A) were expanded in vitro and then subjected to scTCR-seq with identification of both TCRα and β genes Additional file 1: Fig. S4. Tetramer synthesis was attempted for all three neoantigens but was only successful for PIGM. Using the tetramer, PIGM-stimulated T cells from BRC58 were sorted followed by scTCR-seq analysis (Fig. 5B, C). A single dominant clone was observed. This PIGM-specific TCR was subsequently expressed in autologous PBMC through transduction by a lentivirus encoding the TCR genes. Transduced PBMC, but not untransduced PBMC, produced IFN-γ following PIGM stimulation as assessed by ELISpot (Fig. 5D). This confirms that the TCR identified by scTCR-seq is specific for the PIGM peptide. In the case of the CHD6 and CFTR neoantigens, scTCR-seq was performed without tetramer-based sorting. For these two neoantigens, a very limited repertoire of TCRs was identified.

Bulk TCR-seq was also performed to determine if the neoantigen-specific TCRs identified by scTCR-seq expanded following vaccination. Bulk TCR-seq was performed after in vitro stimulation of PBMC with the PIGM, CHD6, and CFTR neoantigens. As controls, PBMCs were cultured in a medium without peptide or with neoantigen peptides included in the patient’s vaccine that did not confer an immune response (data not shown). Bulk TCR-seq analyses of these pre- and post-vaccination short-term cultures demonstrated a significant expansion of the dominant TCR Vꞵ clonotypes identified by scTCR-seq (Fig. 5E), demonstrating that vaccination results in expansion of neoantigen-specific TCRs.

Additional bulk TCR-seq analyses were performed for 10 immunogenic neoantigens across 6 patients (Additional file 1: Fig. S5). These analyses confirm expansion of specific TCR clonotypes following vaccination suggesting that additional neoantigen-specific clonotypes were expanded. In some cases (e.g., EHMT1), multiple clones increased in frequency suggestive of an oligoclonal response, whereas in other cases (ZNF165 and CPNE3), the response appeared more monoclonal (Additional file 1: Fig. S5). We are in the process of validating the specificity of these TCR clonotypes.

### Evaluation of clinical outcomes after neoantigen DNA vaccination

**Fig. 6:**
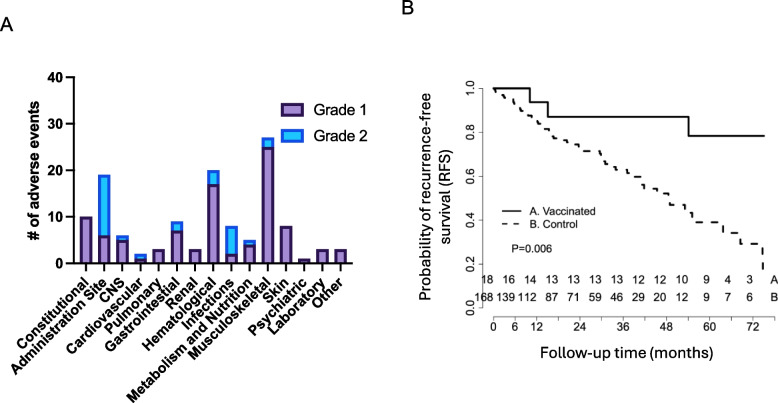
Neoantigen vaccine treatment is safe and prolongs recurrence-free survival compared to historical controls. **A** Adverse events (grade 1 and 2 toxicity) are depicted by type and number among all vaccinated patients. **B** Kaplan–Meier survival curve of patients vaccinated with a personalized neoantigen DNA vaccine compared to non-vaccinated historical control patients

Vaccination was well-tolerated in general with only one grade 3 adverse event (hypertension) with the remaining adverse events being grade 2 related to pain at the injection site (13 grade 2 events out of 28 total), or grade 1 mostly related to myalgia (Fig. 6A). While not powered to formally assess clinical response, patients treated with neoantigen DNA vaccines had excellent clinical outcomes with very few experiencing disease recurrence. We compared clinical outcomes between patients treated with neoantigen DNA vaccines in this trial to institutional historical controls (Fig. 6B). Vaccinated patients were compared to a consecutive series of TNBC patients treated at Washington University School of Medicine [61]. Out of 117 patients treated with neoadjuvant chemotherapy, 60 in this series were selected as controls based on Stage II/III disease, and relapse-free status greater than 4 months after surgery. The 0 time point of the survival curve in the control group was reset as 4 months after surgery to account for the time required to design and manufacture neoantigen DNA vaccines. After 36 months of follow-up, RFS was 87.5 (95% CI: 72.7–100%) in vaccinated patients, compared to 49% (95% CI: 36.4–65.9%) in the institutional TNBC controls (*p* = 0.011).

## Discussion

Preclinical studies and early-phase clinical trials have established DNA vaccines as a safe, flexible, and robust vaccine platform [62, 63]. In addition to a remarkable safety profile, other advantages of the DNA vaccine platform include low cost, relative ease of manufacture compared to other vaccine platforms, and design flexibility which allows the inclusion of multiple target antigens. We have recently developed and optimized a neoantigen DNA vaccine platform [5]. We demonstrated that polyepitope neoantigen constructs expressing multiple long (> 20-mer) neoantigen epitopes fused with a mutant form of ubiquitin are able to induce antitumor immune responses in preclinical models. The current study is the first completed clinical trial that leverages this neoantigen DNA vaccine platform to target human cancer neoantigens. While the first neoantigen-based cancer vaccine clinical trials focused on ex vivo-pulsed dendritic cells, synthetic long peptides, or DNA as delivery vehicles, current clinical trials reflect additional platforms such as mRNA, viral vector, and other platforms (reviewed in [15, 64]. It remains to be seen which platform, if any, is superior.

The current study is also the first completed neoantigen vaccine study to focus exclusively on patients with TNBC, confirming the feasibility and potential of neoantigen vaccine therapy in this patient population. Prior studies have focused on high mutational burden cancers such as melanoma, non-small cell lung cancer, bladder cancer, and glioblastoma. Our trial is unique in terms of targeting breast cancer, characterized as a tumor with a relatively low mutational burden [65]. Despite this relative paucity of mutations, we were able to successfully identify between 4 and 20 cancer neoantigens for each TNBC patient and produced neoantigen-specific immune responses in the majority of patients in our trial. Using ELISpot as the primary readout, neoantigen-specific immune responses were detected in 14 of 18 vaccinated patients. Of note, validation of neoantigens by ICS analysis failed to confirm immunogenicity for multiple neoantigens, perhaps related to lower assay sensitivity of the ICS assay, combined with suboptimal cell condition. Overall, our data suggests a clinical benefit as vaccinated patients had improved overall survival compared to institutional historical controls.

While neoadjuvant therapy results in a pCR rate of around 50%, there are limited treatment options for TNBC, which is associated with a more aggressive course and has a greater likelihood of recurrence after surgery [18]. However, there is a paucity of systemic therapies available beyond chemotherapy due to TNBC’s insensitivity to hormonal therapy and/or targeted therapies. Administration of a neoantigen vaccine in the adjuvant setting is associated with several advantages. First, biopsies or surgical specimens can be used for sequencing and neoantigen identification. Second, there is a window to design and manufacture the vaccine as the patient recovers from surgery and undergoes adjuvant radiation therapy. Third, there is evidence to suggest that cancer vaccines will be most successful in the adjuvant setting, avoiding the tumor-induced regulatory networks and immunosuppression that is often present with metastatic disease. Thus, neoantigen vaccines for TNBC in the adjuvant setting are not only practical, but fill an unmet clinical need.

The design of these vaccines was made possible by the pVACtools suite of computational methods for the prediction of cancer neoantigens [66]. The Tumor Neoantigen Selection Alliance (TESLA) recently compared 25 prediction algorithms and identified factors such as neoantigen MHC binding affinity, half-life, expression level, and level of foreignness as important predictors of neoantigen immunogenicity [67]. pVACtools incorporates all of these key factors identified by the TESLA consortium which likely contributed to the success rate of the vaccines in this trial.

A potential shortcoming of pVACtools as used in this trial is the emphasis on MHC class I binding given the importance of CD4 cells in reprogramming the tumor microenvironment and promoting antitumor immunity [7]. Although our neoantigen predictions prioritized binding affinity to MHC class I, we detected neoantigen-specific CD4 T cell responses in some patients. The neoantigen DNA vaccine polyepitope inserts were designed to express long peptides, 20–30 amino acids in length. Peptides of this length are preferentially processed and presented by antigen-presenting cells [68], but also have the ability to bind both MHC class I and II molecules, with the ability to activate both CD8 and/or CD4 T cells. There is evidence that CD4 T cells can help CD8 T cell priming by licensing cDC1 via the CD40/CD40L interaction [69], and can help prevent CD8 T exhaustion [3, 70]. Interestingly, while mutated TP53 gave rise to candidate neoantigens in 6/18 patients, TP53 immunogenicity was demonstrated in only one patient (BRC64, Fig. 2B), suggesting a lack of immunogenicity or exhaustion of T cell responses to this antigen. CD4 T cells also have effector roles in the tumor microenvironment including direct cytotoxicity [71], cytokine secretion, and NK cell activation. There is also emerging evidence that many tumors have undergone MHC class I loss, but may still be susceptible to CD4 T cell-mediated immunity [3]. With recent improvements in the predictive power of MHC class II algorithms, and/or T cell-based screening of candidates prior to vaccination to assess the immunogenicity of candidates, future studies may be able to generate even more effective vaccines.

Our study focused on TNBC patients who had persistent disease following neoadjuvant chemotherapy, a group with significantly worse survival compared to patients with complete pathologic response or to other breast cancer subtypes [72]. While not designed to evaluate clinical outcomes, there were only 2 recurrences in the cohort of 18 vaccinated patients. This metric, coupled with the durable response measured by RFS is significantly better than institutional historical controls based on a study of consecutive TNBC patients seen at the WUSM between 2006 and 2010 [61]. In this institutional study, 87 patients had residual disease after neoadjuvant chemotherapy with survival greater than 4 months. Vaccinated patients had a 3-year recurrence-free survival of 87.5% (95% CI 72.7–100%) compared to 49% (95% CI 36.4–65.9%) in the control. It is important to recognize the limitations of comparison to historical controls. Important limitations include selection bias in the trial enrollment, differences in patient characteristics between the historical cohort and study patients, and evolution in the standard of care over time. Despite these limitations, we believe that the favorable clinical outcomes observed after neoantigen DNA vaccination provide strong support for further study of neoantigen DNA vaccines in TNBC patients.

Recent studies have demonstrated the importance of immune checkpoints and the tumor microenvironment in restraining antitumor immune responses. It is likely that in order to reach full therapeutic potential, cancer vaccines will need to be combined with other immune therapies such as immune checkpoint inhibition (ICI). It is important to note in this context that unlike in other studies [14, 73–76]*,* we did not consistently detect ex vivo immune responses, and focused our analyses on PBMC after short-term in vitro culture. We note two important differences between the study reported here and these other studies. First, the frequency and timing of vaccination and related blood draws may have played a role in terms of assessing the peak response to vaccine. For example, Hu et al. [74] used a “prime” cluster of vaccinations (days 1, 4, 8, 15, and 22) with two booster vaccinations at days 50 and 78. In the current study, patients received vaccinations at days 1, 29, and 57. In preclinical studies, we have observed that a “prime” cluster of vaccinations results in a dramatic expansion of neoantigen-specific T cells followed by a contraction phase and transition to a memory response. For example, in studies where mice received DNA vaccinations at days 0, 3, and 6, the response peaks at days 11–12 with contraction and transition to a memory response. It is possible that blood draws for immune monitoring failed to capture the peak of the immune response. Second, the lack of ex vivo responses may be related to the fact that our vaccine strategy did not include checkpoint blockade, unlike in the quoted studies from Awad et al., Palmer et al., Ott et al., and Rojas et al. [14, 73, 75, 76]. We demonstrated in a preclinical model that anti-PD-L1 treatment is able to augment antitumor immunity mediated by DNA vaccine-induced neoantigen-specific immune responses [5], and we are currently testing neoantigen DNA vaccines in TNBC ± durvalumab (NCT03199040). In a related study, we are investigating the combination of nab-paclitaxel, durvalumab, and tremelimumab ± neoantigen synthetic long peptide vaccines in patients with metastatic TNBC (NCT03606967).

The primary objective of this clinical trial was to test the safety of polyepitope neoantigen DNA vaccines in patients with TNBC. We demonstrated that neoantigen DNA vaccines are safe and well-tolerated, with no significant adverse events. The neoantigen DNA vaccines were able to induce neoantigen-specific immune responses, with evidence of improved recurrence-free survival compared to an institutional historical control cohort.

## Conclusion

These results support further study of the neoantigen DNA vaccine platform in TNBC and other low mutation burden cancers.

## Supplementary Information

Additional file 1: GMED Supplementary figure (pdf): include all 5 supplementary figures with legends.

Additional file 2: Supplementary tables (excel): include 3 supplementary tables with legends.

Additional file 3: clinical trial protocol (pdf): 20155073 protocol 05-22-19.

Additional file 4: Consort check list: consort 2010 check list A (pdf): CONSORT 2010 checklist of information to include when reporting a randomised trial.

## Data Availability

All data are available in the main text or the supplementary materials. The sequencing data has been deposited in the Database of Genotypes and Phenotypes dbGaP under accession phs0002787. The deposited data includes the tumor/normal whole exome sequencing data, the tumor RNA sequencing data, the bulk TCR sequencing data, and the scTCR-seq data. https://www.ncbi.nlm.nih.gov/projects/gap/cgi-bin/study.cgi?study_id=phs002787.v1.p1 [77].
