# Supplementary material for: Neoantigen DNA vaccines are safe, feasible, and induce neoantigen-specific immune responses in triple-negative breast cancer patients
Source: Genome Med. 2024 Nov 14;16:131. doi: 10.1186/s13073-024-01388-3 (PMC11562513; doi:10.1186/s13073-024-01388-3)
Supplement: Supplementary file 1 — Additional file 1: GMED Supplementary figure (pdf): include all 5 supplementary figures with legends. [file 13073_2024_1388_MOESM1_ESM.pdf]

Fig. S1

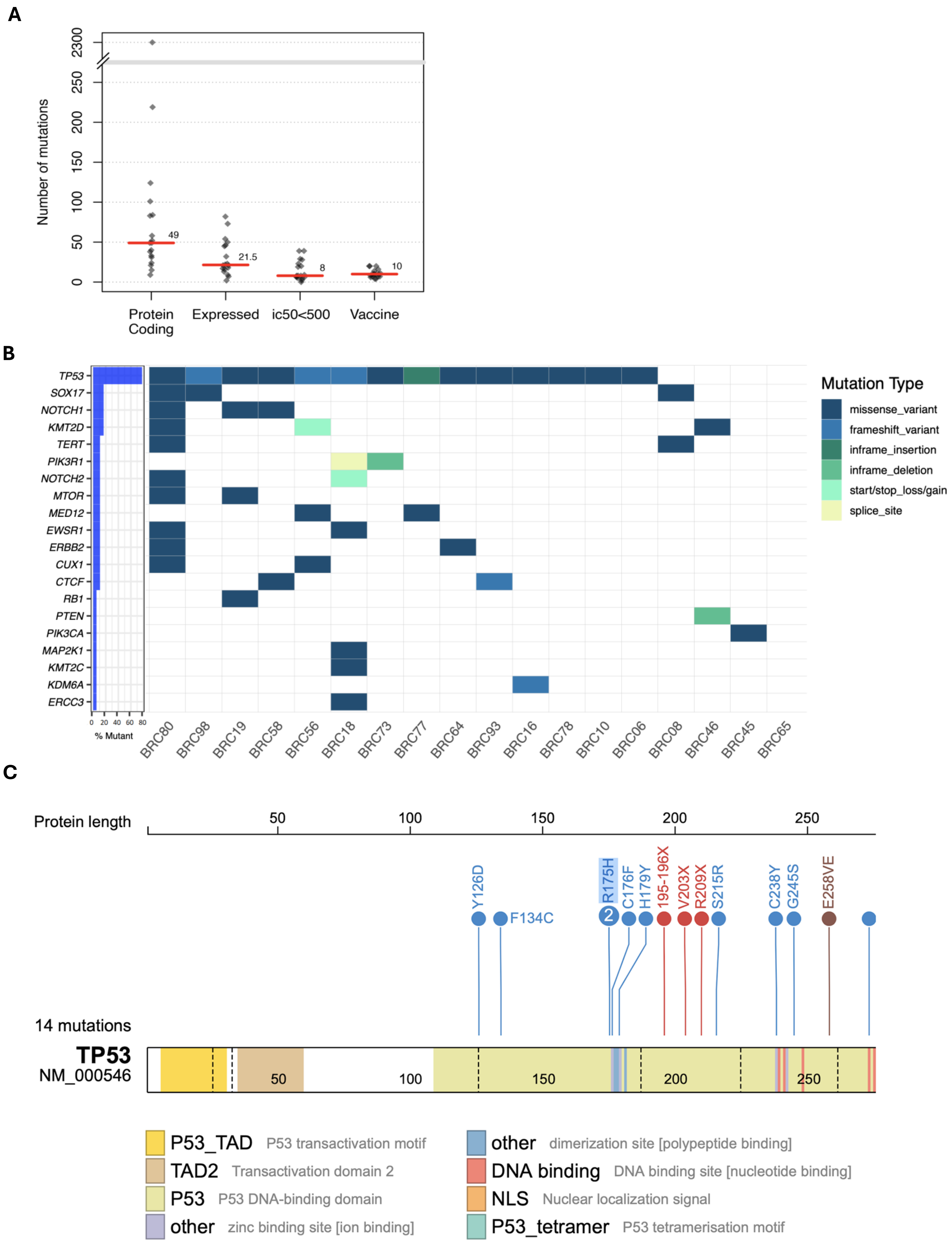
A
Number of mutations
49
21.5
8
10
Protein Coding
Expressed
ic50<500
Vaccine
B
Mutation Type
missense_variant
frameshift_variant
inframe_insertion
inframe_deletion
start/stop_loss/gain
splice_site
TP53
SOX17
NOTCH1
KMT2D
TERT
PIK3R1
NOTCH2
MTOR
MED12
EWSR1
ERBB2
CUX1
CTCF
RB1
PTEN
PIK3CA
MAP2K1
KMT2C
KDM6A
ERCC3
% Mutant
BRC80 BRC98 BRC19 BRC58 BRC56 BRC18 BRC73 BRC77 BRC64 BRC93 BRC16 BRC78 BRC10 BRC06 BRC08 BRC46 BRC45 BRC65
C
Protein length
14 mutations
Y126D
F134C
R175H
C176F
H179Y
195-196X
V203X
R209X
S215R
C238Y
G245S
E258VE
TP53
NM_000546
P53_TAD P53 transactivation motif
TAD2 Transactivation domain 2
P53 P53 DNA-binding domain
other zinc binding site [ion binding]
other dimerization site [polypeptide binding]
DNA binding DNA binding site [nucleotide binding]
NLS Nuclear localization signal
P53_tetramer P53 tetramerisation motif

Fig. S1. pVAC-seq analysis allows for prioritization of somatic mutations for inclusion in the neoantigen DNA vaccines. (A) Filtering plot: Counts of mutations at each stage of the mutation filtering process. Red lines and labels indicate median values. All protein-altering mutations were initially considered, followed by prioritization of those with variants expressed in the RNA, then with predicted binding affinities less than 500nm. The higher final number of mutations represented in the vaccines reflect the decision to relax binding and expression thresholds slightly to identify additional neoantigens for inclusion in some cases. (B) Waterfall plot: Mutational landscape of samples, showing protein-altering mutations in cancer-related genes. Genes are ordered by frequency and colored by alteration type. (C). Overview of mutations occurring within the TP53 gene, with the only recurrent mutation (R175H) indicated by a "2".

Fig. S2

A

BRC19 – ZNF165

**ZNF165**	**EKIESQRIISGRMSGYISEASGESQ**
OP-1	EKIESQRIISGRMSGY
OP-2	QRIISGRMSGYISEAS
OP-3	GRMSGYISEASGESQ

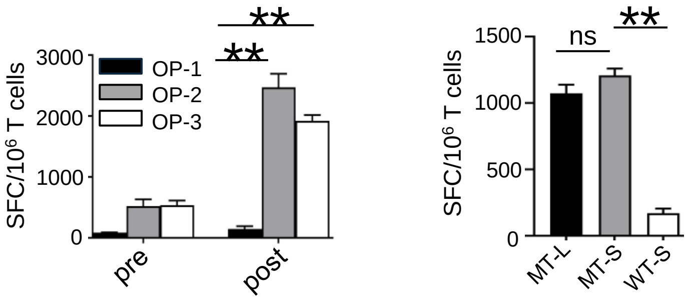

B

BRC19 – UBA7

**UBA7**	**DEELYSRQLYVLCSPAMQRIQGARV**
OP-1	DEELYSRQLYVLCSPA
OP-2	SRQLYVLCSPAMQRIQ
OP-3	VLCSPAMQRIQGARV

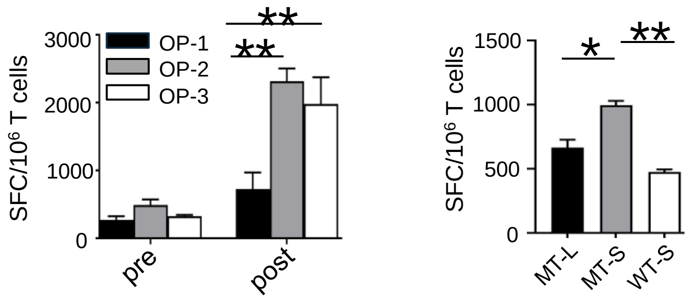

Fig. S2. Specificity of immune response to predicted candidate neoantigens before and after vaccination. PBMCs at baseline (pre-vax) and after vaccination (2 weeks post 3rd vax) were stimulated with pooled OP encoding two candidate neoantigens for 12 days. For each patient, T cell IFN-γ ELISpot assays against pooled (MT-L) and individual OPs (OP1-3), as well as the minimal predicted neoantigen (MT-S) and matching wild type peptide (WT-S) were performed on day 12 by co-culturing cells overnight with autologous, irradiated PBMC pulsed with peptide. (A) The sequences of individual OPs for patients BRC19 neoantigen ZNF165 and patient BRC19 neoantigen UBA17 are listed. (B) IFN-γ ELISpot data for pre- vs post-vaccination cells are shown. Different OPs are indicated in color (black: OP-1; gray: OP-2; white: OP-3). The negative control in the ELISpot assays included responder T cells cultured with no peptide (number of spot-forming cells per 10^6 cells was 10–120). The background (i.e. no peptide) was subtracted from the experimental condition in each case. Data are presented as means ± SEM (n =2 - 3 wells per peptide in ELISpot assay) and are representative of three independent experiments. Samples were compared using unpaired, Student's t-test (*, $P < 0.05$; **, $P < 0.01$); SFC, spot-forming cells. ELISpot experiments were performed in duplicate or triplicate wells per condition.

Supplementary figure 3

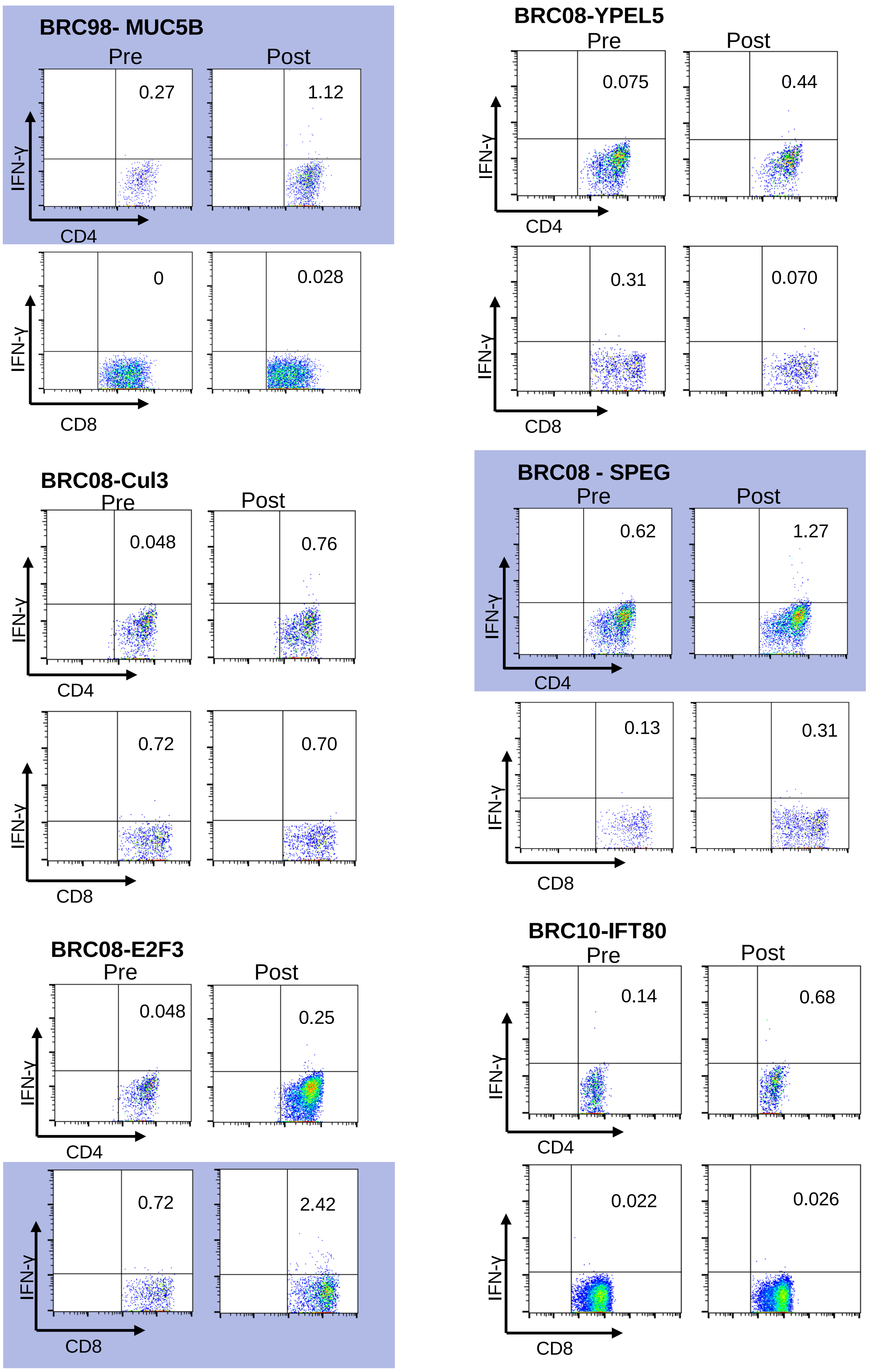

Supplementary figure 3-cont.

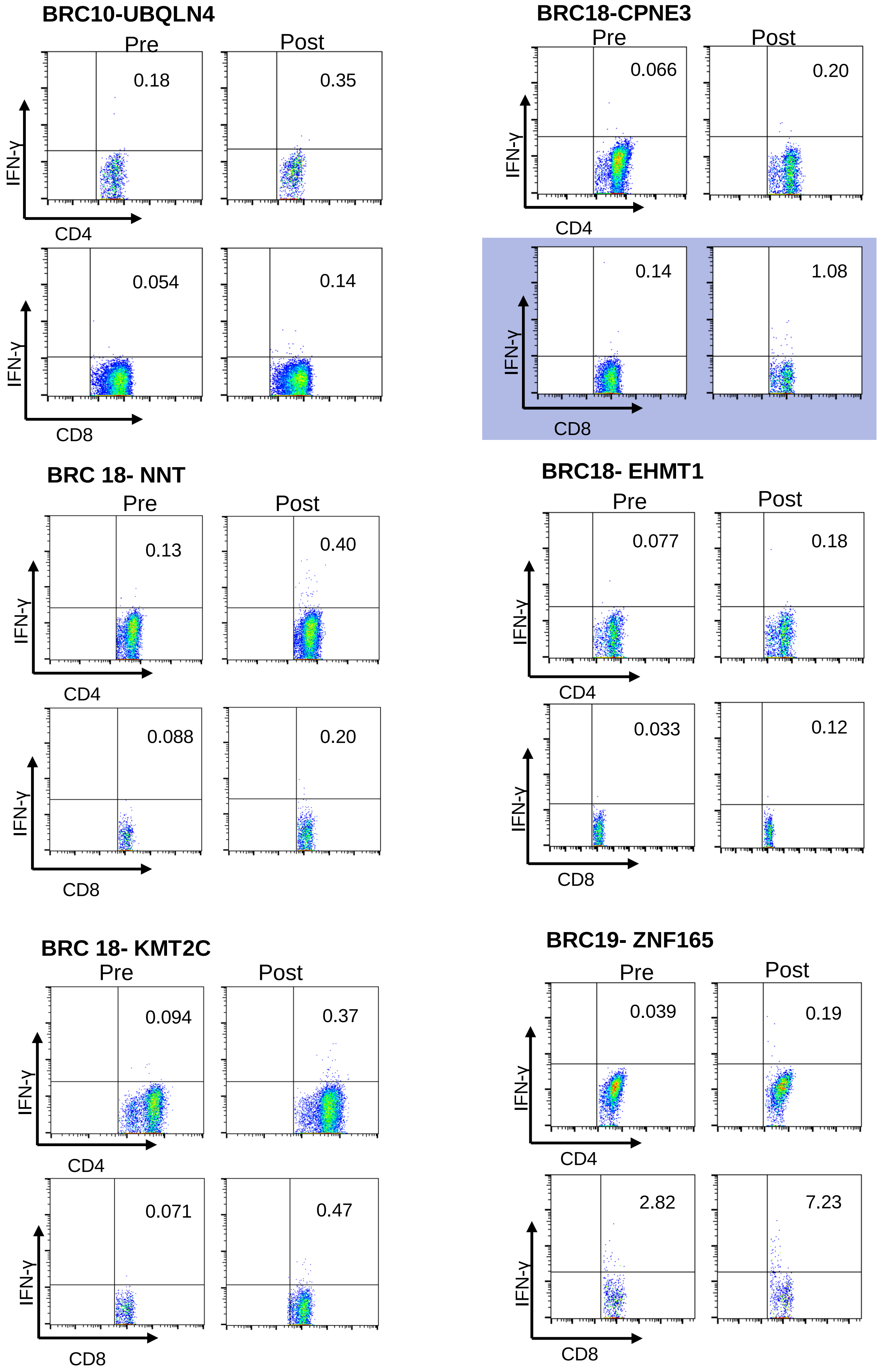

Supplementary figure 3-cont.

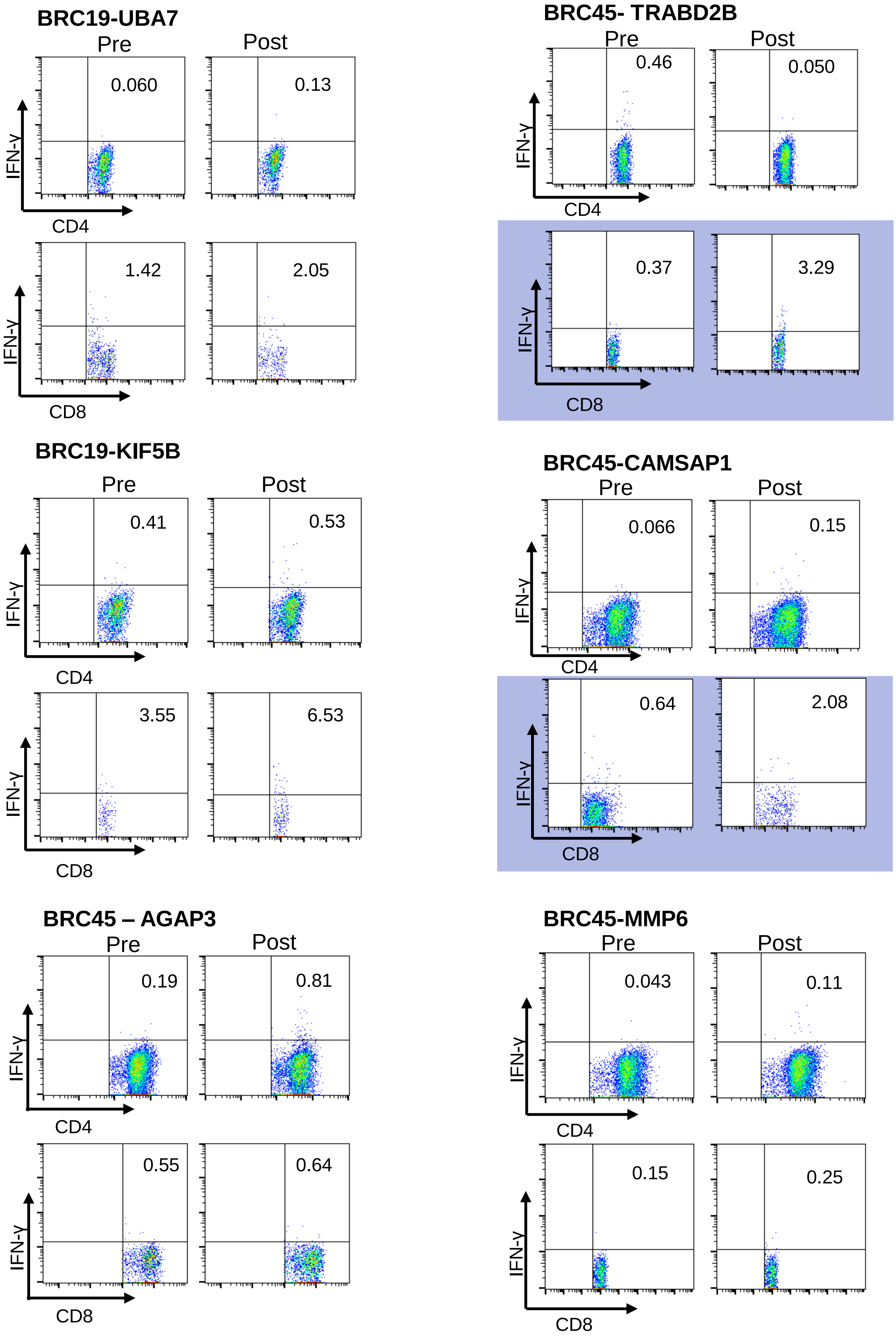

Supplementary figure 3-cont.

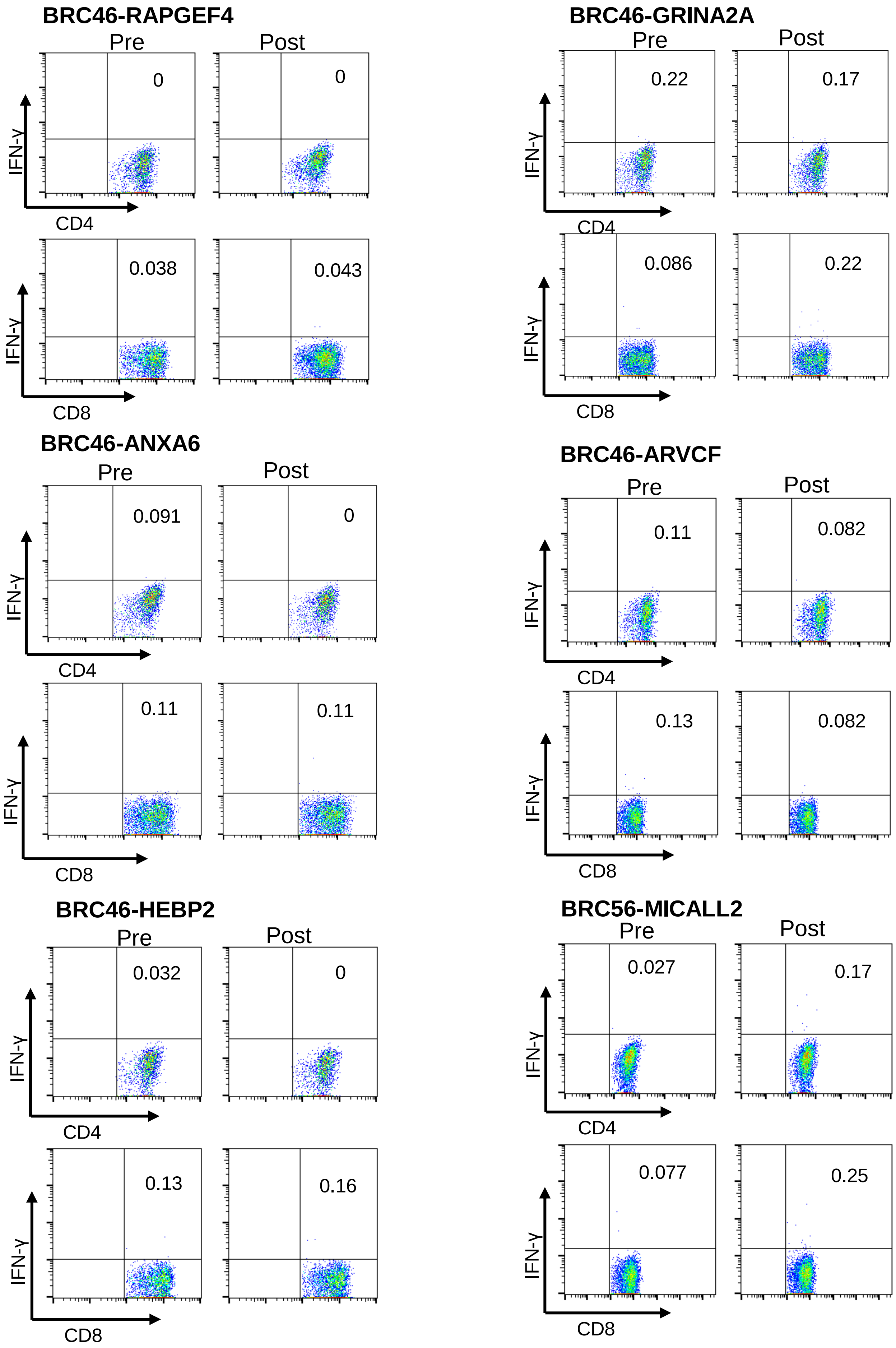

Supplementary figure 3-cont.

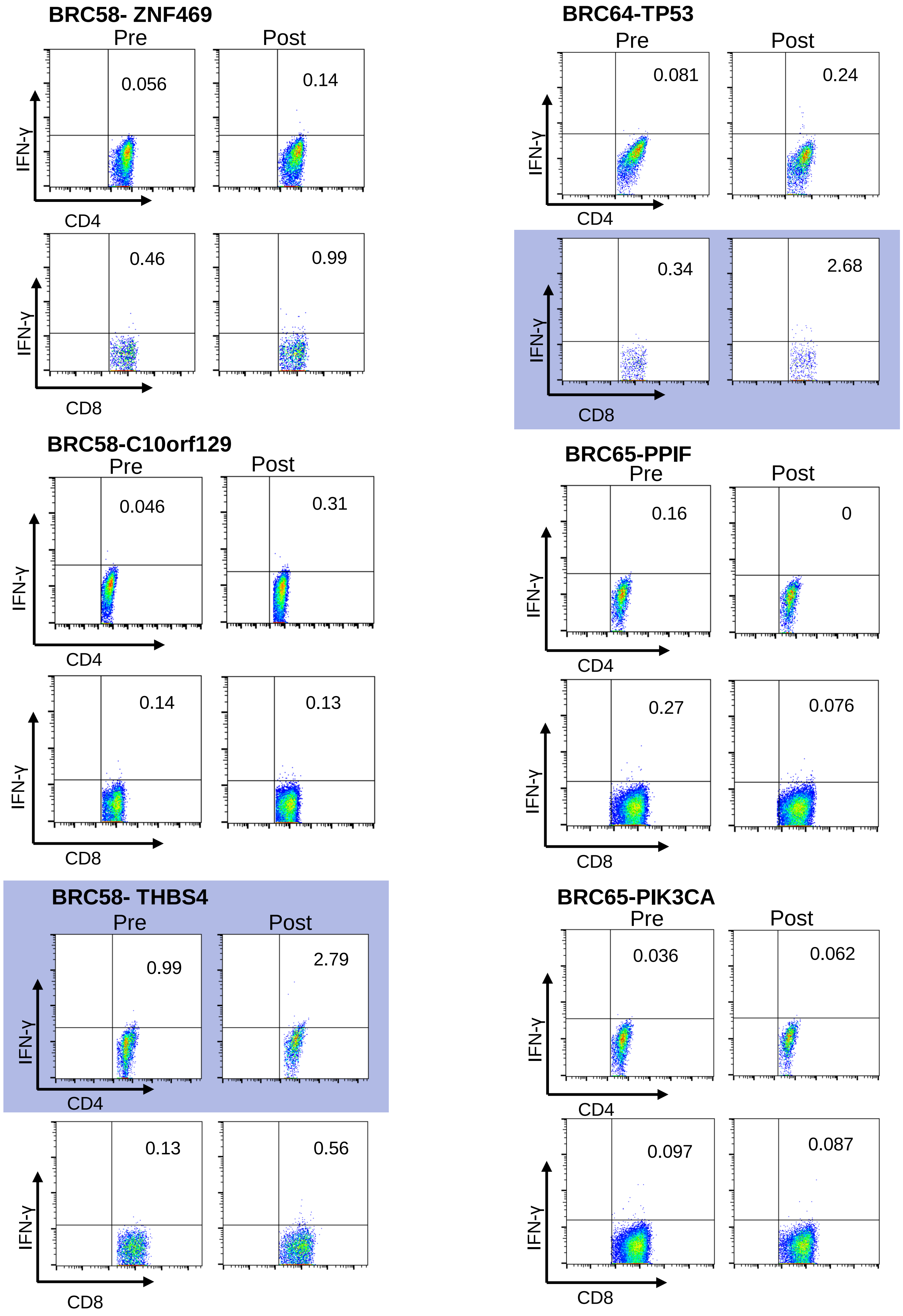

Supplementary figure 3-cont.

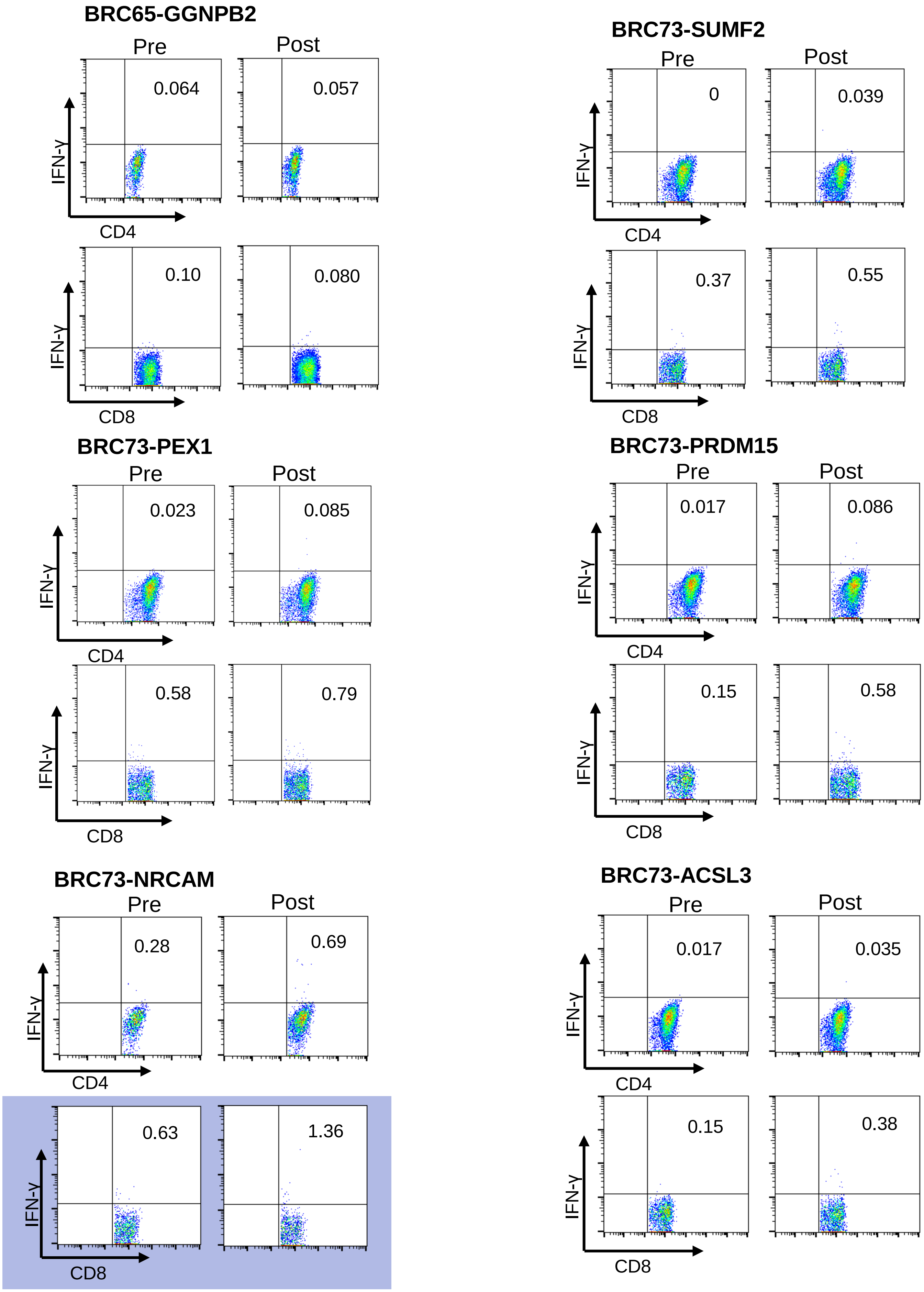

Supplementary figure 3-cont.

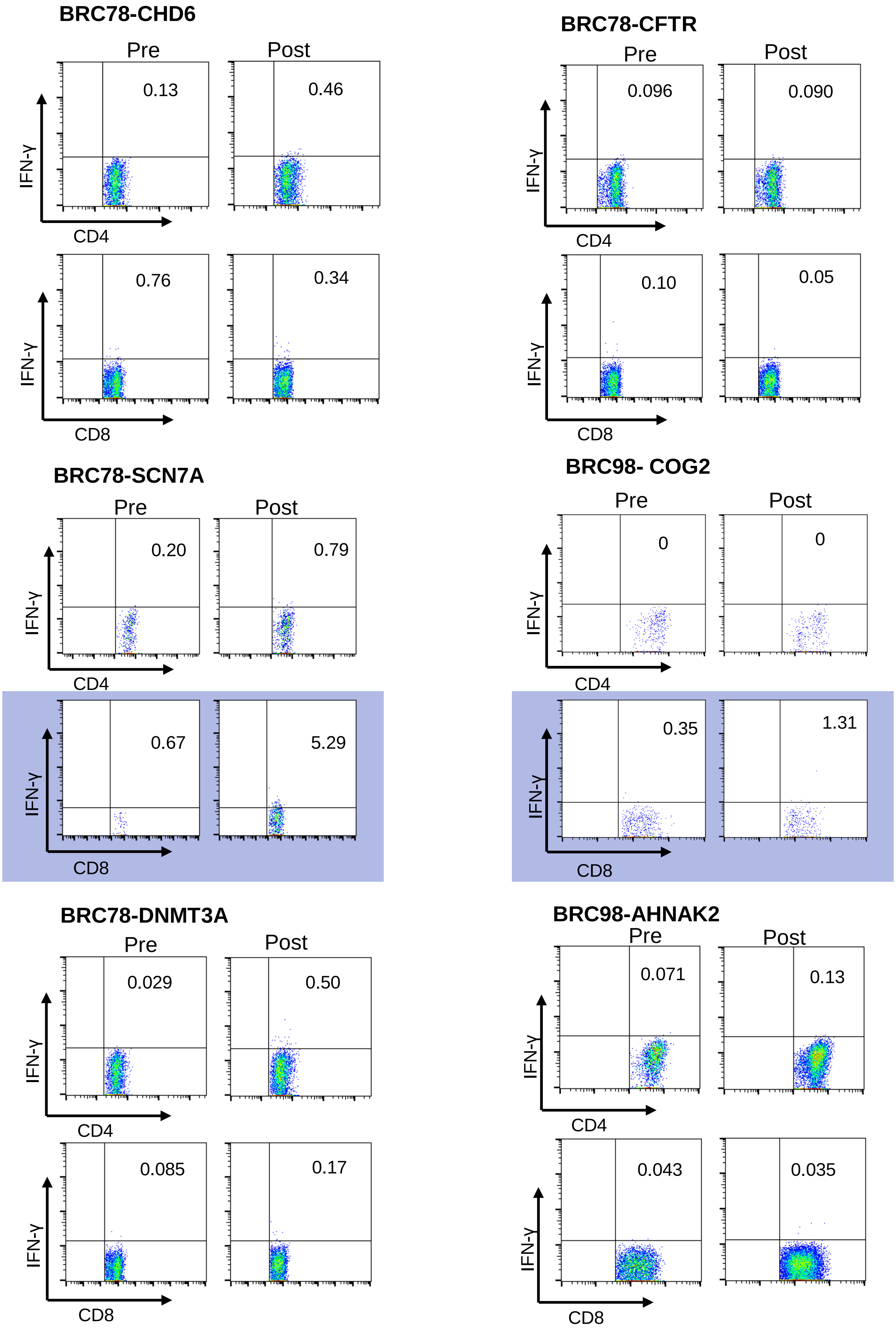

Fig. S3. Neoantigen vaccines elicit both CD4 and CD8 T cell responses. ICS results of all confirmed neoantigens outside of PIGM, ASB18, and MIB2. T cells from *in vitro* cultures as described in Figure 4 were analyzed by ICS for intracellular IFN-γ production after neoantigen peptide stimulation. Data represent percentage increase from pre- to post-vaccination. Increases between pre- and post-vaccination are considered positive when a two-fold or greater increase is observed in percent positive cells, with a minimum percent positive of $\geq$1% in the post-vaccination sample. All positive neoantigen responses are indicated by blue shading.

Fig. S4

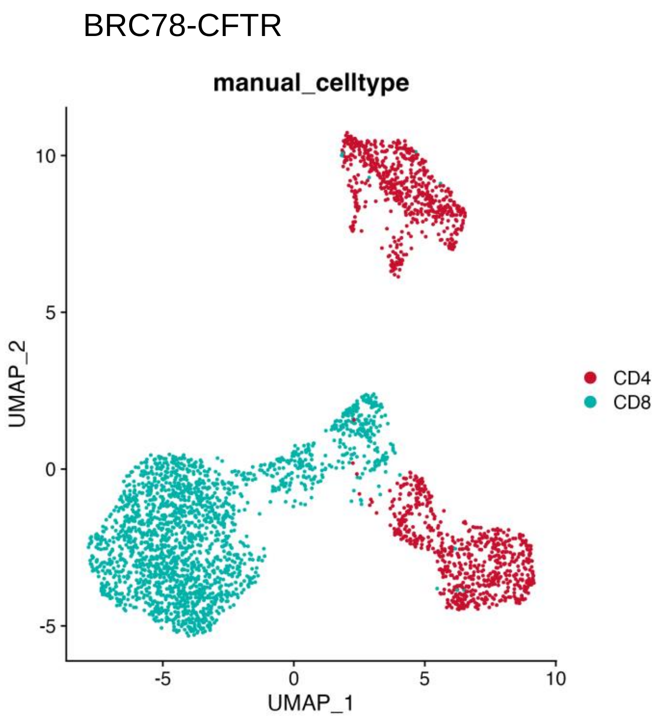

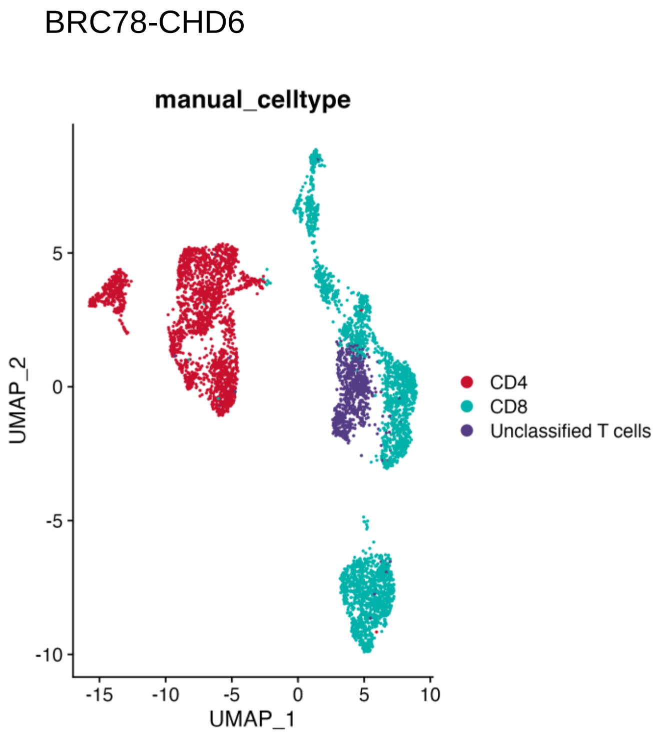

BRC78: CFTR antigen reactive T cell sequence analysis

TRAV	TRBV	TRBV CDR3 aa sequence	Frequency (%) in scTCR seq
V38J43C	V27DJ2-1C2	**CASSLYRGSYNEQFF**	41.1
V13-1J3	V19DJ2-1C1	CASSFNGYSGVEQFF	11.2
V38-2/DV8J43	V7-9-1DJ1-2C1	CASSLTDRTAVEGYTF	5.3
V29/DV5J31C	V5-6DJ2-2C2	CASSWGLDWEVTGELFF	4.1
V12-2J6C	V7-6DJ2-2C2	CASSLAWPGTRGTGELFF	3.2
V10J36C	V3-1DJ1-2C1	CASSQDFGADGYTF	3
V5J39C	V3-1DJ1-2C1	CASSQDFGADGYTF	2.5

Bold in red: Listed in the figure 5.

BRC78:CHD6 antigen reactive T cell sequence analysis

TRAV	TRBV	TRBV CDR3 aa sequence	Frequency (%) in scTCR seq
V12-2J39C	V6-5DJ1-3C1	**CASSQGQTGNTIYF**	21.6
V10J44C	V6-5DJ1-3C1	**CASSQGQTGNTIYF**	21.5
V38-1J26C	V29-1DJ1-1C1	CSVESGLGTEAFF	11.5
V8-6J58C	V5-4DJ1-3C1	**CASSLWGARNTIYF**	10.1
V38-2J31C	V14DJ2-1C2	CASSEYGSSYNEQFF	8.6
V8-4J54C	V7-2DJ2-6C2	**CASSLGPGAGANVLTF**	3.6
V12-3J45C	V20-1DJ2-7C2	CASGTGSEKLFF	2

Bold in black: shared CDR3 sequence in bulk TCRseq ans scTCRseq

Bold in red: Listed in the figure 5.

Fig. S4. scTCR-seq analysis shows a oligoclonal expansion of TCRs. PBMC from patient BRC78, collected two weeks post vaccination was cultured for 22 days in the presence of either CFTR or CHD6 neoantigen peptide. Cells were tagged with antibodies to CD4 and CD8, and subjected to scTCR-seq. (A) UMAP plots show that both CD4 and CD8 T cells expanded in culture. (B) Assessment of the clonotypic phenotype showed outgrowth of selected TCRs that matched data from bulk TCR-seq, as shown in Figure 5.

Fig. S5

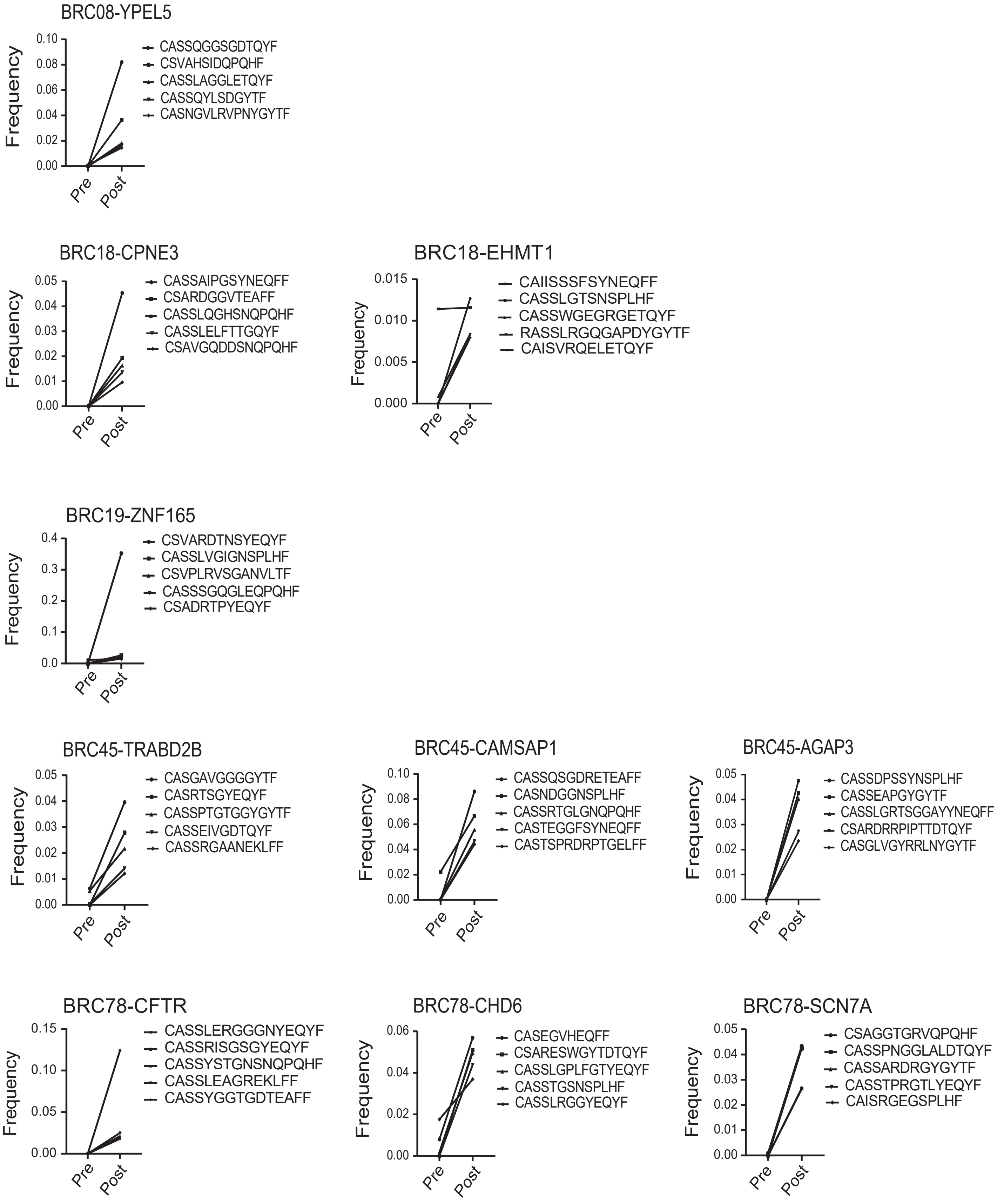

BRC08-YPEL5
Frequency
0.10
0.08
0.06
0.04
0.02
0.00
Pre
Post
CASSQGGSGDTQYF
CSVAHSIDQPQHF
CASSLAGGLETQYF
CASSQYLSDGYTF
CASNGVLRVPNYGYTF
BRC18-CPNE3
Frequency
0.05
0.04
0.03
0.02
0.01
0.00
Pre
Post
CASSAIPGSYNEQFF
CSARDGGVTEAFF
CASSLQGHSNQPQHF
CASSLELFTTGQYF
CSAVGQDDSNQPQHF
BRC18-EHMT1
Frequency
0.015
0.010
0.005
0.000
Pre
Post
CAIISSSFSYNEQFF
CASSLGTSNSPLHF
CASSWGEGRGETQYF
RASSLRGQGAPDYGYTF
CAISVRQELETQYF
BRC19-ZNF165
Frequency
0.4
0.3
0.2
0.1
0.0
Pre
Post
CSVARDTNSYEQYF
CASSLVGIGNSPLHF
CSVPLRVSGANVLTF
CASSSGQGLEQPQHF
CSADRTPYEQYF
BRC45-TRABD2B
Frequency
0.05
0.04
0.03
0.02
0.01
0.00
Pre
Post
CASGAVGGGGYTF
CASRTSGYEQYF
CASSPTGTGGYGYTF
CASSEIVGDTQYF
CASSRGAANEKLFF
BRC45-CAMSAP1
Frequency
0.10
0.08
0.06
0.04
0.02
0.00
Pre
Post
CASSQSGDRETEAFF
CASNDGGNSPLHF
CASSRTGLGNQPQHF
CASTEGGFSYNEQFF
CASTSPRDRPTGELFF
BRC45-AGAP3
Frequency
0.05
0.04
0.03
0.02
0.01
0.00
Pre
Post
CASSDPSSYNSPLHF
CASSEAPGYGYTF
CASSLGRTSGGAYYNEQFF
CSARDRRPIPTTDTQYF
CASGLVGYRRLNYGYTF
BRC78-CFTR
Frequency
0.15
0.10
0.05
0.00
Pre
Post
CASSLERGGGNYEQYF
CASSRISGSGYEQYF
CASSYSTGNSNQPQHF
CASSLEAGREKLFF
CASSYGGTGDTEAFF
BRC78-CHD6
Frequency
0.06
0.04
0.02
0.00
Pre
Post
CASEGVHEQFF
CSARESWGYTDTQYF
CASSLGPLFGTYEQYF
CASSTGSNSPLHF
CASSLRGGYEQYF
BRC78-SCN7A
Frequency
0.05
0.04
0.03
0.02
0.01
0.00
Pre
Post
CSAGGTGRVQPQHF
CASSPNGGLALDTQYF
CASSARDRGYGYTF
CASSTPRGTLYEQYF
CAISRGEGSPLHF

Fig S5. Vaccination promotes expansion of select T cell clones. Summary of TCRVβ clonotypes identified, using TCRVβ CDR3 reference libraries in T cells. PBMC obtained before and after vaccination were stimulated with neoantigen peptide for 12 days. Expanded T cells were subjected to sequencing for TCRVβ CDR3 regions for patients BRC78 (CFTR, SCN7A, CHD6), BRC18 (CPNE3 and EHMT1), BRC19 (ZNF165), BRC45 (TRABD2B, AGAP3, CAMSAP1), and BRC08 (YPEL5). Each symbol represents a unique CDR3 sequence and the frequencies of the top 5 CDR3 sequences detected in post vaccination samples are listed in each panel, as well as their matching frequencies in pre-vaccination samples.
