# Supplementary material for: Neoantigen DNA vaccines are safe, feasible, and induce neoantigen-specific immune responses in triple-negative breast cancer patients
Source: Genome Med. 2024 Nov 14;16:131. doi: 10.1186/s13073-024-01388-3 (PMC11562513; doi:10.1186/s13073-024-01388-3)
Supplement: Supplementary file 3 — Additional file 3: clinical trial protocol (pdf): 20155073 protocol 05-22-19. [file 13073_2024_1388_MOESM3_ESM.pdf]

A PHASE 1 CLINICAL TRIAL TO EVALUATE THE SAFETY AND IMMUNOGENICITY OF A PERSONALIZED POLYEPITOPE DNA VACCINE STRATEGY IN BREAST CANCER PATIENTS WITH PERSISTENT TRIPLE-NEGATIVE DISEASE FOLLOWING NEOADJUVANT CHEMOTHERAPY

HRPO #: 201505074
Version date: 05/22/19
IND #: 16408
ClinicalTrials.gov #: NCT02348320

INVESTIGATORS

Principal Investigator:
William E. Gillanders, M.D.
Washington University School of Medicine
660 South Euclid, Box 8109
St. Louis, MO 63110
Telephone: 314-747-0072
E-mail: gillandersw@wustl.edu

Co-Investigators:
Elaine Mardis, Ph.D.
Genome Scientist
The Genome Center at WUSM
660 South Euclid, Box 8501
St. Louis, MO 63110
Telephone: 314-286-1805
E-mail: emardis@wustl.edu

Timothy Eberlein, M.D.
Breast Surgeon
Washington University School of Medicine
660 South Euclid, Box 8109
St. Louis, MO 63110
Telephone: 314-362-8020
E-mail: eberleint@wustl.edu

Feng Gao, M.D., Ph.D.
Biostatistician
Washington University School of Medicine
660 South Euclid, Box 8067
St. Louis, MO 63110
Telephone: 314-362-3682
E-mail: feng@wustl.edu

S. Peter Goedegebuure, Ph.D.
Immunologist
Washington University School of Medicine
660 South Euclid, Box 8109
St. Louis, MO 63110
Telephone: 314-362-8612
E-mail: goedegep@wustl.edu

Foluso O. Ademuyiwa, M.D., MPH
Medical Oncologist
Washington University School of Medicine
660 South Euclid, Box 8056
St. Louis, MO 63110
Telephone: 314-362-7201
E-mail: bisiademuyiwa@wustl.edu

Robert D. Schreiber, Ph.D.
Immunobiologist
Washington University School of Medicine
660 South Euclid, Box 8118
St. Louis, MO 63110
Telephone: 314-362-8747
E-mail: Schreiber@wustl.edu

SUMMARY

**Protocol:**	**A PHASE 1 CLINICAL TRIAL TO EVALUATE THE SAFETY AND IMMUNOGENICITY OF A PERSONALIZED POLYEPITOPE DNA VACCINE STRATEGY IN BREAST CANCER PATIENTS WITH PERSISTENT TRIPLE-NEGATIVE DISEASE FOLLOWING NEOADJUVANT CHEMOTHERAPY**
**Study Design:**	This is a phase 1 open-label study to evaluate the safety and immunogenicity of a personalized polyepitope DNA vaccine strategy. The personalized polyepitope DNA vaccines will be formulated as naked plasmid DNA vaccines. The hypothesis of this study is that personalized polyepitope DNA vaccines will be safe for human administration and capable of generating measurable CD8 T cell responses to mutant tumor-specific antigens. The primary objective of this study is to demonstrate the safety of the personalized polyepitope DNA vaccine strategy. The secondary objective is to evaluate the immunogenicity of the personalized polyepitope DNA vaccine strategy as measured by ELISPOT analysis and multi-parametric flow cytometry, surrogates for CD8 T cell function.
**Product Description:**	The personalized polyepitope DNA vaccines are composed of closed circular DNA plasmids that are designed to express mutant tumor specific antigens identified by genome sequencing. These plasmids will be formulated as naked DNA plasmid vaccines. Vaccine vials will be supplied at a concentration of 2 mg/mL. DNA vaccines will be administered intramuscularly (in the deltoid or lateralis) using a TriGrid electroporation device (Ichor Medical Systems). Two injections (2 mg/injection, total dose 4 mg) will be administered at each time point.
**Subjects:**	Breast cancer patients must have persistent triple-negative breast cancer following neoadjuvant chemotherapy without evidence of metastatic disease. Patients enrolled into the protocol must provide consent for genome sequencing and dbGAP-based data sharing and provide germline and tumor DNA samples of adequate quality for sequencing. Fresh tissue is preferred but archival tissue is allowed.
**Study Plan:**	Thirty breast cancer patients will be enrolled. Subjects will be treated by electroporation with 4 mg of a personalized polyepitope DNA vaccine at Day 1, Day 29 ± 7, and Day 57 ± 7 with at least 21 days between injection days. Each DNA vaccination will be 4 mg vaccine administered intramuscularly using a TriGrid electroporation device.
**Study Duration:**	Each subject will be followed for 12 months following the last vaccination. Additional follow-up visits or telephone contact will be scheduled annually thereafter if the patient is alive and available for follow-up.
**Study Endpoints:**	The primary endpoint is safety of the vaccine regimen. Safety will be closely monitored after injection with eight or more clinical and laboratory assessments in the first six months of the trial. Toxicity will be graded according to the National Cancer Institute Common Terminology Criteria for Adverse Events version 4.0. The secondary endpoint is immunogenicity of the vaccine regimen as measured by ELISPOT analyses and multiparametric flow cytometry. Exploratory analyses will include other measures of immune function.

PROTOCOL REVISION HISTORY

Initial Approval	05/18/15
Amendment #1	07/08/15
Amendment #2	11/17/15
Amendment #3	09/26/16
Amendment #4	10/25/16
Amendment #5	12/16/16
Amendment #6	03/07/17
Amendment #7 (QASMC)	06/22/17
Amendment #7	07/14/17
Amendment #8	03/16/18
Amendment #9	05/22/19

PROTOCOL SIGNATURE PAGE

A PHASE 1 CLINICAL TRIAL TO EVALUATE THE SAFETY AND IMMUNOGENICITY OF A PERSONALIZED POLYEPITOPE DNA VACCINE STRATEGY IN BREAST CANCER PATIENTS WITH PERSISTENT TRIPLE-NEGATIVE DISEASE FOLLOWING NEOADJUVANT CHEMOTHERAPY

I have read the attached clinical protocol and agree to conduct this trial in accordance with all the stipulations of the protocol and in accordance with the Declaration of Helsinki/Tokyo/Venice on Experimentation in Humans as required by the United States Food and Drug Administration regulations, Code of Federal Regulations Title 21 parts 50, 56, 312, 800, Title 45 part 46 and all applicable guidelines.

Name of Investigator:

____

Signature   Date

TABLE OF CONTENTS

SUMMARY 2

PROTOCOL REVISION HISTORY 3

PROTOCOL SIGNATURE PAGE 4

1 OBJECTIVES 8
- 1.1 PRIMARY OBJECTIVE 8
- 1.2 SECONDARY OBJECTIVE 8
- 1.3 EXPLORATORY OBJECTIVES 8

2 BACKGROUND 9
- 2.1 MUTANT TUMOR-SPECIFIC ANTIGENS 9
- 2.2 NEXT GENERATION SEQUENCING AND CANCER VACCINES 9
- 2.3 SEQUENCING STRATEGIES TO IDENTIFY MUTANT TUMOR-SPECIFIC ANTIGENS 10
- 2.4 PRIORITIZATION OF SEQUENCING-IDENTIFIED MUTANT TUMOR-SPECIFIC ANTIGENS 11
- 2.5 POLYEPITOPE DNA VACCINES 11
- 2.6 ELECTROPORATION 12
- 2.7 RATIONALE FOR TESTING IN ADJUVANT SETTING 12

3 PATIENT SELECTION 14
- 3.1 INCLUSION CRITERIA 14
- 3.2 EXCLUSION CRITERIA 14
- 3.3 INCLUSION OF WOMEN AND MINORITIES 15

4 REGISTRATION PROCEDURES 16
- 4.1 PRIOR TO REGISTRATION 16
- 4.2 REGISTRATION PROCEDURES 16
- 4.3 CONFIRMATION OF PATIENT ELIGIBILITY 16
- 4.4 PATIENT REGISTRATION IN THE SITEMAN CANCER CENTER DATABASE 17
- 4.5 ASSIGNMENT OF UPN 17

5 INVESTIGATIONAL AGENT 18
- 5.1 CHEMICAL NAME AND STRUCTURE 18
- 5.2 MANUFACTURING FACILITY 20
- 5.3 MANUFACTURING PROCESS 22
- 5.4 PRODUCT FORMULATION 25
- 5.5 PRODUCT RELEASE TESTS 26
- 5.6 STABILITY 27
- 5.7 LABELS 27
- 5.8 SITEMAN CANCER CENTER INVESTIGATIONAL PHARMACY 27
- 5.9 INVESTIGATIONAL AGENT ADMINISTRATION 28
- 5.10 INVESTIGATIONAL AGENT ACCOUNTABILITY 32

6 NONCLINICAL STUDIES 33
- 6.1 OVERVIEW 33
- 6.2 PRECLINICAL STUDIES 33
- 6.3 RATIONALE FOR NO GLP SAFETY STUDIES 35
- 6.4 RATIONALE FOR NO GLP BIODISTRIBUTION STUDIES 36

7 TREATMENT PLAN 37
- 7.1 PLASMID DNA ADMINISTRATION 37
- 7.2 DURATION OF THERAPY 37
- 7.3 DURATION OF FOLLOW UP 37

8 POTENTIAL TOXICITY AND DOSE MODIFICATIONS 38

8.1 PREVIOUS HUMAN EXPERIENCE 38
8.2 POTENTIAL TOXICITY 39
8.3 TOXICITY MONITORING AND MANAGEMENT 40
8.4 DOSE MODIFICATIONS 40

9 STUDY CALENDAR 41

10 CRITERIA FOR RESPONSE 42

10.1 PRIMARY OBJECTIVE: SAFETY 42
10.2 SECONDARY OBJECTIVES: EVALUATION OF THE IMMUNE RESPONSE 42
10.3 EXPLORATORY OBJECTIVES 43

11 REMOVAL OF SUBJECTS FROM PROTOCOL THERAPY 45

11.1 REMOVAL OF SUBJECTS FROM PROTOCOL THERAPY 45
11.2 VOLUNTARY SUBJECT WITHDRAWAL 45
11.3 PROCEDURE FOR DISCONTINUATION 45

12 ADVERSE EVENT REPORTING 46

12.1 DEFINITIONS 46
12.2 REPORTING TO THE HUMAN RESEARCH PROTECTION OFFICE (HRPO) AT WUSM 47
12.3 REPORTING TO THE QUALITY ASSURANCE AND SAFETY MONITORING COMMITTEE (QASMC) AT WASHINGTON UNIVERSITY 48
12.4 REPORTING TO THE INSTITUTIONAL BIOSAFETY COMMITTEE 48
12.5 REPORTING TO THE FDA 48
12.6 REPORTING TO THE NIH OFFICE OF BIOTECHNOLOGY ACTIVITIES (OBA) 48
12.7 REPORTING TO ICHOR MEDICAL SYSTEMS 49
12.8 TIMEFRAME FOR REPORTING REQUIRED EVENTS 49

13 DATA AND SAFETY MONITORING 50

13.1 PROTOCOL STOPPING CRITERIA 50
13.2 DATA SAFETY MONITORING PLAN 50
13.3 DEVELOPMENTAL THERAPEUTICS 51
13.4 NIH RECOMBINANT DNA ADVISORY COMMITTEE 51

14 STATISTICAL CONSIDERATIONS 53

14.1 STUDY DESIGN/OBJECTIVES 53
14.2 SAMPLE SIZE AND ACCRUAL 53
14.3 DATA ANALYSIS 55

15 DATA MANAGEMENT 56

16 REGULATORY AND ETHICAL OBLIGATIONS 58

16.1 INFORMED CONSENT 58
16.2 INSTITUTIONAL REVIEW BOARD 58
16.3 SUBJECT CONFIDENTIALITY 58

17 ADMINISTRATIVE AND LEGAL OBLIGATIONS 59

17.1 STUDY DOCUMENTATION AND RETENTION OF RECORDS 59
17.2 POLICY REGARDING RESEARCH-RELATED INJURIES 59
17.3 STUDY TERMINATION 59

18 REFERENCES 60

19 APPENDICES 64

19.1 ABBREVIATIONS 64

19.2 ECOG/ZUBROD PERFORMANCE STATUS SCALE ____ 65
19.3 NATIONAL CANCER INSTITUTE COMMON TERMINOLOGY CRITERIA FOR ADVERSE EVENTS ____ 65

1 OBJECTIVES

This is a phase 1 open-label study to evaluate the safety and immunogenicity of a personalized polyepitope DNA vaccine strategy. The personalized polyepitope DNA vaccine strategy is designed to target prioritized mutant tumor-specific antigens that are present in an individual patient's breast cancer but not in corresponding normal tissues. The vaccines will be formulated as naked plasmid DNA vaccines, and will be administered at a dose of 4 mg intramuscularly using an integrated electroporation administration system (TDS-IM system, Ichor Medical Systems). The hypothesis of this study is that the personalized polyepitope DNA vaccine strategy will be safe for human administration and capable of generating measurable CD8 T cell responses to the mutant tumor-specific antigens.

1.1 Primary objective

The primary objective is to evaluate the safety of the personalized polyepitope DNA vaccine strategy.

1.2 Secondary objective

The secondary objective is to evaluate the immunogenicity of the personalized polyepitope DNA vaccine strategy. Immunogenicity will be measured by ELISPOT analysis and multiparametric flow cytometry, surrogates for CD8 T cell function,

1.3 Exploratory objectives

The exploratory objectives are to evaluate the immunogenicity, and clinical response that may be associated with immune responses induced by the personalized polyepitope DNA vaccine strategy.

Additional assays of T cell immunity will include cytotoxicity assays. Clinical responses and time to disease progression will be evaluated with physical examination and diagnostic imaging as clinically indicated.

2 BACKGROUND

2.1 Mutant tumor-specific antigens

The hypothesis of the proposed phase 1 clinical trial is that a personalized polyepitope DNA vaccine strategy targeting mutant tumor-specific antigens is safe for human use, and can generate CD8 T cell responses to the targeted mutant tumor-specific antigens. Although not specifically addressed in the trial, the underlying hypothesis is that personalized cancer vaccine strategies are superior to cancer vaccine strategies targeting shared tumor antigens.

Tumor antigens are often classified as shared tumor antigens and tumor-specific antigens. The majority of tumor-specific antigens are now believed to be the result of somatic mutations present in the tumor.

Shared tumor antigens are expressed in multiple cancers, and are often self-differentiation antigens that are expressed in a limited subset of normal tissues, but overexpressed in cancers. Examples of shared tumor antigens include MAGE (melanoma) [1], prostatic acid phosphatase (prostate cancer) [2], and HER2/neu (breast cancer) [3].

Tumor-specific antigens are uniquely expressed in individual cancers, and are typically the result of point mutations or other genetic changes that are present only in the tumor (reviewed in [4, 5]). As such, tumor-specific antigens represent the only antigens that are truly unique to the tumor and not expressed in normal tissues. The first human mutant tumor-specific antigen was described in 1995, resulting from a point mutation of cyclin-dependent kinase (CDK4) [6]. Since that time additional publications have described the expression of mutant tumor-specific antigens in melanoma [7], non-small cell lung cancer [8] and other human cancers [9].

Cancer vaccine strategies targeting mutant tumor-specific antigens have clear conceptual advantages over strategies targeting shared tumor antigens. Conceptual advantages include: (1) Targeting mutant tumor-specific antigens is potentially safer. Mutant tumor-specific antigens are expressed only in the tumor, decreasing the risk of autoimmunity. (2) Targeting mutant tumor-specific antigens is potentially more effective. T cell responses to mutant tumor-specific antigens are high in affinity, and are not limited by central mechanisms of self-tolerance. (3) Targeting mutant tumor-specific antigens potentially limits antigen-loss, a common tumor escape mechanism. One of the hallmarks of cancer is genome instability, and one clear weakness of cancer vaccines that target a single shared tumor antigen is antigen-loss. Targeting multiple mutant tumor-specific antigens may preclude antigen loss. In addition, many mutant tumor-specific antigens play a functional role in neoplastic transformation (driver mutations). Immune selection resulting in loss of driver mutations may fundamentally alter the phenotype of targeted cancers. (4) Targeting mutant tumor-specific antigens is likely to be universally applicable in solid tumors. Solid tumors appear to have a remarkable number of nonsynonymous mutations present (each nonsynonymous mutation is a candidate mutant tumor-specific antigen), suggesting that a personalized vaccine approach could be used in most solid tumor patients, regardless of intrinsic subtype or HLA type.

2.2 Next generation sequencing and cancer vaccines

Cancer genome sequencing is a major focus area for Siteman Cancer Center, and for the Genome Institute at Washington University School of Medicine (WUSM). We recently used next-generation sequencing technologies to sequence and compare four DNA samples (primary tumor, brain metastasis, xenograft of the primary tumor, and peripheral blood) from an African-American patient with basal-like breast cancer. Of note, next-generation sequencing technologies are particularly well suited to breast cancer genome sequencing. Breast cancer is a heterogenous disease, but genome sequencing at almost 40× haploid coverage allowed us to precisely calculate mutant allele frequencies, demonstrating genome remodeling, and unexpected similarities between the brain metastasis and xenograft [10].

Additional studies are currently ongoing, and we have successfully sequenced over 47 breast cancer tumor/normal genomes in postmenopausal breast cancer patients with luminal disease enrolled in the American College of Surgeons Oncology Group Z1031 clinical trial (neoadjuvant endocrine therapy) [11]. Although the main objective of this study was to define a genetic profile of susceptibility and/or resistance

to aromatase inhibitor therapy, the results do provide important insights into the rational development of vaccine strategies for targeting mutant tumor-specific antigens.

In the 47 luminal breast cancer genomes sequenced, we identified and validated 1415 nonsynonymous single-nucleotide variant (SNV) mutations. This means that approximately 31 nonsynonymous SNV mutations are present per luminal breast cancer genome, similar to the number identified in our previous studies of a basal-like breast cancer [10], and similar to the number predicted by limited exome sequencing [12], and statistical models [13]. 79 genes contained nonsynonymous SNV mutations in more than one breast cancer (recurrently mutated genes). The most common recurrently mutated genes include *PIK3CA* (mutations present in 21 breast cancers), *MT-ND5* (11 breast cancers), *TP53* (7 breast cancers), *SYNE1* (6 breast cancers), and *TTN* (5 breast cancers). Of the remaining 79 recurrently mutated genes, nonsynonymous SNV mutations were present in < 10% of the breast cancers studied. Of note, we identified 10 different *PIK3CA* mutations, and the most common mutation, p.H1047R, was present in 10 cancers. Our interpretation of these data is that a personalized vaccine approach is significantly more attractive than an off-the-shelf vaccine approach targeting recurrent mutations. Given the diversity of mutations observed, the limited number of recurrent mutations present in >10% of patients, and the fact that off-the-shelf vaccines would be restricted by HLA type, we estimate that < 10% of breast cancer patients would be eligible for an off-the-shelf vaccine targeting the most common *PIK3CA* mutation. Hence, even if multiple off-the-shelf vaccines were available for different recurrent mutations, only a limited number of patients would be eligible. However, given the number of mutations consistently observed in breast cancer, it is likely that at least a subset of these mutations could be successfully targeted by a personalized vaccine approach. These results suggest that a personalized vaccine approach is the best strategy to target mutant tumor-specific antigens.

There are two conceptual strategies for creating personalized cancer vaccines targeting mutant tumor-specific antigens: a candidate epitope strategy, and an unbiased strategy. The candidate epitope strategy uses computer algorithms [14, 15] and *in vitro* studies to predict immunodominant epitopes, which are then integrated into a personalized vaccine. In the unbiased strategy, no attempt is made to identify the immunodominant epitopes, and all candidate mutant tumor-specific antigens are integrated into a personalized vaccine.

We have considered both the candidate epitope strategy and the unbiased strategy. We believe that the candidate epitope strategy is superior to the unbiased strategy for the following reasons. (1) Preliminary data from preclinical models and human correlative studies suggest that relatively few sequencing-identified mutant tumor-specific antigens are processed, presented and effectively recognized by the immune system. (2) We have now developed and validated algorithms for the prediction and prioritization of sequencing-identified mutant tumor-specific antigens [16, 17]. (3) Targeting a limited number of prioritized sequencing-identified mutant tumor-specific antigens will facilitate vaccine design and manufacture, and streamline immune monitoring.

2.3 Sequencing strategies to identify mutant tumor-specific antigens

Robust next-generation sequencing strategies for the identification of mutant tumor-specific antigens will be required for the successful clinical translation of personalized cancer vaccine strategies. As such, a major focus of our research studies has been the development of cost-effective and accurate next-generation sequencing strategies to identify mutant tumor-specific antigens and validate the expression of these antigens at the mRNA level. Initially a cancer genome sequencing approach was used. While cancer whole genome sequencing is informative and provides comprehensive information about both the coding and noncoding regions of the genome, this level of information may not be necessary for identifying mutant tumor-specific antigens, or prioritizing antigens for immune intervention. We have now confirmed that tumor/normal exome sequencing is a robust and accurate strategy for the identification of mutant tumor-specific antigens [17]. Of note, recent studies suggest that approximately 40% of mutations identified by cancer exome sequencing are not expressed at the mRNA level, so it is important to confirm expression of the mutant allele at the mRNA level. To evaluate mRNA expression, we have performed cDNA-capture sequencing analyses. We have confirmed that cDNA-capture sequencing can be used to successfully confirm expression of sequencing-identified mutant tumor-specific antigens at the mRNA level. This analysis also provides an estimation of how highly expressed the mutated allele is expressed relative to other genes in the tumor. For the phase 1 clinical trial proposed, tumor/normal exome

sequencing analysis will be used to identify mutations (single nucleotide variants, insertions and deletions) present only in the tumor, and cDNA-capture sequencing will be used to confirm mutant allele expression and expression level in the tumor mRNA.

2.4 Prioritization of sequencing-identified mutant tumor-specific antigens

Of note, we have now developed and validated an epitope prediction algorithm for the prioritization of sequencing-identified mutant tumor-specific antigens. Once somatic mutations have been identified and mutant mRNA expression confirmed/quantified using the sequencing strategies outlined above, mutant tumor-specific antigens will be prioritized using an epitope prediction algorithm that has been designed to select and prioritize the most promising sequencing-identified mutant tumor-specific antigens. Currently, the most commonly used CD8 T cell epitope prediction algorithm is NetMHC. However, collaborative work conducted by Robert Schreiber, Elaine Mardis, Max Artyomov and William Gillanders has shown that a much more accurate prediction comes from calculating a median affinity for each sequencing-predicted mutant epitope using multiple epitope prediction algorithms (NetMHC Pan; ANN; SMM and others). We have significantly improved this epitope prediction algorithm by applying three filters to the initial prioritized output list: (a) elimination of hypothetical proteins; (b) use of an antigen processing algorithm to eliminate epitopes that are not likely to be proteolytically produced by constitutive proteasomes or immunoproteasomes; and (c) prioritization of “neo-epitopes” identified by a higher affinity binding of the mutant peptide sequence compared to the wildtype peptide sequence. The final output of these analyses is a rank-ordered list of the highest to lowest priority sequencing-identified mutant tumor-specific antigens for each individual patient. In experiments performed using preclinical mouse sarcoma models, this refined prediction algorithm has successfully identified the major tumor rejection antigens in three out of three tumors tested to date [16, 17]. To our knowledge, this is the only algorithm that has been successfully applied to date to cancer vaccine development. Additional information about the preclinical validation of the epitope prediction algorithm is provided in Section 10 NONCLINICAL STUDIES. A similar process will be used to identify and prioritize HLA class II neoantigens.

Once a rank-ordered list of the highest to lowest priority sequencing-identified mutant tumor-specific peptide antigens is generated, we may perform *in vitro* binding studies to confirm that the prioritized sequencing-identified mutant tumor-specific peptide antigens can bind to and stabilize the appropriate HLA class I allele. Up to 20of the highest priority sequencing-identified mutant tumor-specific antigens that are confirmed to bind and stabilize HLA class I or class II molecules will be targeted using the personalized DNA vaccine strategy detailed below.

2.5 Polyepitope DNA vaccines

The personalized polyepitope DNA vaccine strategy is based on the DNA vaccine platform. The observation that direct administration of recombinant DNA can generate potent immune responses established the field of DNA vaccines in the early 1990s [18-23]. Since that time, DNA vaccines have remained an area of intense research interest, and vaccines targeting infectious disease agents and cancers have progressed into clinical trials. Advantages of the DNA vaccine platform include the remarkable safety profile of DNA vaccines, and the relative ease of manufacture relative to proteins and other biologics. Perhaps most important, however, is the molecular flexibility of the DNA vaccine platform, with the ability to genetically manipulate encoded antigens, and/or incorporate other genes to amplify the immune response [24, 25]. The molecular flexibility of the DNA vaccine platform allows us to target multiple mutant tumor-specific antigens using a single polyepitope DNA vaccine. Polyepitope DNA vaccines integrate multiple epitopes in a single construct (Figure 1). We have optimized the polyepitope DNA vaccine platform to maximize antigen presentation of mutant tumor-specific antigens by integrating a mutant ubiquitin molecule. Because MHC class I binding peptides are initially processed in the cytosol by the ubiquitin/proteasome pathway, we have integrated a mutant form of ubiquitin (UbG76V). Fusion of UbG76V to the N-terminus of the polyepitope construct promotes epitope generation and display.

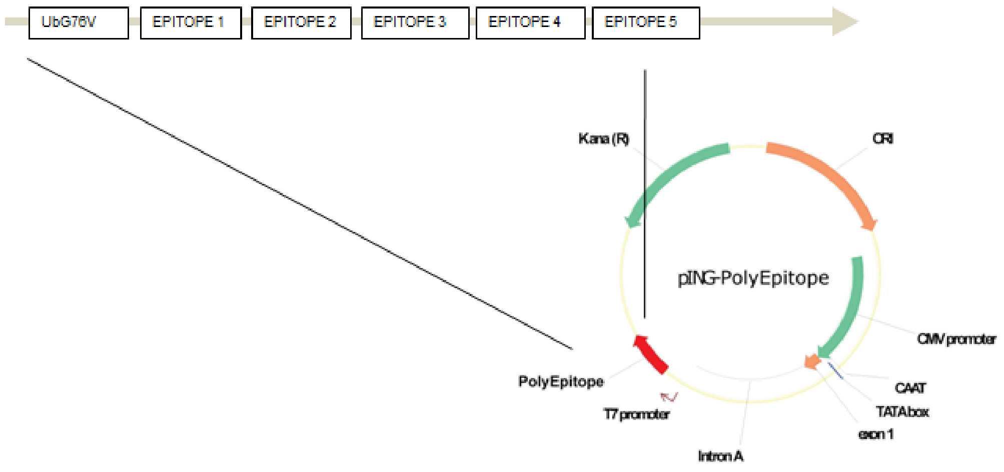

Figure 1: Polyepitope DNA vaccine design. Personalized polyepitope DNA vaccines integrate multiple epitopes in a single construct. We have optimized the polyepitope DNA vaccine platform to maximize antigen presentation of the sequencing-identified mutant tumor-specific antigens by integrating a mutant ubiquitin molecule.

2.6 Electroporation

Recent research provides valuable insights into why preclinical studies of DNA vaccines have been successful in rodents, but less successful in larger animals. One difficulty is scaling up DNA vaccine dose and injection volume [26]. In rodents, hydrostatic pressure from injecting DNA in a relatively large liquid volume significantly improves cellular uptake of DNA and antigen expression [27]. This effect is reduced in larger animals as the relative volume injected and hydrostatic pressure is reduced. Electroporation dramatically increases DNA uptake by muscle cells, antigen expression, and immunogenicity [28-31]. Of particular note, electroporation has now been used successfully in non-human primates, with responses at levels previously not observed with other DNA vaccine approaches and similar to or superior to responses induced by live vectors [32-39]. The importance of electroporation to the successful clinical translation of DNA vaccines was recently highlighted and emphasized in a high-profile review in *Nature Reviews* [40], and in the introduction to a special issue of *Vaccine* [41]. We have established a collaboration with Ichor Medical Systems and will use the TDS-IM electroporation device in the proposed phase 1 clinical trial.

2.7 Rationale for testing in adjuvant setting

To date the majority of therapeutic cancer vaccine trials have been performed in patients with metastatic disease. Unfortunately, the results of cancer vaccines in patients with metastatic cancers have been disappointing. In a recent review of cancer vaccine trials of 440 patients at the NCI, Rosenberg et al. noted that the objective clinical response rate by standard RECIST criteria was only 2.6% [42]. It is now commonly believed that generation of an effective antitumor immune response will be difficult in patients with metastatic cancers [43]. Metastatic breast cancer is no exception; metastatic breast cancer is associated with an increase in the prevalence of regulatory T cells [44, 45] and immature myeloid cells [46, 47] in the peripheral blood and in the tumor microenvironment, and these cells are capable of inhibiting endogenous or elicited antitumor immune responses.

These issues have resulted in a fundamental reassessment of the clinical development paradigm for therapeutic cancer vaccines, with an emphasis on early assessment of vaccine safety and efficacy in an appropriate clinical context [48-50].

The most important consideration in the design of this clinical trial is to ensure the safe translation of the personalized synthetic long peptide vaccine strategy. The Food and Drug Administration (FDA) dictates that initial studies of biologic therapies be performed in such a way that there is a balance between the

potential risks and benefits in individual patients. Consistent with these recommendations, we will target patients with triple-negative breast cancer who do not have a pathologic complete response after neoadjuvant chemotherapy. These patients typically have no gross evidence of disease following standard of care therapy (neoadjuvant chemotherapy, surgery and radiation therapy) but are at extremely high-risk for disease recurrence [51]. Targeting this patient population provides a window-of-opportunity to design and manufacture the personalized cancer vaccines, maximizes the potential benefit from the vaccine as the regulatory networks associated with metastatic disease are not present, and balances risk in this patient population with extremely high risk for disease recurrence but no other treatment options.

3 PATIENT SELECTION

3.1 Inclusion criteria

A patient will be eligible for inclusion in this study only if ALL of the following criteria apply:

(1) Histologically confirmed diagnosis of invasive breast cancer.

(2) ER and PR less than Allred score of 3 or less than 1% positive staining cells in the invasive component of the tumor. Patients not meeting this pathology criteria, but have been clinically treated as having TNBC, can be enrolled at PI discretion.

(3) HER2 negative by FISH or IHC staining 0 or 1+.

(4) Consented for genome sequencing and dbGAP-based data sharing and has provided or will provide germline and tumor DNA samples of adequate quality for sequencing. Fresh tissue is preferred (from biopsy at the time of port placement) but archival tissue is allowed.

(5) Clinical stage T1c-T4c, any N, M0 primary tumor by AJCC 7th edition clinical staging prior to neoadjuvant chemotherapy, with residual invasive breast cancer after neoadjuvant therapy. If the patient has invasive cancer in the contralateral breast, she is not eligible for this study.

(6) Completed all standard of care therapy (surgery + radiation as clinically necessary) prior to vaccination.

(7) At least 18 years of age.

(8) Eastern Cooperative Oncology Group (ECOG) performance status ≤ 2.

(9) Adequate organ and marrow function no more than 14 days prior to registration as defined below:

WBC	≥3,000/μL
absolute neutrophil count	≥1,500/μL
platelets	≥100,000/μL
total bilirubin	≤2.5 X institutional upper limit of normal
AST/ALT	≤2.5 X institutional upper limit of normal
creatinine	≤1.5 X institutional upper limit of normal

(10) Women of reproductive potential must agree to use adequate contraception (hormonal or barrier method of birth control; abstinence) prior to study entry and for the duration of study participation.

(11) Able to understand and willing to sign an IRB-approved written informed consent document.

3.2 Exclusion criteria

A patient will be ineligible for inclusion in this study if ANY of the following criteria apply:

(1) Evidence of progressive breast cancer within the last 30 days.

(2) Received chemotherapy, radiotherapy, or biologic therapy within the last 30 days (neoadjuvant chemotherapy excluded).

(3) Experiencing any clinically significant adverse events above Grade 1 (according to CTCAE 4.0) due to agents administered more than 30 days earlier. However, patients with Grade 2 Alopecia will be considered eligible.

(4) Receiving any other investigational agent(s) or has received an investigational agent within the last 30 days.

(5) Known metastatic disease.

(6) Invasive cancer in the contralateral breast.

(7) Known allergy, or history of serious adverse reaction to vaccines such as anaphylaxis, hives, or respiratory difficulty.

(8) Uncontrolled intercurrent illness including, but not limited to ongoing or active infection, symptomatic congestive heart failure, unstable angina pectoris, cardiac arrhythmia (including sinus bradycardia), or psychiatric illness/social situation that would limit compliance with study requirements.

(9) Prior or currently active autoimmune disease requiring management with immunosuppression. This includes inflammatory bowel disease, ulcerative colitis, Crohn's disease, systemic vasculitis, scleroderma, psoriasis, multiple sclerosis, hemolytic anemia, immune-mediated thrombocytopenia, rheumatoid arthritis, systemic lupus erythematosus, Sjögren's syndrome, sarcoidosis, or other rheumatologic disease or any other medical condition or use of medication (e.g., corticosteroids) which might make it difficult for the patient to complete the full course of treatments or to generate an immune response to vaccines. Asthma or chronic obstructive pulmonary disease that does not require daily systemic corticosteroids is acceptable. Any patients receiving steroids should be discussed with the PI to determine if eligible.

(10) Pregnant or breastfeeding. A negative serum pregnancy test is required no more than 7 days before study entry.

(11) The patient with a previous history of non-breast malignancy is eligible for this study only if the patient meets the following criteria for a cancer survivor. A cancer survivor is eligible provided the following criteria are met: (1) patient has undergone potentially curative therapy for all prior malignancies, (2) patients have been considered disease free for at least 1 year (with the exception of basal cell or squamous cell carcinoma of the skin or carcinoma-in-situ of the cervix).

(12) Patient must have no active major medical or psychosocial problems that could be complicated by study participation.

(13) Known HIV-positive status. These patients are ineligible because of the potential inability to generate an immune response to vaccines.

(14) Subjects with a strong likelihood of non-adherence such as difficulties in adhering to follow-up schedule due to geographic distance from the Siteman Cancer Center should not knowingly be registered.

(15) Individuals in whom a skinfold measurement of the cutaneous and subcutaneous tissue for eligible injection sites (left and right medial deltoid region) exceeds 40 mm.

(16) Individuals in whom the ability to observe possible local reactions at the eligible injection sites (deltoid region) is, in the opinion of the investigator, unacceptably obscured due to a physical condition or permanent body art.

(17) Therapeutic or traumatic metal implant in the skin or muscle of either deltoid region.

(18) Acute or chronic, clinically significant hematologic, pulmonary, cardiovascular, or hepatic or renal functional abnormality as determined by the investigator based on medical history, physical examination, EKG, and/or laboratory screening test

(19) Any chronic or active neurologic disorder, including seizures and epilepsy, excluding a single febrile seizure as a child

(20) Syncopal episode within 12 months of screening

(21) Current use of any electronic stimulation device, such as cardiac demand pacemakers, automatic implantable cardiac defibrillator, nerve stimulators, or deep brain stimulators.

3.3 Inclusion of women and minorities

Women of all races and ethnic groups are eligible for this trial.

4 REGISTRATION PROCEDURES

4.1 Prior to registration

4.1.1 Subject recruitment

The methods used for recruitment of subjects in the study will be devoid of any procedures that may be construed as coercive. The recruitment process will not involve any restrictions based on social or demographic factors including age, or ethnic characteristics of the subject population. However, the composition of the study subject population will depend on patient sources available to the investigators. Subjects will be identified and recruited for this study as follows:

Patients will be recruited from Siteman Cancer Center outpatients or patient referrals by our community oncologists to the principal investigator and co-investigators. Patients must be willing and able to give their written informed consent indicating that they are aware of the investigational nature of the study. After a patient is deemed eligible for study, the principal investigator (or co-investigators) will discuss the Washington University Human Research Protection Office-approved informed consent with the patient. This written informed consent will be signed and dated by the patient and the principal investigator (or co-investigators). The original consent will be placed in the patient's permanent record and a copy will be given to the patient.

Washington University School of Medicine (WUSM) has an approved Multiple Project Assurance of Compliance with Department of Health and Human Services Regulations for the Protection of Human Research Subjects on file with the Office for Human Research Protection (OHRP). The Human Research Protection Office Policies and Procedures for Protection of Human Research Subjects details all policies and procedures for the protection of human research subjects and can be obtained upon request from the Human Research Protection Office.

4.1.2 Compliance and understanding

All patients who present with locally advanced breast cancer will be screened for eligibility for entry into the study. As in all trials, the goal is to achieve a high level of compliance with protocol requirements by assuring, during the eligibility assessment, that the potential subject is fully informed and agrees to the protocol requirements. In addition, subjects with a strong likelihood of non-adherence such as difficulties in adhering to follow-up schedule due to geographic distance from the Siteman Cancer Center, should not knowingly be registered. Adherence of the Siteman Cancer Center staff to careful assessment of the subject's understanding of the trial and a clinical center environment which supports the continued commitment of the subjects are essential for the trial to be successfully completed.

4.1.3 Presentation of informed consent

Consent will be obtained by either the principal investigator or by individuals approved by the principal investigator and whose names and copy of their *curriculum vitae* have been filed. The initial consent should be the IRB-approved version corresponding to the version of the protocol approved when the screening was initiated. Informed consent is to be obtained from the subject according to **Section 14.1 Informed Consent** of this protocol.

4.2 Registration procedures

Patients must not start any protocol intervention prior to registration through the Siteman Cancer Center.

The following steps must be taken before registering patients to this study:

(1) Confirmation of patient eligibility

(2) Registration of patient in the Siteman Cancer Center OnCore database

(3) Assignment of unique patient number (UPN)

4.3 Confirmation of patient eligibility

Confirm patient eligibility by collecting the information listed below:

(1) The registering MD's name

(2) Patient's race, sex, and DOB

(3) Three letters (or two letters and a dash) for the patient's initials

(4) Copy of signed consent form

(5) Completed eligibility checklist, signed and dated by a member of the study team

(6) Copy of appropriate source documentation confirming patient eligibility

4.4 Patient registration in the Siteman Cancer Center OnCore database

All patients must be registered through the Siteman Cancer Center OnCore database.

4.5 Assignment of UPN

Each patient will be identified with a unique patient number (UPN) for this study. Patients will also be identified by first, middle, and last initials. If the patient has no middle initial, a dash will be used on the case report forms (CRFs). All data will be recorded with this identification number on the appropriate CRFs.

5 INVESTIGATIONAL AGENT

5.1 Chemical name and structure

The personalized polyepitope DNA vaccines are also known as DNA plasmid vector expressing tumor-specific antigens.

Polyepitope DNA vaccines will be designed in the Gillanders laboratory and manufactured at the Siteman Cancer Center Biological Therapy Core Facility based on the following general steps:

(1) Breast cancer tissue and normal lymphocytes will be obtained from breast cancer patients who are eligible for the phase 1 clinical trial.

(2) Tumor/normal exome sequencing and tumor cDNA-capture sequencing will be performed to identify candidate mutant tumor-specific antigens.

(3) Candidate mutant tumor-specific antigens will be prioritized based on epitope prediction algorithms and *in vitro* studies.

(4) Personalized polyepitope inserts integrating the prioritized mutant tumor-specific antigens will be designed in the Gillanders laboratory and then synthesized and cloned into the pING parent vector by Blue Heron Biotech.

(5) Master cell banks will be established and validated at WUSM.

(6) The personalized polyepitope DNA vaccines will be manufactured and vialed at WUSM.

(7) The personalized polyepitope DNA vaccines will undergo product release tests at WUSM and other sites prior to investigational use.

The personalized polyepitope DNA vaccines will be manufactured in the SCC Biologic Therapy Core facility. Standard Operating Procedures for the GMP manufacture of the personalized vaccines have been established and are in accordance with “CGMP for Phase 1 Investigational Drugs 2008,” and “Considerations for Plasmid DNA Vaccines for Infectious Disease Indications 2007.”

Each polyepitope DNA vaccine drug product is composed of a deoxyribonucleic acid (DNA) plasmid purified from *E. Coli*. The pING parent vector was obtained from Alan Houghton, M.D. at Memorial Sloan Kettering Cancer Center (Figure 2). The pING vector has been used extensively in preclinical DNA vaccine studies [52, 53], and in human clinical trials, including a clinical trial at WUSM (ClinicalTrials.gov identifier: NCT00807781). The mammaglobin-A DNA vaccine is based on the pING vector and was manufactured at WUSM in the SCC Biologic Therapy Core Facility using manufacturing processes almost identical to the ones outlined here).

The pING vector contains the following elements: (1) a eukaryotic promoter and enhancer from the Towne strain of CMV; (2) a polylinker region to facilitate cloning of a variety of DNA fragments; (3) donor and acceptor splice sites and a poly adenylation signal sequence derived from the bovine growth hormone gene; (4) the ColE1 origin of replication and (5) a gene conferring kanamycin resistance. With the exception of the kanamycin resistance gene, which was cloned as a PstI fragment from the plasmid pUC4, all other gene segments were amplified by polymerase chain reaction (PCR).

The role of each element is as follows: (1) the CMV promoter/enhancer enables high-level expression of polyepitope insert in mammalian cells; (2) the polylinker region serves as a multiple cloning site for easy insertion of genes, in this case the polyepitope insert; (3) the polyadenylation signal sequence facilitates efficient transcription termination and polyadenylation of mRNA; (4) the origin of replication allows for high-copy number replication and growth in *E. coli* and (5) the kanamycin resistance gene provides for selection in *E. coli*. Of note, the kanamycin resistance gene is cloned in the opposite orientation from the polyepitope insert to limit transcription of kanamycin from the CMV promoter in human cells.

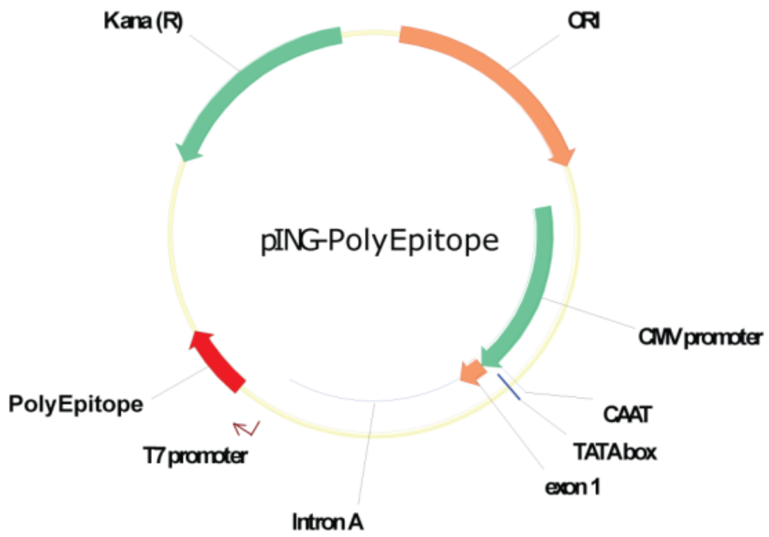

Figure 2: Polyepitope DNA vaccine design. Polyepitope DNA vaccines are based on the pING parent vector. The pING vector contains the following elements: (1) a eukaryotic promoter and enhancer from the Towne strain of CMV; (2) a polylinker region to facilitate cloning of a variety of DNA fragments; (3) donor and acceptor splice sites and a poly adenylation signal sequence derived from the bovine growth hormone gene; (4) the ColE1 origin of replication; (5) a gene conferring kanamycin resistance, and (6) a polyepitope insert integrating the prioritized mutant tumor-specific antigens.

The personalized polyepitope inserts will be designed in the Gillanders Laboratory based on the results of next-generation sequencing and epitope prediction algorithms from each individual patient. Personalized polyepitope inserts will incorporate up to 20 prioritized mutant tumor-specific antigens. The personalized polyepitope inserts will be synthesized by Blue Heron Technologies and cloned into the pING parent vector as detailed below.

5.2 Manufacturing facility

The personalized polyepitope DNA vaccines will be prepared in the Siteman Cancer Center Biologic Therapy Core Facility at Washington University School of Medicine. The facility is located at 500 S. Kingshighway, Room 719 Southwest Tower, Saint Louis, MO 63110.

The facility adheres to cGMP practices with regard to documentation, facility maintenance, and QC/QA review. Within the 2,615 sq ft GMP-facility on the 7th floor of the Southwest Tower, 6 manufacturing rooms are available for clinical grade manufacturing of cellular therapy products, recombinant DNA or gene therapy products (Floor plan below).

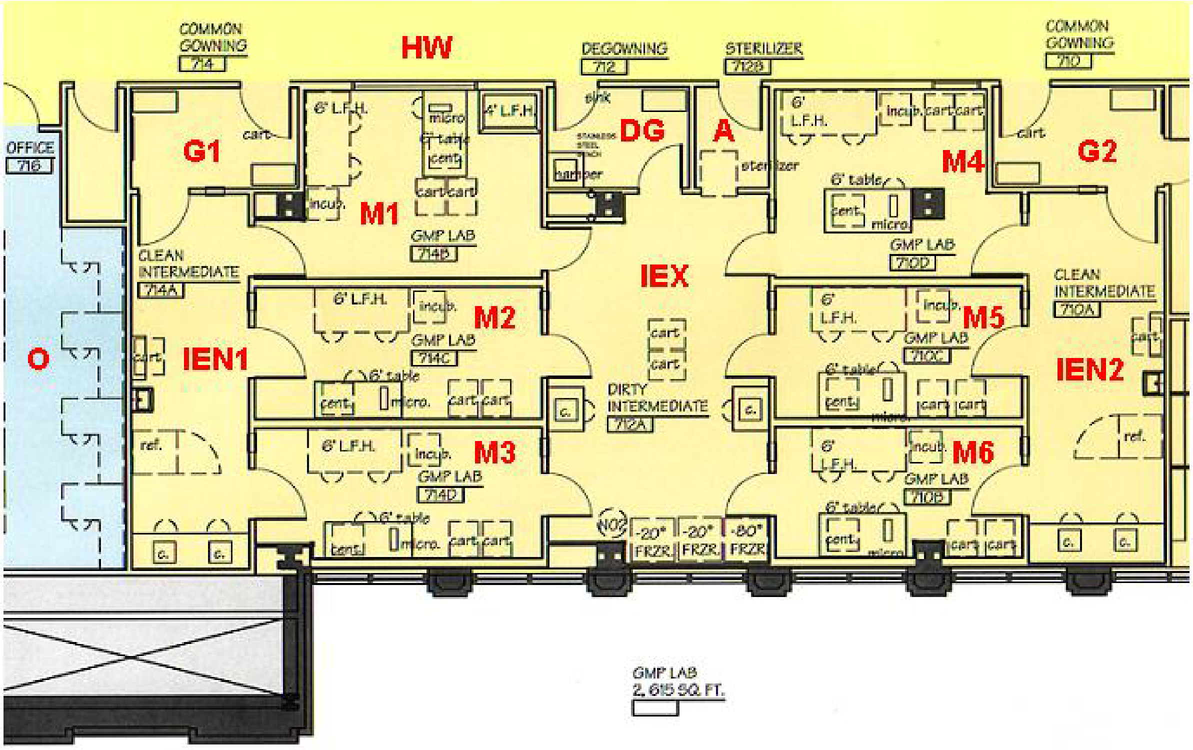

Figure 3: Siteman Cancer Center Biologic Therapy Core Facility floor plan. HW: Hallway, G1: Gowning Room 1; G2: Gowning Room 2; IEN 1: Intermediate Entry Room 1; IEN 2: Intermediate Entry Room 2; M1 to M3: Manufacturing Rooms 1 to 3; M4 to M6: Manufacturing Rooms 4 to 6; IEX: Intermediate Exit Room; DG: Degowning Room; O: Office; A: Autoclave Room.

In the Biologic Therapy Core facility, all manufacturing rooms are physically separated from each other with single pass air, and provide one-way personnel and product flow, which avoids cross-contamination of products. 6 different products can be worked on simultaneously without interfering with each other. Passthroughs are available from gowning rooms to intermediate rooms, and intermediate rooms to manufacturing rooms. All manufacturing rooms are completely isolated from each other by sealed walls and ceilings. A custom ceiling allows maintenance on the HVAC units, filters or lights without breeching the clean environment of the facility.

The manufacturing laboratories are only entered, one at a time, from an intermediate room. The laboratories are only exited, one at a time, into another intermediate room.

This provides easily manageable spatial and temporal segregation of products, which is a necessity to prevent product cross-contamination. Door interlocks, which allow for doors to be opened only one at a time, assure this unidirectional flow of personnel and products.

Manufacturing rooms, intermediate rooms and gowning/degowning rooms are isolated from each other by air pressure gradients. The manufacturing rooms have higher air pressure in respect to the intermediate rooms; gowning rooms have negative air pressure towards intermediate rooms and the hallway. This also assures protection of the outside environment from potentially biohazardous materials produced in the Biologic Therapy Core facility. Air pressure gradients are maintained by individual HVAC units for each room. The manufacturing rooms are certified and maintained as Class 10,000, the intermediate rooms are certified and maintained as Class 100,000.

Every manufacturing lab is equipped with a 6-foot biosafety cabinet and a dual chamber incubator. Sufficient room is available per manufacturing lab to accommodate specialized equipment (e.g. clinical grade magnetic bead cell separator) for different manufacturing processes. In the largest manufacturing room, besides the 6ft biosafety cabinet, a second 4ft biosafety cabinet is installed for work with infectious agents. Manufacturing room 1 can also be switched to negative air pressure and BSL-3 containment (vector manufacturing), and an IBC approved protocol to accomplish this task is in place.

Quality control and monitoring plans are in effect. Daily environmental cleaning is performed by dedicated personnel. The facility is electronically monitored continuously, daily monitoring by core personnel ensures adherence to standards and is prescribed by SOPs. Environmental monitoring includes non viable and viable particle enumeration with limits more stringent than the standards prescribed in the USP.

GMP regulations for even Phase 1 clinical trials are in place to assure a cellular or biological product to be safe and free of contaminants.

5.3 Manufacturing process

Overview of the polyepitope DNA vaccine manufacturing process. Schematic outline of product manufacture, in-process testing, and product release tests.

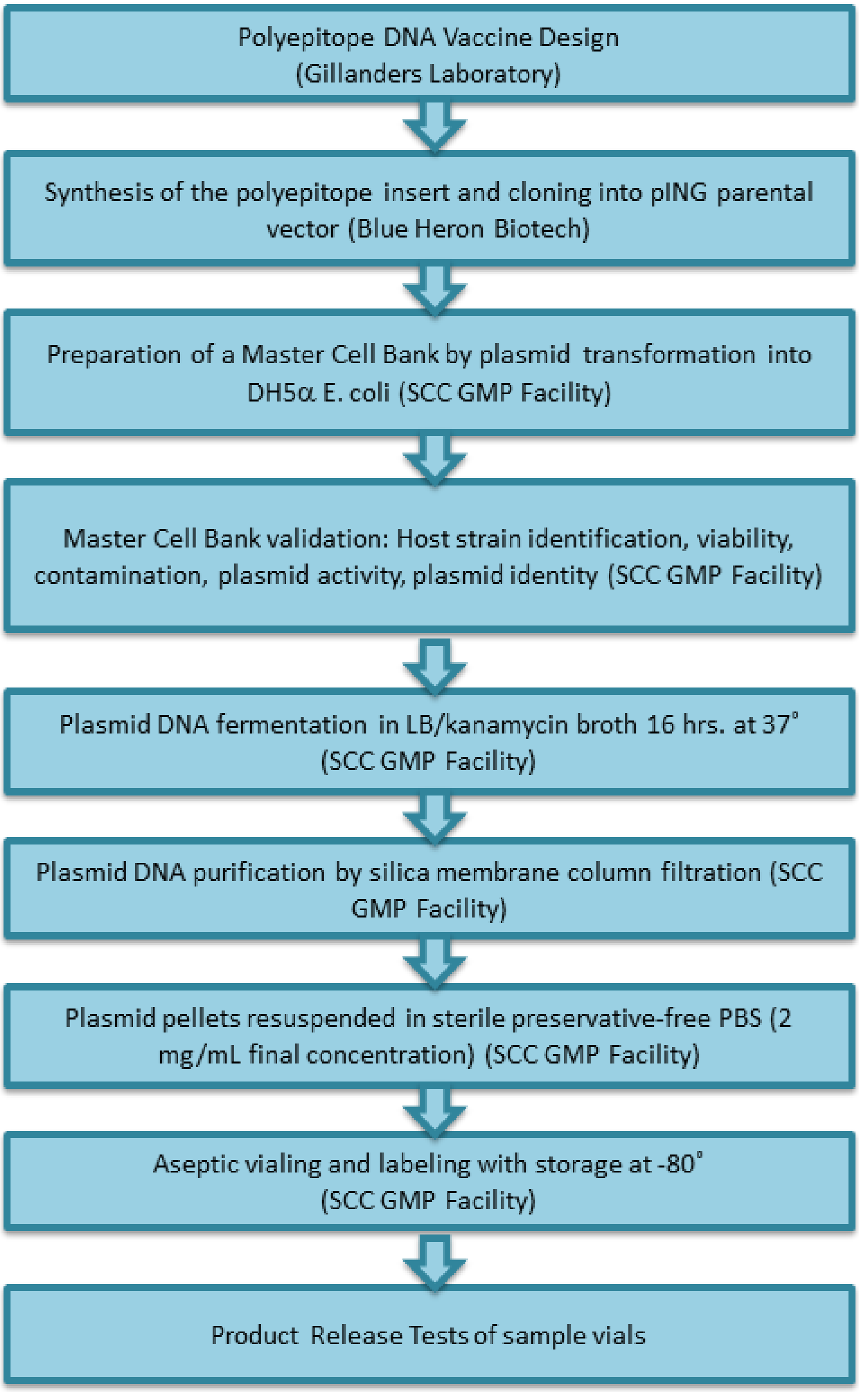

Figure 4: Overview of the polyepitope DNA vaccine manufacturing process. Schematic outline of product manufacture, in-process testing, and product release tests.

Design of the polyepitope DNA vaccine. Personalized polyepitope DNA vaccines will be designed in the Gillanders laboratory at WUSM. Up to 20 candidate mutant tumor-specific antigens from each patient will be integrated into the polyepitope DNA vaccine. Mutant tumor-specific antigens will be identified and prioritized based on the results of next-generation sequencing studies, epitope prediction algorithms, and *in vitro* studies.

Synthesis of the polyepitope insert. A polyepitope insert integrating up to 20 of the highest priority mutant tumor-specific antigens will be designed in the Gillanders laboratory. This insert will then be synthesized by Blue Heron Biotech. The insert will then be cloned into the pING parent vector by Blue Heron Biotech. The pING vector containing the polyepitope insert will be sequenced by Blue Heron Biotech. The plasmid will then be used to establish a master cell bank at WUSM as detailed below.

Creation of a master cell bank. Master cell banks for each individual polyepitope DNA vaccine will be constructed by transformation of *E. coli* strain DH5 alpha with each patient's unique pING-polyepitope plasmid. Briefly, 15 vials of transformed bacteria will be prepared and stored at -80°C in the Siteman Cancer Center Biologic Therapy Core Facility. The Johnson Controls monitoring system continuously monitor the freezer used for storage. The system triggers an alarm and electronically notifies staff when temperature values are out of specification. In addition, daily manual monitoring is applied. For each bacterial culture production, a vial of the glycerol stock will be taken from the master cell bank and used for culture inoculation.

Samples from each master cell bank will be characterized for host strain identity, contaminating organisms, viable cells, plasmid activity, and plasmid identity as detailed below in testing to be performed under GLP conditions at Barnes-Jewish Hospital and Washington University School of Medicine. Of note, in previous sequencing reactions we have identified three variations in pING vector sequence. These variations are in non-coding areas of the plasmid DNA, and are not expected to have any functional significance. They are also present in the pING parent vector obtained from MSKCC, suggesting that the published sequence for the pING vector may not be accurate. Please see Table 1 below.

TABLE 1: Master cell bank validation for polyepitope DNA vaccines

Criteria	Method	Specification	Test site
Host strain identity	Growth on selective media	Growth of *E. coli*	Barnes-Jewish Hospital Microbiology Laboratory
Contaminating organisms	Growth on selective media,	No growth	Barnes-Jewish Hospital Microbiology Laboratory
Viable cells	Growth in LB agar	Growth of *E. coli*	Barnes-Jewish Hospital Microbiology Laboratory
Plasmid activity	Transfect cells, RT-PCR	Detection of polyepitope construct mRNA	Gillanders Laboratory
Plasmid identity	DNA sequencing	Sequence as predicted	Gillanders Laboratory
Plasmid identity	Restriction analysis	As expected, no extraneous bands	Gillanders Laboratory

Plasmid DNA fermentation. The plasmid vectors will be grown in *E. Coli* strain DH5alpha. The host bacteria will be grown in 500 mL LB broth cultures in a bacterial shaker at 37°C for 16 hours. Antibiotic selection (kanamycin) will be applied to increase plasmid yield.

Plasmid DNA purification. The plasmid DNA will be purified using reagents obtained from Sigma Aldrich (Saint Louis, MO). Distinct lots of the Genelute HP Select Plasmid Gigaprep Kit from Sigma-Aldrich (St.

Louis, MO) will be used for the manufacture of the plasmids. The following narrative is a brief description of the protocol. The composition of buffers, solutions, and chromatography material is included in Table 2 below. (Please note that the precise composition of the resin and solutions are considered by Sigma Aldrich to be proprietary). The harvested bacteria will be recovered by centrifugation, and then resuspended in a cell suspension buffer containing RNase A. Lysis buffer will be added and a homogenous lysate will be obtained by gentle inversion. After neutralization of the bacterial lysate, a lysate clearing agent will be added and allowed to stand for 5 minutes for precipitates to form. The lysate will be cleared by filtration, and a binding solution optimized for endotoxin-free plasmid purification will be added. The lysate with binding solution will then run through an equilibrated column where the plasmid DNA is captured onto a silica membrane in the presence of high salt, while endotoxins are prevented from adsorbing to the membrane. The flow-through will be discarded, and the contaminants in the column will be removed in three wash steps. Finally, the bound plasmid DNA will be eluted in endotoxin-free water. The eluted DNA will be precipitated by adding 0.1 volume of 3 M sodium acetate and 0.7 volumes of isopropanol. The DNA will be recovered by centrifugation at 14,000 x g in a fixed angle rotor. A wash and pelleting of the final DNA with 70% ethanol will be performed and the resulting purified DNA will be air dried in a biosafety cabinet. All vessels and buffers used for DNA preparation will be sterile for single use, or will be autoclaved in a monitored and quality controlled autoclave in the Biological Therapy Core facility.

The only product of animal origin is the RNase solution. This is isolated from bovine pancreas and we have Certificates of Origin that clearly indicate that these were derived from a New Zealand herd. These animals passed ante and post mortem testing for viral pathogens; the samples were treated with low pH (1.7) for 12 hours to inactivate pathogenic viral agents; and no animal protein was fed to these animals.

Glycerol will be used in our manufacturing process. However, all Lot #s of the glycerol utilized will be of plant origin.

**TABLE 2: Composition of buffers, solutions, and chromatography material**	
Solution	Composition, Sigma #
Wash Solution 2	Tris-HCl (T3038), NaCl (S5150)
Neutralization Solution	Trizma-HCl (T5941)
Binding Column	Guanidine Thiocyanate (G9277), EDTA (E7889)
Column Preparation Solution	NaOH (S2770)
Resuspension/RNase A Solution	Tris-HCl (T3038), EDTA (E7889)
Lysis Solution	NaCl (S5150), Tris-HCl (T3038), EDTA (E7889), Ocytl-beta-Thioglucopyranoside (O6004)
Lysate Clearing Agent	Guanadine HCl (G3272), Tris-HCl (T2413), EDTA (E7889), Tween20 (P7949), Triton X-100 (T8787), Hydrochloric acid (H9892)
Binding Solution	Guanadine HCl (G3272), Tris Acetate (T1258), Isopropanol (I0398), NaOH (S2770)
Column Prep Solution	NaOH (S2770)
EndoCleaning Solution	NaOH (S2770), Tween20 (P7949)
Wash Solution 1	NaCl (S5150), Tris-HCl (T3038)
Wash Solution 2	Tris-HCl (T3038), NaCl (S5150)

5.4 Product formulation

The eluted plasmid preparations will be resuspended in sterile, preservative free-PBS. A small sample (100 µL) will be removed aseptically and the concentration tested using a certified NanoDrop spectrophotometer, Qubit Fluorometer or similar device. Typically, the eluted DNA is in the range of 3-4 mg/mL. The DNA product will be diluted to 2 mg/mL using sterile, preservative-free PBS, and aseptically added to 2.0 mL cryovials from Nalgene (Catalog# 5000-0020, Lot# 616412). The vials are made of polypropylene with a high-density polyethylene closure. They are externally-threaded for aseptic technique. They have a sealing ring in conjunction with specially designed threads. A white marking area, fill line, and graduations are printed on the vials with white paint. They are radiation-sterilized, non-cytotoxic, non-pyrogenic, and are RNase/DNase-free.

We plan to synthesize enough DNA for approximately 20 vials. We anticipate that 6 vials will be required for vaccination (2 vials per time point times 3 timepoints), and 5 vials will be required for product release tests. We are targeting 20 vials to have a reserve stock.

Following vialing, we anticipate that 5 vials (#1, 4, 10, 19) will be used for product release testing. Vials 1 and 19 will be used to test sterility and represent the beginning and end of the vialing procedure. Two additional vials will be used for the other product release tests listed in Table 5.

If any contamination would be detected in our plasmid samples, we would initiate an alcohol precipitation step. This procedure is basically the final stage of the gigaprep purification, where the soluble DNA is

precipitated using 2x volumes of cold isopropanol and the DNA is pelleted by centrifugation. The pellet is washed in 70% ethanol and air dried. This procedure is a standard method for eliminating any microbiologic contamination in DNA.

5.5 Product release tests

A key issue in the quality control of plasmid DNA for human use is the development of analytical methodologies capable of fully characterizing the product in its final form, ensuring the production of a consistent product. The following list of release criteria, analytical methodologies, and specifications has been adapted from [54]. Polyepitope DNA vaccines will be tested for the following specifications.

TABLE 3: Polyepitope DNA vaccine product release tests

**Criteria**	**Method**	**Specification**	**Test Site**
Plasmid activity	Transfect cells, RT-PCR	Polyepitope mRNA present	Gillanders Laboratory
Residual kanamycin	USP 29 <81>	< 24 mcg/mL	Alcami or equivalent
Plasmid homogeneity	Agarose gel electrophoresis	≥80% closed circular	Gillanders Laboratory
Residual RNA	Agarose gel electrophoresis	Less than 1%	Gillanders Laboratory
Residual bacterial chromosomal DNA	Agarose gel electrophoresis	Less than 1%	Gillanders Laboratory
Plasmid identity	Restriction analysis	As expected, no extraneous bands	Gillanders Laboratory
Appearance	Visual	Clear and colorless, free of particulates	Gillanders Laboratory
pH	Calibrated pH meter	Report pH	Biologic Therapy Core Facility
DNA concentration	Nano-drop spectrophotometer	Greater than 1 mg/mL	Gillanders Laboratory
Residual proteins	BCA reagent colorimetric	Less than 1% (< 21 µg/mL)	Gillanders Laboratory, Biologic Therapy Core Facility, or equivalent
Residual isopropanol, EtOH	USP <467>	Less than 5000 ppm	PACE Analytics or equivalent
Endotoxin	Chromogenic Limulus amebocyte lysate assay	Less than 175 EU/mL	Biologic Therapy Core Facility
Sterility	USP <71>	No growth	Biologic Therapy Core Facility

Alternatively, we will perform studies to demonstrate that the manufacturing process removes residual kanamycin, RNA, bacterial chromosomal DNA, proteins, isopropanol and EtOH below the specification levels, and not perform these residual studies on each lot. Update, July 2017: to date, testing on each lot has shown that residual kanamycin is undetectable and residual isopropanol and EtOH is consistently below the specification. This testing will no longer be performed. The criteria for sterility meets specification if no growth after 7 days. The sterility culture will be held and monitor for 14 days as per USP <71>.

5.6 Stability

The polyepitope DNA vaccines will be stored in the Biologic Therapy Core Facility in a -80°C freezer. The freezer used for storage is monitored 24/7 by Johnson Controls monitoring system with dedicated on-call personnel to address any aberrant occurrences. Overall, we anticipate no problems with plasmid stability as stability is considered to be one of the advantages of the DNA vaccine platform, and plasmid DNA formulated in PBS is considered to be very stable.

We are currently storing similar investigational DNA vaccines in the Biologic Therapy Core Facility in a -80°C freezer. These investigational DNA vaccines were manufactured in the Biologic Therapy Core Facility using an almost identical manufacturing process as the one proposed here, are based on the pING parental vector and are thus very similar in size and composition, and are formulated similarly in PBS at 2 mg/mL. These DNA vaccines have remained stable over a 3 year period.

We do not anticipate that long-term stability testing will be required. As each lot of vaccine is personalized to a specific patient, we anticipate that the vaccine will be used within 6 months of completion of the product release tests.

5.7 Labels

Each polyepitope DNA vaccine product will be made as a single lot ensuring that the labeled product is unique and consistent for this trial. Please see the sample label below. Prior to administration a time out will be performed to confirm subject identity, and the investigational agent to be administered. This will be performed by the clinical research associate, the oncology nurse trained to administer the vaccine by electroporation and the experimental pharmacist.

SCC Biologic Therapy Core Facility
Saint Louis, MO
Store at -80°C
WUSM PPDV #1

Subject Study #, Subject Initials

LOT #	1
Vial #	003
Concentration	2mg/mL
Volume	1.2mL
Date	mm/dd/yyyy

CAUTION Investigational drug limited by federal law to investigational use

5.8 Siteman Cancer Center Investigational Pharmacy

The Investigational Pharmacy is located on the 7th floor of the Center for Advanced Medicine within the chemotherapy administration area of the Siteman Cancer Center. The pharmacy is locked and secured with entry only via an authorized swipe card entry system. Three pharmacists, including a pharmacist with responsibility for investigational agents, and two technicians are employed full-time. Two freezers (-70ºC and -20ºC) and one refrigerator have been designated for studies. Room temperature shelving extends over 300 feet. There are three IV admixture hoods. Two vertical flow hoods are used for preparation of chemotherapy agents and a laminar flow hood is used for non-chemotherapy agents. There were a total of 13,260 chemotherapy prescriptions filled in 2004. Pharmacy hours of operation are Monday through Friday from 7:00 am to 5:30 pm. The investigational pharmacist will obtain the vaccine from the TBC Facility and dispense the vaccine to the nurse who will be administering the agent to the patient.

5.9 Investigational agent administration

Each DNA vaccination will be 1 mL vaccine administered intramuscularly using an integrated electroporation administration system (TDS-IM system, Ichor Medical Systems). At each vaccination time point, patients will receive two injections at separate sites.

5.9.1 Introduction to electroporation

Electroporation (EP) is a potent delivery technique based on the in vivo application of electrical fields that may improve immune responses following the administration of plasmid DNA vaccines. The EP effect is induced through the propagation of electrical fields of sufficient magnitude and duration within a target region of tissue. These electrical fields produce a transient increase in the membrane permeability of cells exposed to the electrical field, allowing enhanced intracellular uptake of agents distributed within the interstitium of the local tissue. Of note, agent delivery occurs only where the agent of interest is present contemporaneously with the propagation of threshold level electrical fields. Numerous non-clinical studies have demonstrated that the application of EP can enhance gene expression and immune responses following plasmid DNA vaccine delivery.

Although DNA vaccines encoding antigens from a variety of cancers and infectious pathogens have exhibited considerable promise in non-clinical studies, clinical experience to date suggests that current administration methods may have insufficient potency to achieve the desired immune responses in humans. Poor delivery efficiency and low levels of antigen expression have been implicated as factors contributing to the sub-optimal responses observed to date [55, 56].

Electroporation (EP) is a potent physical delivery technique based on the *in vivo* application of electrical fields. The propagation of electrical fields of a sufficient magnitude within a target tissue induces a transient increase in cell membrane permeability, resulting in a significant improvement of intracellular uptake of exogenous substances. Shortly after pulse delivery, the cell membrane function stabilizes and cells within the affected tissue resume normal function. EP has been shown in non-clinical studies to be a potent method for delivery of DNA vaccines [37, 57-59].

EP has several important advantages that make it an appealing method for delivery of DNA vaccines. First, EP can reliably enhance delivery and expression of DNA antigens by 2-3 orders of magnitude. This improvement in delivery has been shown to enhance both cellular and humoral immune responses. Second, EP is a non-viral delivery method. As such, it has a favorable safety profile and does not elicit unwanted immune responses against the vector. Thus, the technique is particularly appropriate for indications likely to require multiple vaccine administrations to achieve response (e.g. prime / boost). It is also advantageous because the absence of immunogenic viral antigens minimizes potentially deleterious antigen competition, thereby ensuring that the immune response is concentrated on the antigen of interest. Lastly, unlike other non-viral approaches, EP is capable of inducing consistent responses from subject to subject, even at relatively low DNA doses.

5.9.2 The TDS-IM Electrode Array System

The TDS-IM device is supplied by Ichor Medical Systems, Inc. The TDS-IM device utilizes the in vivo application of electrical fields to enhance the intracellular delivery of agents of interest in a targeted region of tissue (EP). The device complies with the applicable safety and electromagnetic compatibility requirements of International Electrotechnical Commission (IEC) 60601-1. During this study, the device will be operated according to the TDS-IM User Manual and applicable study-specific procedures to ensure consistent and safe utilization.

Each TDS device consists of 3 parts: A pulse stimulator, an integrated applicator, and a single-use application cartridge (see Figure below). The components are manufactured in ISO13485-compliant, FDA-registered facilities including BIT MedTech, LLC. (Pulse Stimulator) and Life Science Outsourcing, Inc. (Integrated Applicator and Application Cartridges.

TDS-IM Components

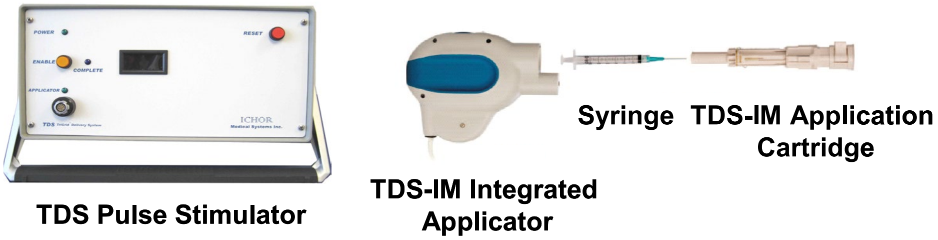

5.9.3 Summary of TDS-IM Clinical Experience to Date

To date, formal safety studies have been conducted to assess the TDS-IM as the means for delivery of nine DNA vaccine candidates. This includes studies of a melanoma DNA vaccine encoding a xenogeneic form of the tyrosinase antigen that uses the same vector backbone (pING) as the Mammaglobin-A DNA vaccine candidate (see Table 1). Safety studies for the various DNA vaccine candidates have included repeat dose studies in rabbits to assess safety and toxicology, and studies in rats to assess DNA vaccine biodistribution, persistence, and potential for integration into host DNA.

In the safety and toxicology studies, notable findings associated with electroporation-mediated delivery of DNA vaccines were limited to localized inflammatory responses of mild to moderate severity at the site of administration (for an example, see Dolter, K.E., *et al.* Immunogenicity, safety, biodistribution and persistence of ADVAX, a prophylactic DNA vaccine for HIV-1, delivered by in vivo electroporation. Vaccine, 2011. 29(4): p. 795-803). The injection site findings were most prominent in tissue samples obtained 48–72 hours after administration at that site. Tissue samples obtained from administration sites 14–43 days after administration indicate progressive resolution of the local inflammatory responses over time.

The results of the biodistribution studies indicated negligible systemic uptake of vaccine DNA following EP-based intramuscular delivery, with no significant differences observed among the different DNA vaccine candidates tested to date. Persistence analysis indicated that the presence of vaccine DNA 30 to 90 days after administration was confined to the tissues at the site of administration (muscle and skin), and only at very low levels (< 1,000 copies/μg host genomic DNA), suggesting minimal risk for potential integration of vaccine DNA into host DNA (for an example, see Dolter, 2011).

Reports describing the results of the tyrosinase GLP safety and toxicology studies are available in BB IND 13275); a letter of cross reference to this IND has been provided by Ichor.

5.9.4 Summary of TDS-IM clinical experience to date

The TDS-IM investigational device is currently being used as the means for DNA vaccine delivery in both the therapeutic and prophylactic setting. Six clinical trials of the TDS-IM device have been completed as detailed in the Table below.

**TDS-IM Based DNA Vaccine Administration: Completed Clinical Trials**		
**ClinicalTrials.gov Study #**	**Vaccine Candidate**	**Subject Population**
NCT00545987	Multigenic HIV-1 DNA vaccine candidate (ADVAX)	Healthy, HIV uninfected adult volunteers
NCT00471133	Xenogeneic tyrosinase DNA vaccine candidate (pINGmuTyr)	Patients with Stage IIB-IV melanoma
NCT01169077	Multi-epitope malaria DNA vaccine (EP-1300)	Healthy adult volunteers
NCT01502345	Multi-antigen hantavirus DNA vaccine (pWRG/HTN-M(x) and pWRG/PUUV-M(s2))	Healthy adult volunteers
NCT01641536	Multi-antigen HBV DNA vaccine administered with a DNA-based human IL-12 adjuvant (HB-110)	HBV infected adult volunteers
NCT01496989	Multi-antigen HIV DNA vaccine (HIV-MAG) administered with or without a DNA-based human IL-12 adjuvant (GENEVAX) prior to or after administration of an adenovirus vector (Ad35GRIN)	Healthy, HIV uninfected adult volunteers

The TDS-IM device is currently being evaluated in seven ongoing clinical studies as detailed in the Table below.

**TDS-IM Based DNA Vaccine Administration: Ongoing Clinical Trials**		
**ClinicalTrials.gov Study #**	**Vaccine Candidate**	**Subject Population**
NCT01138410	Epitope-based TRP-2 melanoma vaccine (SCIB-1)	Patients that are HLA A1, A2, A24, or B35 positive with AJCC stage III-IV melanoma
NCT01859325	Multi-antigen HIV DNA vaccine (HIV-MAG) administered with or without a DNA-based human IL-12 adjuvant (GENEVAX) prior to administration of an vesicular stomatitis virus vector (rVSVgag)	HIV infected adult volunteers
NCT01578889	Multi-antigen HIV DNA vaccine (HIV-MAG) administered with or without a DNA-based human IL-12 adjuvant (GENEVAX) prior to administration of an vesicular stomatitis virus vector (rVSVgag)	Healthy adult volunteers
NCT01266616	Multi-antigen HIV DNA vaccine (HIV-MAG) administered with or without a DNA-based human IL-12 adjuvant (GENEVAX)	HIV infected adult volunteers
NCT01634503	Multi-antigen HPV DNA vaccine administered with a DNA-based human IL-12 adjuvant (GX-188E)	Patients with Grade 3 Cervical Intraepithelial Neoplasia
NCT01493154	HPV DNA vaccine administered with a DNA-based calreticulin adjuvant (pNGVL-4a-CRT/E7 (detox))	HPV-associated squamous cell carcinoma of the head and neck
NCT01984983	VEEV DNA Vaccine Candidate	Healthy adult volunteers

To date, the 13 clinical trials that have been completed or are currently ongoing have enrolled over 350 subjects in the electroporation arms of the studies (including subjects receiving either the DNA vaccine

candidate or placebo). The device has been used for administration of DNA injections of up to 1.0 ml volume and 4.0 mg DNA dose per injection site. Subjects have received the vaccine candidate either as a single injection in one muscle site (total DNA dose up to 4.0 mg per administration time point) or as 2 injections in 2 separate muscle sites (total DNA dose up to 8.0 mg per administration time point). Subjects given the DNA dose as a single injection have received up to 5 TDS-IM injections at up to 4.0 mg DNA, and subjects administered the DNA dose in 2 injections have received up to 5 administrations (i.e., 10 total TDS-IM injections).

Adverse responses reported in association with use of the device include localized muscle contractions and associated discomfort/pain during the application of EP, minor cutaneous bleeding at the site of injection, and transient injection site soreness of mild to moderate severity, typically resolving within 24–72 hours following administration. Several subjects in the 2 melanoma studies have reported lightheadedness immediately following procedure application which, in some cases, was accompanied by a decrease in blood pressure. In one subject, enrolled in the xenogeneic tyrosinase study, this was followed by a brief syncopal episode (~30 seconds duration) shortly after procedure application. The subject recovered without incident. At the time of enrollment, the subject indicated a life long history of sinus bradycardia of unknown origin, which was confirmed by electrocardiogram (EKG) during screening. Multiple EKGs performed after the syncopal episode indicated no changes from pre-procedure baseline. Based on the judgment of the investigator, the subject was withdrawn from the study and the study eligibility criteria were modified to exclude subjects with sinus bradycardia. No other serious or unanticipated adverse events attributed to the device or administration procedure have been observed.

The results of the completed HIV-1 ADVAX vaccine study in healthy subjects have been published (Vasan S *et al.* In vivo electroporation enhances the immunogenicity of an HIV-1 DNA vaccine candidate in healthy volunteers. PLoS One 2011;6(5):e19252). Briefly, results from this study indicate that EP-based delivery with the TDS-IM device at ADVAX DNA doses ranging from 0.2–4.0 mg was safe and effective in improving the magnitude, breadth and durability of cellular immune responses to a DNA vaccine candidate. Assessment of the tolerability of the EP procedure by questionnaire after each administration indicates that the procedure is generally acceptable for use in healthy subjects.

5.9.5 Preparation of the vaccine

Doses of the personalized polyepitope DNA vaccines will be prepared in the Siteman Cancer Center Investigational Pharmacy and delivered to the clinic on the day of vaccination. Vials will be thawed at room temperature on the day of study visits. After thawing the vaccine vial(s), the investigational pharmacist will mix the vial(s) completely by inverting the vial(s) at least 10 times (one inversion = one 180° turn of the wrist and back). The vaccine will then be withdrawn into 3.0 mL Becton Dickinson Model 309585 syringe under sterile conditions by the research pharmacist. For administration, a 22 gauge 1.5 inch injection needle will be affixed to the syringe. Once the dose is prepared, the research pharmacist will load the syringe into a TDS-IM Application Cartridge in a manner consistent with the instructions provided in the TDS-IM User’s Guide.

5.9.6 Vaccine administration

Detailed instructions for procedure administration using the TDS-IM device are included in the TDS-IM User’s Guide. Briefly, the TDS-IM Application Cartridge is loaded into the Integrated Applicator. The Pulse Stimulator is connected to an appropriate power source, turned on, and then connected to the Integrated Applicator through the supplied cable. Prior to administration, the skin at the site of administration is prepared according to the standard procedures for a conventional intramuscular injection. Once the skin has been disinfected, the skin around the injection site is held firmly while the Application Cartridge is placed against the injection site at a 90° angle. An indicator light on the device notifies the user that sufficient pressure has been applied. The activation button is then depressed, causing the electrodes and injection needle to be inserted into the target tissue. An automated safety check is performed, and, if passed, allows the injection of the study vaccine into the muscle. The procedure will conclude with a series of brief, localized muscle contractions at the administration site. The procedure will require approximately 7-10 seconds to complete, during which time the site is held firmly and with the device depressed against the skin. Once the indicator light on the device indicates that the procedure is complete, the device is withdrawn and site is covered with a sterile covering and pressure applied with 3 fingers for 1 minute.

5.10 Investigational agent accountability

5.10.1 Documentation

The investigational agent will be prepared by the study pharmacist at the Siteman Cancer Center Investigational Pharmacy. The study pharmacist will be responsible for maintaining an accurate record of the codes, inventory, and an accountability record of vaccine supplies for this study. Electronic documentation as well as paper copies will be used.

5.10.2 Disposition

The empty vials and the unused portion of a vial will be discarded in a biohazard containment bag and incinerated or autoclaved. Any unopened vials that remain at the end of the study will be returned to the production facility or discarded at the discretion of the principal investigator in accordance with policies that apply to investigational agents. Partially used vials will not be administered to other subjects or used for *in vitro* experimental studies. They will be disposed of in accordance with institutional or pharmacy policy.

6 NONCLINICAL STUDIES

6.1 Overview

We propose a phase 1 clinical trial of a personalized polyepitope DNA vaccine strategy. The personalized polyepitope DNA vaccine strategy is designed to target mutant tumor-specific antigens present in the cancer, but absent in normal tissues.

One of the reasons that we have pursued clinical development of a personalized polyepitope DNA vaccine strategy targeting mutant tumor-specific antigens is because we believe that this strategy has the potential to be safer than strategies targeting shared tumor antigens. Shared tumor antigens are typically expressed at high levels in the tumor, but are also typically expressed at lower levels in some normal tissues. Expression of shared tumor antigens in normal tissues may increase the risk of autoimmunity. Mutant tumor-specific antigens are present only in the tumor. In addition, our next-generation sequencing-based epitope prediction algorithm prioritizes epitopes where the mutant epitope (but not the wildtype epitope) can bind to restricting HLA molecules. This decreases the potential that immune responses targeting mutant tumor-specific antigens will be cross-reactive with wildtype antigens.

We do not think that GLP safety and toxicology studies will provide significant insight into the safety of the personalized polyepitope DNA vaccine strategy. First, it is impossible to know *a priori* what mutations will be present and/or prioritized in individual patients. We estimate that there are as many as 7 million potential mutant tumor-specific antigens that could be targeted by our approach. Only a limited number of mutations could be targeted in GLP safety and toxicology studies. Second, to our knowledge, no mammary tumor models exist that would be relevant for GLP safety and toxicology studies. Third, the pING parental vector has proven to be safe in phase 1 clinical trials.

We are not proposing to perform GLP biodistribution and integration studies at this stage in development. There is extensive GLP information available about the biodistribution and integration of DNA vaccines following electroporation with the TriGrid device. This includes GLP information about biodistribution and integration of DNA vaccines using the pING parent vector. Additional information about the biodistribution and integration of DNA vaccines following electroporation with the TriGrid device is summarized in the IND application.

6.2 Preclinical studies

It has long been known that there is a dynamic relationship between the immune system and cancer. This dynamic relationship has been studied in detail, ultimately resulting in the establishment of the cancer immunoediting concept [60-67].

We have recently focused on defining the antigens recognized by the immune system during the cancer immunoediting process. These studies, summarized below, demonstrate that mutant tumor-specific antigens are important tumor rejection antigens, and provide strong support for our personalized breast cancer vaccine strategy. Specifically, we have developed next-generation sequencing and epitope prediction algorithms to identify and prioritize mutant tumor-specific antigens. We will use these algorithms in the proposed clinical trial. The preclinical data supporting the use of these algorithms are presented below.

In initial studies we used a combination of next-generation sequencing and epitope prediction algorithms to identify mutant tumor-specific antigens in the d42m1 MCA sarcoma line. These algorithms identified one particular mutation (an R913L mutation of SPTBN2) as a top candidate, and subsequent analyses confirmed that this mutant tumor-specific antigen functioned as an immunodominant tumor rejection antigen. These studies were published in *Nature* [17].

The d42m1 MCA sarcoma is an unedited tumor, and would therefore be expected to express strong tumor rejection antigens. We have since turned our attention to examining the epitope landscape in edited MCA sarcomas that develop in immunocompetent wildtype mice. Specifically, we have asked the following questions: (1) Can the next-generation sequencing and epitope prediction algorithms be used more broadly to identify and prioritize important mutant tumor-specific antigens in less immunogenic

tumors? (2) Can the next-generation sequencing and epitope prediction algorithms be used to prioritize antigens for immune targeting and/or personalized vaccine therapy?

To address these questions we focused initial efforts on d42m1-T3. d42m1-T3 is a clone of d42m1 that lacks the immunodominant rejection antigen, mutant SPTBN2, and forms progressively growing tumors in wildtype mice. We specifically chose the d42m1-T3 clone because d42m1-T3 shares with naturally edited sarcomas the ability to form progressively growing tumors in wildtype mice and shows a similar sensitivity to checkpoint blockade.

To identify and prioritize mutant tumor specific antigens from the d42m1-T3 we used optimized next-generation sequencing and epitope prediction algorithms. Specifically, we pipelined the candidate mutant tumor-specific antigen sequences into four different MHC class I epitope prediction algorithms and calculated the median predicted affinity for binding to the relevant class I MHC alleles. We then applied filters that account for proteasomal processing of the antigen and differences in MHC class I binding affinity between mutant and native sequences to prioritize the mutant tumor-specific antigens. We also deprioritized hypothetical Riken proteins.

Of the top 61 prioritized candidates, 20 were eliminated by the filtering process; including two of the top four candidates. Of those that remained, two [G1254V Laminin subunit α4 (mLama4) and A506T alpha-1,3 glucosyltransferase (mAlg8)] were clearly favored above the others based on predicted binding affinity.

To test whether these two “best” mutant tumor-specific antigens were biologically relevant, we generated tumor-specific $CD8^+$ T cell lines from the spleens of three independent mice that had rejected d42m1-T3 cells after anti-PD-1 therapy and showed that each T cell line (CTL-62, CTL-73, CTL-74) displayed specificity for d42m1-T3 but not an unrelated sarcoma, F244. To determine if the “prioritized” mutant tumor-specific antigens were recognized by anti-d42m1-T3 T cell lines, we incubated 8 amino acid synthetic peptides corresponding to each of the top 61 initially predicted H-$2K^b$ mutant tumor-specific antigens with irradiated splenocytes and CTL-74 T cells and monitored IFNγ production. The mLama4 and mAlg8 peptides strongly stimulated CTL-74 T cells, with mLama4 inducing ~10x more IFNγ than mAlg8. No other predicted mutant epitope induced significant levels of IFNγ production in this assay. Similar results were obtained with the other two d42m1-T3 specific $CD8^+$ T cell lines. Subsequent dose response experiments showed that mLama4 stimulated the tumor-specific T cell lines to a greater extent than mAlg8 and that the T cells reacted specifically with mutant but not native peptides.

We then used four experimental systems to confirm that our optimized epitope prediction algorithms accurately prioritized mutant tumor-specific antigens. *First*, together with the groups of Hans-Georg Rammensee in Tübingen and Ruedi Abersold in Zurich we detected mLama4 and mAlg8 peptides bound to H-$2K^b$ on d42m1-T3 tumor cells. To our knowledge this is the first time that mutant class I epitopes have been detected bound to tumor cell-associated MHC class I. *Second*, using PE-labeled H-$2K^b$ tetramers carrying mLama4 or mAlg8 peptides, $CD8^+$ T cells with specificities for these two epitopes were found to accumulate in d42m1-T3 tumors in αPD-1 treated mice and reached peak values just prior to tumor rejection on day 12. Consistent with the results of the T cell stimulation experiments, mLama4-specific T cells were present in significantly higher numbers in the tumor than mAlg8-specific T cells. No mLama4- or mAlg8-specific T cells were observed in irrelevant, checkpoint blockade-sensitive F244 tumors. *Third*, vaccination of naïve WT mice with mutant-Lama4 or mutant-Alg8 short peptide vaccines (8mer) induced strong $CD8^+$ T cell responses that were specific for the mutant, but not the WT epitope (mLama4 = 1650 SFC/10^6 cells vs. wtLama4 = 75 SFC/10^6 cells; mAlg8 =606 SFC/10^6 cells vs. wtAlg8 = 50 SFC/10^6 cells). *Fourth*, prophylactic vaccination of mice with long peptides (~30mer) corresponding to either the mLama4 epitope alone, or both the mLama4 and mAlg8 epitopes induced protection against subsequent challenge with d42m1-T3 tumor cells. The combined peptide vaccine was more protective than the vaccine containing the mLama4 long peptide alone (Figure 5).

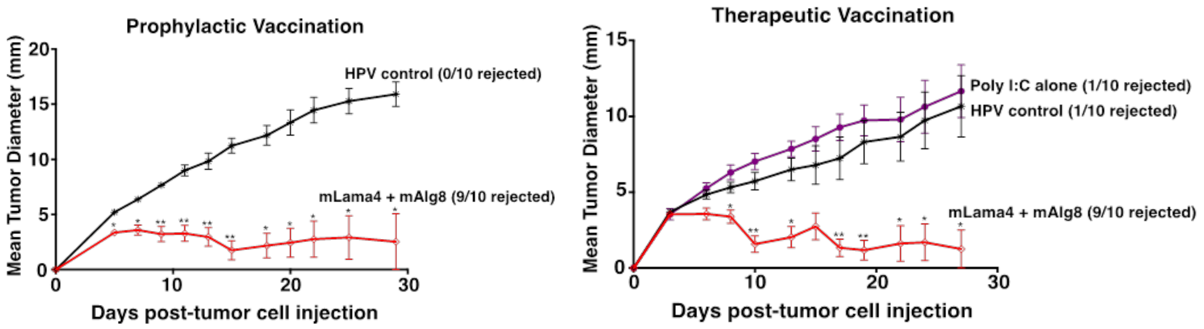

Figure 5. Synthetic long peptide vaccines for mLama4 and mAlg8 protect against tumor growth. (a) Groups of mice were prophylactically injected with synthetic long peptide vaccines (SLP) (plus the adjuvant Poly I:C) on days -10, -3 & +4 and challenged on day 0 with 10^6 d42m1-T3 sarcoma cells s.c. A SLP from HPV was used as a negative control. (b) Groups of mice were challenged on day 0 with 10^6 d42m1-T3 sarcoma cells s.c. and therapeutically injected with SLP vaccines (plus Poly I:C) on days +3, +9, & +15 post-tumor injection. Poly I:C alone or a SLP from HPV was used as a negative control. *p<0.05 **p<0.01.

Of note, immune responses to both mLama4 and mAlg8 were also observed after vaccination with a polyepitope DNA vaccine encoding mLama4, mAlg8, and several additional epitopes. Mice were vaccinated three times and the immune response to mLama4, mAlg8 and control peptides was assessed by ELISPOT using splenocytes from vaccinated mice. Vaccination with polyepitope DNA vaccines induced responses that were similar in magnitude as synthetic long peptide vaccines encoding mLama4 or mAlg8 (data not shown).

6.3 Rationale for no GLP safety studies

We do not think that GLP safety and toxicology studies will provide significant insight into the safety of the personalized polyepitope DNA vaccine strategy.

First, it is impossible to know *a priori* what mutations will be present and/or prioritized in patients. Next-generation sequencing of epithelial cancers has demonstrated that there are very few recurrent mutations present. Mutations can be present in any one of the approximately 20,000 protein-coding genes present in the human genome. Even more problematic is that the mutations targeted could be anywhere in the corresponding protein. It has been estimated that the average length of a protein in humans is 362 AA. Thus, there are potentially 7,240,000 potential mutant tumor-specific antigens that could be targeted by our approach. This includes only point mutations and does not include mutations resulting from indels. If studies are performed in a preclinical model, only a limited number of mutations will be targeted. For example if we design a personalized DNA vaccine specific for a murine epithelial cancer targeting 5 genes, this would represent only 5 of 7,240,000 potential mutant tumor-specific antigens. Even if 1000 GLP safety and toxicology studies were performed, each targeting 5 mutant tumor-specific antigens, it would still provide information on < 0.07% of potential mutant tumor-specific antigens.

Second, to our knowledge, no mammary tumor models exist that would be relevant for the proposed preclinical studies. In order to study mutant tumor-specific antigens, sequencing analyses of paired tumor/normal tissues are required. As such, we would need to study spontaneous tumors in mice, as tumors propagated as cell lines do not have corresponding normal DNA to evaluate. There are very few models of spontaneous tumor development in wildtype mice. Spontaneous tumors do develop in genetically engineered mice, but these oncogene-driven tumors in genetically engineered murine models of cancer typically have a very limited number of mutations. We have performed extensive studies in genetically engineered mice, and have found that there are very few mutations present in tumors derived from these mice. For example, we have performed extensive studies in the p53-null transplant mammary tumor model. p53 is a tumor suppressor gene and plays an important role in maintaining genome stability. As such one might expect that there would be a significant number of mutations present in p53-null transplant mammary tumors. However, we have sequenced > 10 p53-null transplant mammary tumors

and have found that there are only a limited number of mutations present in each tumor. This is not at all representative of human triple-negative breast cancers where significantly more mutations are present. Of note, a significant number of mutations must be present to reliably identify mutant tumor-specific antigens that are immunogenic, as many candidate mutant tumor-specific antigens are not processed and presented by the immune system, or cannot be recognized efficiently by T cells. If only a limited number of mutations is present, it is possible that no mutant tumor-specific antigens will be identified that are immunogenic. For meaningful GLP safety and toxicology studies of a personalized DNA cancer vaccine, one of the key considerations is to assess any potential toxicity associated with immune responses to the vaccine. If no mutant tumor-specific antigens are identified that are immunogenic, the GLP safety and toxicology studies will have only limited value as no immune response to the mutant tumor-specific antigen will be generated. As such, we are not aware of any mammary tumor models in mice that would be appropriate for the GLP safety and toxicology studies.

6.4 Rationale for no GLP biodistribution studies

We are not proposing to perform GLP biodistribution and integration studies at this stage in development. There is extensive GLP information available about the biodistribution and integration of DNA vaccines following electroporation with the TriGrid device. This includes GLP information about biodistribution and integration of DNA vaccines using the pING parent vector. The information available about the biodistribution and integration of DNA vaccines following electroporation with the TriGrid device is detailed below.

7 TREATMENT PLAN

7.1 Plasmid DNA administration

All subjects will be treated as outpatients in the Siteman Cancer Center.

The schedule of vaccination is Day 1, Day 29 ± 7, and Day 57 ± 7 with at least 21 days between injection days. All study injections will be given intramuscularly using an integrated electroporation device (TDS-IM system, Ichor Medical Systems). At each vaccination time point, patients will receive two injections of the personalized polyepitope DNA vaccine, one injection into each deltoid or lateralis. If both injections cannot be given on the same day, the patient will be asked to return to complete the second injection. Standard aseptic technique and precautions will be utilized in site preparation, vaccine administration, and medical waste disposal to ensure maximal safety of subjects and study personnel

The propagation of electroporation inducing electrical fields in the muscle will result in brief, localized muscle contractions at the site of administration, which are transiently painful. The personalized polyepitope DNA vaccines will be administered by an experienced nurse who has completed a training seminar on the use of the TDS-IM device.

The sites of immunization may be rotated for each of the immunizations. No injection will be given at a location in which the draining lymph nodes have been removed.

At the discretion of the treating physician, Patients may be pre-medicated with lorazepam 1mg PO (or similar) at least 30 minutes but no greater than 60 minutes prior to the first injection. Patients may also receive a second dose of lorazepam (1 mg PO) (or similar) 10 minutes prior to injection. Patients may also receive acetaminophen 650mg PO at least 30 minutes but no greater than 60 minutes prior to the first injection.

Following study injections, subjects will be observed for a minimum of 30 minutes. Vital signs (temperature, blood pressure, pulse and respiratory rate) will be taken at 30-45 minutes post-immunization. The injection sites will be inspected for evidence of local reaction. Subjects will be given a "Diary Card" on which to record temperature and symptoms daily for 5 days. The "Diary Card" will contain contact information for the study team should they have any questions or concerns.

On each injection day (prior to injection) study subjects will be evaluated by clinical exam and laboratory tests. At 14 ± 3 days after each injection, study subjects will be required to return for a follow-up visit or be seen by their local oncologist for clinical exam and laboratory tests. Long-term follow-up visits are at Week 11 ± 7 days and Week 24 ± 14 days. Additional follow-up visits or telephone contact will be scheduled at Week 52 and annually thereafter if the patient is alive and available for follow-up.

At intervals throughout the study (both before and after vaccination) subjects will have blood drawn for immunologic assays. Any cells, serum or plasma not used will be stored for future immunological assays.

Please see **Section 9 STUDY CALENDAR** for details on study visit procedures and monitoring.

7.2 Duration of therapy

In the absence of treatment delays due to adverse events, treatment may continue for 3 doses of personalized polyepitope DNA breast cancer vaccines. Under certain circumstances, a subject will be terminated from participating in further injections. Subjects who are discontinued from additional study injections will continue to be followed according to the schedule of safety and immunogenicity evaluations. Please see **Section 11 REMOVAL OF PATIENTS FROM PROTOCOL THERAPY** for additional details.

7.3 Duration of follow up

Patients will be followed for 52 weeks or until death, whichever occurs first. Additional follow-up visits or telephone contact will be scheduled annually thereafter if the patient is alive and available for follow-up. Patients removed from study for unacceptable adverse events will be followed until resolution or stabilization of the adverse event.

8 POTENTIAL TOXICITY AND DOSE MODIFICATIONS

8.1 Previous human experience

8.1.1 Experience with the pING parent vector

The personalized polyepitope DNA vaccines have not been used in humans to date. Conventional DNA cancer vaccines based on the pING parent vector have been used extensively in phase 1 human clinical trials.

We have discussed this issue with Dr. Robert Jambou at the Office of Biotechnology Activities, National Institutes of Health. On January 12, 2009, We made a Freedom of Information Act (FOIA) request for copies of information on the study population, dosing regimen, and study results to include a detailed adverse events/safety information for six NIH OBA-registered clinical trials: 0005-394, 0105-474, 0105-474, 0303-573, 0312-617, 0412-684 and 0412-685. Dr. Jambou sent us the relevant clinical protocols, Appendix M documents, safety reports and annual reports.

In accordance with Appendix M-I-C-4 of the NIH Guidelines for Research Involving Recombinant DNA Molecules posted April 1, 2002 in the Federal Register, investigators who have received authorization from the FDA to initiate a human gene transfer protocol must report in writing any unanticipated problems (serious adverse events or SAEs) to the NIH Office of Biotechnology Activities (OBA). These guidelines state that "Principal Investigators must submit, in accordance with Appendix M-I-C-4, a written report on any serious adverse event that is both unexpected and associated with the use of the gene transfer product. Any serious adverse event that is fatal or life-threatening, that is unexpected, and associated with the use of the gene transfer product must be reported to the NIH OBA as soon as possible, but not later 24 hours after the sponsor’s initial receipt of the information. Serious adverse events that are unexpected and associated with the use of the gene transfer product, but are not fatal or life-threatening, must be reported to the NIH OBA as soon as possible, but not later than 15 calendar days after the sponsor’s initial receipt of the information."

We have used this information to prepare Table 4 below. Of note, there was only one serious adverse event reported. One patient with metastatic prostate cancer developed urinary retention. It is not clear if this was related to the DNA vaccine. Review of the available annual reports confirms minimal toxicity associated with DNA vaccination in these trials. Over 90% of the toxicities related to treatment were grade 1 and included diarrhea, dizziness, edema, fatigue, injection site reaction, nausea, vomiting, and rigors.

We have also discussed these trials with Jedd Wolchock, M.D., at Memorial Sloan-Kettering Cancer Center, by e-mail correspondence January 15, 2009. Dr. Wolchock is the PI or co-investigator on all of these trials. He confirmed that there has been minimal toxicity associated with the DNA vaccines based on the pING parent vector.

Table 4 Clinical Experience with pING parent vector

Protocol number	Study population	Dosing regimen	Route of administration	Number and frequency	Safety
0005-394	Metastatic melanoma, 18 patients total	100, 500, or 1500 ug DNA	Intramuscularly using Biojector 2000	three vaccinations three weeks apart	No SAEs reported
0105-474	Prostate cancer, 36 patients	100, 500, or 1500 ug DNA	Intramuscularly using Biojector 2000	six vaccinations three weeks apart	One SAE reported, deemed to be unrelated to the vaccine
0303-573	Metastatic melanoma, 18 patients total	100, 500, or 1500 ug DNA	Intramuscularly using Biojector 2000	six vaccinations three weeks apart	No SAEs reported
0312-617	Renal Cell Carcinoma, 18 patients	500, 1500, or 3000 ug DNA	Intramuscularly using Biojector 2000	six vaccinations three weeks apart	No SAEs reported
0412-684	Breast cancer, 12 patients	500, 1000, 3000, or 6000 ug DNA	Intramuscularly using Biojector 2000	five vaccinations three weeks apart	No SAEs reported
0412-685	Metastatic melanoma, 18 patients	500, 2000, or 4000 ug DNA	Intramuscularly using Biojector 2000	six vaccinations three weeks apart	No SAEs reported

8.2 Potential toxicity

8.2.1 Potential toxicity related to the personalized polyepitope DNA vaccine

The primary objective of this trial is to evaluate the safety of the personalized polyepitope DNA vaccines. This is the one of the first times that mutant tumor-specific antigens identified by next generation sequencing have been targeted for immune therapy in humans. This is also the first time that personalized polyepitope DNA vaccines have been administered to humans. However, clinical trials of similar investigational plasmid DNA vaccines suggest that these vaccines will be very safe. We expect that most of the toxicity to be limited to local grade 1 or 2 reactions at the vaccination site.

Please note that the risks detailed below are based on the risks of injections, the risks of vaccines in general, and the results of previous studies with investigational DNA vaccines.

Risks associated with intramuscular administration of a DNA plasmid include acute bleeding and/or bruising. Although highly unlikely, intramuscular injection can result in muscle damage, peripheral nerve damage, and/or injection site infection. Due to the insertion and activation of multiple electrodes, use of the TDS-IM electroporation device may increase these risks. The propagation of electroporation inducing electrical fields in the muscle will result in brief, localized muscle contractions at the site of administration, which are transiently painful. As with any immunization, discomfort or redness at the injection site in the days following DNA vaccine administration may be expected. Since intramuscular DNA delivery with electroporation results in increased intracellular uptake of plasmid at the site of injection and electric field application, the procedure may increase the frequency and/or severity of local site reactions compared to conventional intramuscular administration of DNA vaccines. Such symptoms should not last longer than several days.

Study subjects can receive medications such as acetaminophen, NSAIDs, or antihistamines as required. Steroids will not routinely be used in study subjects; if steroids are required the study subject will receive no further immunizations, but will continue to be monitored in follow-up visits.

Subjects may exhibit general signs and symptoms associated with administration of a vaccine injection, including fever, chills, rash, aches and pains, nausea, headache, dizziness and fatigue. These side effects will be monitored, but are generally short term and do not require treatment.

The possibility of integration of the DNA plasmid vector into genomic DNA of transfected myocytes has been considered. Plasmid integration at a sufficiently high frequency carries the possibility of inducing deleterious mutations. Potential side effects could include an increased risk of malignancy arising from the cells harboring the mutation(s). However, current evidence from laboratory and animal studies indicates that the frequency of induced mutations following electroporation of DNA is conservatively estimated to be two to three orders of magnitude lower than that of naturally-occurring gene inactivating mutations in healthy humans.

The effect of this vaccine on a fetus or nursing baby is unknown, so female subjects of child bearing potential will be required to agree to use birth control for sexual intercourse beginning 21 days prior to enrollment and continuing through the last protocol visit. Women who are pregnant or nursing will be excluded from the study.

The potential discomforts of this study include having blood drawn, intramuscular injection of the vaccine, and possible reactions to the vaccine. Drawing blood causes transient discomfort and may cause fainting. Bruising at the blood draw site may occur, but can be prevented or lessened by applying pressure for several minutes. Intramuscular injection also causes transient discomfort. Infection at the site of blood drawing or vaccination is extremely unlikely as alcohol swabbing and sterile equipment will be used.

The use of plasmid DNA has the potential to cause an allergic reaction due to the presence of bacterial endotoxin. However, each lot of DNA will be tested for endotoxin to ensure that endotoxin content does not exceed USP specifications. Antibodies to DNA may potentially develop in DNA vaccine recipients. However, the development of such antibodies in response to DNA vaccination is rare and has not been associated with disease in animals or humans to date.

8.3 Toxicity monitoring and management

Toxicity will be characterized according to the National Cancer Institute Common Terminology Criteria for Adverse Events version 4.0 (CTCAE). Subjects who are immunized with the plasmid DNA vaccine will be evaluated at the time of each vaccination on Day 1, Day 29 ± 7, and 57 ± 7. Follow up on subject well-being will be performed by telephone on the first or second day after each vaccination. Subjects will maintain a study diary to note any symptoms related to vaccination. All information will be recorded on case report forms. Adverse events will be reported to the Quality Assurance and Safety Monitoring Committee of the Siteman Cancer Center, the Institutional Review Board, the Institutional Biosafety Committee, the Office of Biotechnology Activities and the Food and Drug Administration as detailed in **Section 12 ADVERSE EVENT REPORTING**.

Significant local inflammation will be treated with cold packs and oral analgesics as indicated. Skin ulceration at the vaccine site will be treated with local wound care and antibiotics as indicated. Autoimmune involvement of the breast will be treated conservatively with analgesics; more aggressive intervention (systemic corticosteroids) will be used as necessary

8.4 Dose modifications

No dose modifications are planned. If a subject develops an adverse event that is classified as possibly, probably, or definitely associated with protocol therapy, this may result in removal of the subject from protocol therapy as outlined in **Section 11 REMOVAL OF SUBJECTS FROM PROTOCOL THERAPY**. Protocol Stopping Criteria are outlined in **Section 13 DATA AND SAFETY MONITORING**.

9 STUDY CALENDAR

	Pre-Study[g]	Wk 1	Wk 2	Wk 3[e]	Wk 4	Wk 5	Wk 6	Wk 7[e]	Wk 8	Wk 9	Wk 10	Wk 11[e]	Wk 24	Wk 52[h]
Plasmid DNA vaccine[a]		X				X				X				
Informed consent	X													
Demographics	X													
Medical history	X													
Concurrent meds	X	X---X												
Physical exam	X	X		X		X		X		X		X	X	
Vital signs	X	X		X		X		X		X		X	X	
Height	X													
Weight	X	X		X		X		X		X		X	X	
Performance Status	X	X				X				X				
CBC w/diff, plts[k]	X			X		X		X		X		X	X	
CMP[b, k]	X			X		X		X		X		X	X	
EKG (as indicated)	X													
Adverse event evaluation	X	X[c]		X		X[c]		X		X[c]		X	X	X
Tumor measurements	Tumor measurements will be performed as clinically indicated. Documentation (radiologic) must be provided for patients removed from study for progressive disease.													
Radiologic evaluation	Radiologic measurements will be performed as clinically indicated.													
B-HCG (as indicated)	X[d]					X[d]				X[d]			X[d]	
Immune monitoring[j]	X	X		X[i]		X				X		X[i]	X	X
Tumor specimen[f]														

a: Personalized polyepitope breast cancer DNA vaccine

b: Albumin, alkaline phosphatase, total bilirubin, bicarbonate, BUN, calcium, chloride, creatinine, glucose, potassium, total protein, SGOT [AST], SGPT [ALT], sodium.

c: Adverse event evaluation on day of vaccine and by telephone contact on the first or second day after vaccine. Follow-up or telephone contact at week 52 and annually thereafter if the patient is alive and available for follow-up.

d: Serum or urine pregnancy test (women of childbearing potential).

e: ± 3 days; assessments may take place by return office visit to SCC or by local oncologist.

f: At time of port placement if fresh biopsy will be performed, and again at time of surgery.

g: Pre-study assessments shall be repeated no more than 28 days prior to the first vaccine injection (with the exception of immune monitoring, CBC, and CMP)

h: ± 14 days

i: Optional for non-local patients who are not returning to SCC at this time point.

j: The blood draw for immune monitoring will be done once prior to the first vaccine injection. This can be done at any time after the patient signs consent but will occur before the first vaccine. Blood will be drawn for immune monitoring per the study calendar, irrespective of the vaccine administration.

k: Pre-study assessments shall be repeated no more than 14 days prior to the first vaccine injection.

10 CRITERIA FOR RESPONSE

10.1 Primary objective: safety

Assessment of plasmid DNA safety will include both clinical observation and laboratory evaluation. Safety will be closely monitored after injection with eight or more clinical and laboratory assessments in the first 24 weeks of the trial. The following parameters will be assessed following vaccination:

(1) Local signs and symptoms

(2) Systemic signs and symptoms

(3) Laboratory evaluations, including blood counts and serum chemistries

(4) Adverse, and serious adverse events

Toxicity will be graded according to the National Cancer Institute Common Terminology Criteria for Adverse Events v4.0.

10.2 Secondary objectives: evaluation of the immune response

10.2.1 Introduction to immune monitoring

The secondary endpoint is to evaluate the immunogenicity of the personalized polyepitope DNA vaccine. Immunogenicity will be measured by ELISPOT analysis, a surrogate for CD8 T cell function, and multiparametric flow cytometry. In both assays the quantity and quality of antigen-specific CD8 T cells is determined; the ELISPOT analysis is based on measuring the frequencies of IFN-γ producing T cells in response to polyepitope antigen, whereas the multiparametric flow cytometry assesses phenotypic as well as functional characteristics of epitope-specific CD8 T cells. In the proposed study, blood samples will be collected at multiple time points (n=8) and PBMC isolated and cryopreserved. Upon completion of the vaccination protocol, all samples will be analyzed simultaneously in order to minimize assay-to-assay variation.

10.2.2 ELISPOT Analysis

ELISPOT analysis, will be performed as previously described [68-72]. PBMC from subjects in the phase 1b clinical trial will be tested for secretion of IFN-γ by ELISPOT assay. PBMC will be plated at various concentrations starting at 300,000 cells per well, in triplicate, following the protocol previously described by Dr. Mohanakumar and colleagues for detection of breast cancer-specific T cells. PBMC will be co-cultured with the mutant peptides encoded by the polyepitope construct. As negative controls, PBMC will be incubated in medium alone or stimulated with matching wild type peptides. As a positive control we will include a mix of viral peptides, CEF, containing immunodominant epitopes for multiple common HLA alleles from influenza virus, cytomegalovirus, and Epstein-Barr virus. After 24-48 hours, the plates will be developed and the spots counted in an ImmunoSpot Series I analyzer (Cellular Technology).

10.2.3 Multi-parametric flow cytometry

Patient-derived PBMC will be used for functional assays to characterize immunity to personalized breast cancer DNA vaccines. Polyfunctional $CD8^+$ T cell responses will be determined after stimulation with polyepitope pulsed autologous PBMC using muti-parameter flow cytometry. Fluorescently labeled MHC class I tetramers expressing vaccine-encoded peptides will be used to gate on peptide-specific T cells.

PBMC aliquots ($2x10^6$ cells/mL) will be stimulated with individual peptides encoded by the polyepitope vaccine and cultured in the presence of IL-2 (50U/mL). Ten days after activation, cells will be harvested, washed and restimulated with irradiated (10,000 rads) peptide-pulsed autologous PBMC for 16h (in the presence of Brefeldin A) and stained with anti-CD8 (FITC), anti-IFN-γ (PE), anti-TNF-α (PerpCy5.5), anti-IL-2 (APC) and CD107a (APC-Cy7), a marker for cytolytic activity. Cells will be analyzed by the Immune Monitoring Core, also known as the Center for Human Immunology and Immunotherapy Programs (CHiiPs), headed by Dr. Robert Schreiber. Cultured cells stimulated with unpulsed PBMC will be used as negative control, and cells stimulated with CEF-pulsed PBMC will be used as positive controls using an appropriate tetramer. One advantage of using 10 day activated/antigen-driven PBMC to measure

polyfunctional responses is the expected relative high frequency of polyepitope-specific T cells providing a larger sample size for statistically significant data analysis.

10.3 Exploratory objectives

Additional assays of T cell immunity will include cytotoxicity assays using a flow cytometry-based killing assay (FloKA). Patient-derived PBMC will be assessed for their ability to lyse cells expressing epitopes encoded by the polyepitope vaccine.

Clinical responses and time to disease progression will be evaluated with physical examination and diagnostic imaging as clinically indicated.

10.3.1 Correlative studies

Although the ELISPOT assay is widely accepted as a predictive measurement of vaccination success or failure [73, 74], this assay provides minimal information as to the functional characteristics of vaccine-induced T cells. Correlative studies are planned in this study with the goal of discovering potential biomarkers for better prediction of clinical outcome. Additionally, these correlative studies may allow investigators to better understand the nature of immune responses specific for a patient's unique tumor protein variants. We plan to obtain blood samples prior to vaccination, during, and post-vaccination (see section 9) in order to procure PBMC for correlative studies. Functional studies will include ELISPOT, T cell poly-functionality by intra-cellular cytokine and degranulation analysis using multi-parametric flow cytometry, and cytotoxicity assays.

10.3.2 Sample collection plan

After trial enrollment, patients will undergo their first blood draw at baseline according to standard procedures. Additional blood samples will be obtained at Weeks 1, 3 (optional for non-local patients), 5, 9, 11 (optional for non-local patients), 24, and 52. Ten mL BD Vacutainer® sodium heparin (green top) tubes will be used for collection (REF 367874). At baseline and at Weeks 1, 11, and 24, twelve tubes (approximately 120 mL) will be collected. At Weeks 3, 5, 9, and 52, six tubes (approximately 60 mL) will be collected. Blood samples will be transported to Dr. Gillanders' laboratory (6th floor Clinical Sciences Research Building, room 6664) within one hour of collection. PBMC will be obtained by Ficoll-Hypaque gradient centrifugation and cryopreserved in 10% DMSO according to standard procedures.

Exome sequencing of PBMC will be performed to obtain germline sequences.

In addition to blood samples, tumor samples will be collected during surgical resection post-neoadjuvant chemotherapy and will be prioritized for tumor exome and tumor cDNA-capture sequencing. Alternatively, specimens from biopsies performed at the time of initial diagnosis will be used, if available. Tissue will be placed in complete RPMI or equivalent media solution on ice and transported to Dr. Gillanders' lab. Tumor tissue will be macro-dissected from grossly normal breast tissue upon receipt by the lab. When tissue amount appears limited, priority will be given to: 1) embedding in optimal cutting temperature (OCT) compound and 2) preparing a single cell suspension.

<u>OCT embedding</u>: approximately 10-50 mg of tissue, sectioned into pieces no larger than 0.5 cm x 0.5 cm, will be prepared. Cryomold will be filled slowly to the top with OCT compound. Tissue will be gently submerged into the OCT compound in the cryomold. OCT will be hardened by cooling. This will be achieved by placing the cryomold in the vapor phase of liquid nitrogen or on dry ice. After the OCT has hardened, the mold will be placed in a container and transferred to a -80°C freezer for storage. The frozen tissue will be transferred to the Laboratory of Translational Pathology (LTP, BJC-Institute of Health, Room 5110) for sectioning, H&E staining, and pathology evaluation.

<u>Single cell suspension</u>: the remaining tumor sample will undergo mechanical and enzymatic digestion and be cryopreserved for further downstream studies such as multi-parametric flow cytometry.

<u>Nucleic acid isolation</u>: DNA will be isolated from PBMC by the LTP or Gillanders laboratory personnel for exome sequencing at The Genome Institute. To identify somatic mutations, DNA and RNA will be extracted from OCT-embedded tissue. The OCT block will be delivered to the LTP where the block will be sectioned and stained in order to confirm the presence of tumor, determine tumor/normal ratio, and guide isolation of tumor cells by, for instance, laser capture microdissection (LCM). All tissue selected for

sequencing will be processed into a single-cell suspension by mechanical and enzymatic digestion, and used to extract nucleic acids. Tumor DNA + RNA will then undergo tumor exome and tumor cDNA-capture sequencing, respectively at The Genome Institute.

10.3.3 Cytotoxicity assay

The granule-exocytosis pathway is a primary effector immune mechanism that promotes tumor cell death. PBMC cultured as described above will be tested in cytotoxicity assays for their ability to recognize (1) the antigen-presentation deficient cell line, T2, pulsed with peptide encoded by the personalized vaccine, and (2) cells isolated from patient-derived xenograft. While the T2 cell line is homozygous for HLA-A2, we have generated multiple genetic variants of T2 that express other common HLA class I alleles.

The appropriate T2 line will be labeled with the fluorescent dye, CFSE, irradiated, and pulsed with peptide. Cultured cells will be harvested at around day 12 and cocultured with peptide-pulsed T2 at a T cell to T2 ratio of 20:1 for 4-6 hr. All cells will be harvested and the viability dye, 7-amino-actinomycin D (7-AAD) will be added, followed by flow cytometry analysis. Peptide-pulsed T2 cultured for 4-6 hr in the absence of T cells will be analyzed to assess background levels of killing. The percentage killing will be assessed by gating on CFSE-positive cells, and calculating the percentage 7-AAD-positive cells over spontaneous lysis.

11 REMOVAL OF SUBJECTS FROM PROTOCOL THERAPY

11.1 Removal of subjects from protocol therapy

Subjects may be removed from protocol therapy if any one or more of the following events occur:

(1) Development of progressive or recurrent disease requiring systemic treatment or radiation therapy;

(2) Intercurrent illness that prevents further administration of protocol therapy;

(3) Pregnancy;

(4) Type 1 hypersensitivity reaction associated with protocol therapy;

(5) Grade 2 systemic or injection site adverse event classified as possibly, probably, or definitely associated with protocol therapy that does not resolve to at least grade 1 prior to the next scheduled treatment;

(6) Grade 3 or 4 systemic or injection site adverse event classified as possibly, probably, or definitely associated with protocol therapy;

(7) Any significant autoimmune disease or phenomena presumed to be related to protocol therapy;

(8) Subject refusal to continue protocol therapy and/or observations;

(9) Significant protocol violation or noncompliance, either on the part of the subject or investigator(s);

(10) The principal investigator or study sponsor believes it is in the subject's best interest to discontinue participation in the study;

(11) Administrative reasons, e.g., study termination by the principal investigator, Siteman Cancer Center, HRPO, FDA, or other group.

Please note that even if a subject is removed from protocol therapy, they will continue to be followed for adverse events.

11.2 Voluntary subject withdrawal

The subject has the right to voluntarily withdraw from the study at any time for any reason without prejudice to her future medical care by the physician or at the institution.

For any subject who withdraws consent, the date and reason for consent withdrawal should be documented. Subject data will be included in the analysis up to the date of the consent withdrawal.

11.3 Procedure for discontinuation

The procedure to be followed at the time a subject either discontinues participation or is removed from the study is:

(1) Check for the development of adverse events.

(2) Complete the End-of-Study form and include an explanation of why the subject is withdrawing or withdrawn.

(3) Attempt to perform follow-up evaluations as outlined above.

12 ADVERSE EVENT REPORTING

The entities providing oversight of safety and compliance with the protocol require reporting as outline below.

The Washington University Human Research Protection Office (HRPO) requires that all events meeting the definition of unanticipated problem or serious noncompliance be reported as outlined in Section 12.2.

The FDA requires that all serious and unexpected adverse events be reported as outlined in Section 12.3. In addition, any fatal or life-threatening adverse experiences where there is a reasonable possibility of relationship to study intervention must be reported.

12.1 Definitions

12.1.1 Adverse Events (AEs)

Definition: any unfavorable medical occurrence in a human subject including any abnormal sign, symptom, or disease.

Grading: the descriptions and grading scales found in the revised NCI Common Terminology Criteria for Adverse Events (CTCAE) version 4.0 will be utilized for all toxicity reporting. A copy of the CTCAE version 4.0 can be downloaded from the CTEP website.

Attribution (relatedness), Expectedness, and Seriousness: the definitions for the terms listed that should be used are those provided by the Department of Health and Human Services' Office for Human Research Protections (OHRP). A copy of this guidance can be found on OHRP's website: http://www.hhs.gov/ohrp/policy/advevntguid.html

12.1.2 Serious Adverse Event (SAE)

Definition: any adverse drug experience occurring at any dose that results in any of the following outcomes:

- Death
- A life-threatening adverse drug experience
- Inpatient hospitalization or prolongation of existing hospitalization
- A persistent or significant disability/incapacity (i.e., a substantial disruption of a person's ability to conduct normal life functions)
- A congenital anomaly/birth defect
- Any other experience which, based upon appropriate medical judgment, may jeopardize the subject and may require medical or surgical intervention to prevent one of the outcomes listed above

All unexpected SAEs must be reported to the FDA.

12.1.3 Unexpected Adverse Experience

Definition: any adverse drug experience, the specificity or severity of which is not consistent with the current investigator brochure (or risk information, if an IB is not required or available).

Events that are both serious AND unexpected must be reported to the FDA.

12.1.4 Life-Threatening Adverse Experience

Definition: any adverse drug experience that places the subject (in the view of the investigator) at immediate risk of death from the reaction as it occurred, i.e., it does not include a reaction that, had it occurred in a more severe form, might have caused death.

Life-threatening adverse experiences must be reported to the FDA.

12.1.5 Unanticipated problems

Definition:

- unexpected (in terms of nature, severity, or frequency) given (a) the research procedures that are described in the protocol-related documents, such as the IRB-approved research protocol and informed consent document; and (b) the characteristics of the subject population being studied;
- related or possibly related to participation in the research ("possibly related" means there is a reasonable possibility that the incident, experience, or outcome may have been caused by the procedures involved in the research); and
- suggests that the research places subjects or others at a greater risk of harm (including physical, psychological, economic, or social harm) than was previously known or recognized.

12.1.6 Noncompliance

Definition: failure to follow any applicable regulation or institutional policies that govern human subjects research or failure to follow the determinations of the IRB. Noncompliance may occur due to lack of knowledge or due to deliberate choice to ignore regulations, institutional policies, or determinations of the IRB.

12.1.7 Serious noncompliance

Definition: noncompliance that materially increases risks, that results in substantial harm to subjects or others, or that materially compromises the rights or welfare of participants.

12.1.8 Protocol exceptions

Definition: A planned deviation from the approved protocol that are under the research team's control. Exceptions apply only to a single participant or a singular situation.

Pre-approval of all protocol exceptions must be obtained prior to the event.

12.2 Reporting to the Human Research Protection Office (HRPO) at WUSM

The PI is required to promptly notify the IRB of the following events:

- Any unanticipated problems involving risks to participants or others which occur at WU, any BJH or SLCH institution, or that impacts participants or the conduct of the study.
- Noncompliance with federal regulations or the requirements or determinations of the IRB.
- Receipt of new information that may impact the willingness of participants to participate or continue participation in the research study.

These events must be reported to the IRB within **10 working days** of the occurrence of the event or notification to the PI of the event. The death of a research participant that qualifies as a reportable event should be reported within **1 working day** of the occurrence of the event or notification to the PI of the event.

12.3 Reporting to the Quality Assurance and Safety Monitoring Committee (QASMC) at Washington University

The PI is required to notify the QASMC of any unanticipated problem occurring at WU or any BJH or SLCH institution that has been reported to and acknowledged by HRPO as reportable. (Unanticipated problems reported to HRPO and withdrawn during the review process need not be reported to QASMC.)

QASMC must be notified within **10 days** of receipt of IRB acknowledgment via email to a QASMC auditor.

12.4 Reporting to the Institutional Biosafety Committee

In accordance with institutional policies and NIH guidelines, any unanticipated problems must be reported to the Institutional Biological and Chemical Safety Committee (IBC) at Washington University School of Medicine. When submitting reports to the IBC, the FDA MedWatch form will be used. Please send completed reports to:

WUSM IBC
Attn: Susan Cook
660 S. Euclid Ave., CB 8229
St. Louis, MO 63110
Tel: (314) 747-0309
Email: shcook@wustl.edu

WUSM IBC guidelines specify that a copy of all unanticipated problems must be submitted to the IBC for review and documentation simultaneously with IRB submission. Additionally, any study modifications related to the investigational agent as well as the IRB renewal paperwork should be sent to the IBC for approval. The IRB and IBC will review all submissions simultaneously.

12.5 Reporting to the FDA

The conduct of the study will comply with all FDA safety reporting requirements according to 21 CFR 312.32. **PLEASE NOTE THAT REPORTING REQUIREMENTS FOR THE FDA DIFFER FROM REPORTING REQUIREMENTS FOR HRPO/QASMC.** It is the responsibility of the investigator to report any unanticipated problem to the FDA as follows:

- Report any unexpected fatal or life-threatening adverse experiences (12.1.4) associated with use of the drug (i.e., there is a reasonable possibility that the experience may have been caused by the drug) by telephone or fax no later than **7 calendar days** after initial receipt of the information.

Report any serious, unexpected adverse experiences (12.1.3), as well as results from animal studies that suggest significant clinical risk within **15 calendar days** after initial receipt of this information. A paper and a digital copy of each MedWatch form and accompanying FDA Form 1571 will be sent by the investigator or investigator's team to the FDA at the following address by FedEx or other expedited mail courier service:

Food and Drug Administration
Center for Biologics Evaluation and Research
Document Control Center
10903 New Hampshire Ave.
WO71-G112
Silver Spring, MD 20993-002
Attn : Erica Giordano

12.6 Reporting to the NIH Office of Biotechnology Activities (OBA)

Principal investigators must submit a written report on any adverse event that is both unexpected and associated with the use of the gene transfer product (i.e., there is reasonable possibility that the event may have been caused by the use of the product; investigators should not await definitive proof of

association before reporting such events). The report must be clearly labeled as a "Safety Report" and must be submitted to the NIH OBA and the local IBC within the time frame specified below.

Any serious adverse event that is fatal or life-threatening, that is unexpected, and that is associated with the use of the gene transfer product must be reported to the NIH OBA as soon as possible, but not later than 7 calendar days after the PI's initial receipt of information (i.e., at the same time the event must be reported to the FDA).

Serious adverse events that are unexpected and associated with the use of the gene transfer product, but are not fatal or life-threatening, must be reported to NIH OBA as soon as possible, but not later than 15 calendar days after the PI's initial receipt of information (i.e., at the same time the event must be reported to the FDA).

If, after further evaluation, an adverse event initially considered not to be associated with the use of the gene transfer product is subsequently determined to be associated, then the event must be reported to the NIH OBA within 15 days of the determination.

Relevant additional clinical and laboratory data may become available following the initial serious adverse event report. Any follow-up information relevant to a serious adverse event must be reported within 15 calendar days of the PI's receipt of the information. If a serious adverse event occurs after the end of the clinical trial and is determined to be associated with the use of the gene transfer product, that event shall be reported to the NIH OBA within 15 calendar days of the determination. The written report may be submitted on the FDA's MedWatch form. Submit to OBA by emailing oba@od.nih.gov or by fax at (301) 496-9839.

12.7 Reporting to Ichor Medical Systems

Since the TDS-IM device may be involved with the occurrence of adverse events, Ichor Medical Systems will be included in the Adverse Event reporting plan. Reports to Ichor for SAEs or for adverse events with possible relationship to the device should follow the timing for reporting to the WUSM Human Research Protection Office and should be directed to Drew Hannaman at dhannaman@ichorms.com.

12.8 Timeframe for reporting required events

Reportable adverse events will be tracked for 30 days following the last day of study treatment.

13 DATA AND SAFETY MONITORING

13.1 Protocol stopping criteria

The principal investigator will closely monitor and analyze study data as they become available and will make determinations regarding the presence and severity of adverse events. The administration of study injections and new enrollments will be halted and the QASMC promptly notified if any of the following events occurs:

(1) **One** (or more) subject(s) experiences a Grade 3 or 4 adverse event that is classified as probably or definitely related to vaccination;

(2) **One** (or more) subject(s) experiences a vaccine-related SAE;

(3) **Two** (or more) subjects experience the **same** Grade 2 or higher adverse event that is classified as probably or definitely related to vaccination: this criterion applies to fever, vomiting, laboratory abnormalities or other clinical adverse experiences, but does not apply to the subjective local or systemic symptoms of pain/tenderness, malaise, fatigue, headache, chills, nausea, myalgia, or arthralgia.

(4) Any other observation occurs that in the opinion of the PI results in a recommendation to halt enrollment.

If one of these events does occur, study injections and study enrollments would only resume if review of the adverse events that caused the halt resulted in a recommendation to permit further study injections and study enrollments.

The QASMC, in consultation with the principal investigator, will conduct any review and make the decision to resume or close the study for any Grade 2 or 3 events leading to a halt in the study.

The QASMC, with participation by the principal investigator, will consult with the FDA to conduct the review and make the decision to resume or close the study for all Grade 4 adverse events leading to a halt in the study.

13.2 Data safety monitoring plan

In compliance with the Washington University Institutional Data and Safety Monitoring Plan, the Principal Investigator will provide a Data and Safety Monitoring (DSM) report to the Washington University Quality Assurance and Safety Monitoring Committee (QASMC) semi-annually beginning six months after accrual has opened (if at least five patients have been enrolled) or one year after accrual has opened (if fewer than five patients have been enrolled at the six-month mark).

The Principal Investigator will review all patient data at least every six months, and provide a semi-annual report to the QASMC. This report will include:

- HRPO protocol number, protocol title, Principal Investigator name, data coordinator name, regulatory coordinator name, and statistician
- Date of initial HRPO approval, date of most recent consent HRPO approval/revision, date of HRPO expiration, date of most recent QA audit, study status, and phase of study
- History of study including summary of substantive amendments; summary of accrual suspensions including start/stop dates and reason; and summary of protocol exceptions, error, or breach of confidentiality including start/stop dates and reason
- Study-wide target accrual and study-wide actual accrual
- Protocol activation date
- Average rate of accrual observed in year 1, year 2, and subsequent years
- Expected accrual end date
- Objectives of protocol with supporting data and list the number of participants who have met each objective
- Measures of efficacy
- Early stopping rules with supporting data and list the number of participants who have met the early stopping rules

- Summary of toxicities
- Abstract submissions/publications
- Summary of any recent literature that may affect the safety or ethics of the study

The study principal investigator and Research Patient Coordinator will monitor for serious toxicities on an ongoing basis. Once the principal investigator or Research Patient Coordinator becomes aware of an adverse event, the AE will be reported to the HRPO and QASMC according to institutional guidelines.

13.3 Developmental therapeutics

Given the nature of this human gene transfer protocol, the principal investigator will monitor and analyze study data as they become available and will review this data on a monthly basis with the Developmental Therapeutics Group at the Siteman Cancer Center. This is an independent group that will provide more rigorous oversight than is routinely provided by the QASMC and the HRPO. Craig Lockhart M.D., Ph.D. is an Associate Professor of Medicine in the Division of Oncology and the leader of this group. As the Director of the Developmental Therapeutics Group, he devotes 20% of his time to overseeing and managing early phase clinical trials. These trials consist of all phase 1 trials and selected Phase 2 trials. He will advise Dr. Gillanders in the proper conduct of this study and will review patient treatment and all toxicities in her weekly Phase 1 meeting. In addition, Dr. Lockhart will assist in the oversight of the regulatory and data management personnel involved in this clinical trial.

13.4 NIH Recombinant DNA Advisory Committee

13.4.1 Initiation of the clinical investigation

Appendix M-I-C-1 of the NIH Guidelines for Research Involving Recombinant DNA Molecules specify:

> *No later than 20 working days after enrollment (see definition of enrollment in Section I-E-7) of the first research participant in a human gene transfer experiment, the Principal Investigator(s) shall submit the following documentation to NIH OBA: (1) a copy of the informed consent document approved by the Institutional Review Board (IRB); (2) a copy of the protocol approved by the Institutional Biosafety Committee (IBC) and IRB; (3) a copy of the final IBC approval from the clinical trial site; (4) a copy of the final IRB approval; (5) a brief written report that includes the following information: (a) how the investigator(s) responded to each of the RAC's recommendations on the protocol (if applicable); and (b) any modifications to the protocol as required by FDA; (6) applicable NIH grant number(s); (7) the FDA Investigational New Drug Application (IND) number; and (8) the date of the initiation of the trial. The purpose of requesting the FDA IND number is for facilitating interagency collaboration in the Federal oversight of human gene transfer research.*

13.4.2 Annual reports

Appendix M-I-C-3 of the NIH Guidelines for Research Involving Recombinant DNA Molecules specify:

> *Within 60 days after the one-year anniversary of the date on which the investigational new drug (IND) application went into effect, and after each subsequent anniversary until the trial is completed, the Principal Investigator (or delegate) shall submit the information set forth in (a), (b), and (c). When multiple studies are conducted under the single IND, the Principal Investigator (or delegate) may choose to submit a single annual report covering all studies, provided that each study is identified by its OBA protocol number.*
>
> *(a) Clinical Trial Information. A brief summary of the status of each trial in progress and each trial completed during the previous year. The summary is required to include the following information for each trial: (1) the title and purpose of the trial; (2) clinical site; (3) the Principal Investigator; (4) clinical protocol identifiers, including the NIH OBA protocol number, NIH grant number(s) (if applicable), and the FDA IND application number; (5) participant population (such as disease indication and general age group, e.g., adult or pediatric); (6) the total number of participants planned for inclusion in the trial; the number entered into the trial to date; the number whose participation in the trial was completed; and the number who dropped out of the trial with a brief description of the reasons; (7) the status of the trial, e.g., open to accrual of subjects, closed but data collection ongoing, or fully completed, and (8) if the trial has been completed, a brief description of any study results.*

(b) Progress Report and Data Analysis. Information obtained during the previous year's clinical and non-clinical investigations, including: (1) a narrative or tabular summary showing the most frequent and most serious adverse experiences by body system; (2) a summary of all serious adverse events submitted during the past year; (3) a summary of serious adverse events that were expected or considered to have causes not associated with the use of the gene transfer product such as disease progression or concurrent medications; (4) if any deaths have occurred, the number of participants who died during participation in the investigation and causes of death; and (5) a brief description of any information obtained that is pertinent to an understanding of the gene transfer product's actions, including, for example, information about dose-response, information from controlled trials, and information about bioavailability.

(c) A copy of the updated clinical protocol including a technical and non-technical abstract.

14 STATISTICAL CONSIDERATIONS

14.1 Study design/objectives

This is a phase 1 open-label study to evaluate the safety and immunogenicity of the personalized polyepitope DNA vaccines. The personalized polyepitope DNA vaccines will be formulated as naked plasmid DNA vaccines, and will be administered at a dose of 2 mg intramuscularly using an integrated electroporation administration system (TDS-IM system, Ichor Medical Systems) (2 doses per vaccination time point, 4 mg total per time point). The hypothesis of this study is that the personalized polyepitope DNA vaccines will be safe for human administration and capable of generating measurable CD8 T cell responses.

The primary objective is to evaluate the safety of the personalized polyepitope DNA vaccines.

The secondary objective is to evaluate the immunogenicity of the personalized polyepitope DNA vaccines. Immunogenicity will be measured by ELISPOT analysis and multiparameter flow cytometry, surrogates for CD8 T cell function.

Exploratory objectives are to evaluate the immunogenicity, and clinical response that may be associated with immune responses induced by the personalized polyepitope DNA vaccines.

14.2 Sample size and accrual

Development of therapeutic cancer vaccines is currently an area of intense research interest. The traditional paradigm for phase 1 clinical trials has been heavily influenced by phase 1 trials of chemotherapeutic agents, where dose escalation designs are appropriate given the rather narrow dose versus safety concerns of these agents. However, there are considerable differences between therapeutic cancer vaccines and chemotherapeutic agents that have important implications for early clinical development. Consistent with recommendations published in recent statistical literature, the general philosophy of this phase 1 clinical trial is to facilitate a prompt preliminary evaluation of the safety and immunogenicity of the personalized polyepitope DNA vaccines. The sample size determination is mainly based on clinical feasibility rather than statistical power [50]. However, the sample size (n=30) will provide a reasonably reliable estimate of the safety and immunogenicity of the vaccine.

14.2.1 Sample Size Calculations for Safety

The primary objective of this study is to evaluate the safety of the personalized polyepitope DNA vaccines. We have chosen to power the study to provide a reasonable ability to detect serious adverse events associated with vaccine administration.

Sample size calculations for safety are expressed in terms of the ability to detect serious adverse events. The ability of the study to identify serious adverse events is best expressed by the maximum true rate of events that would be unlikely to be observed and the minimum true rate of events that would very likely be observed. Specifically, for the sample size in the study (n=30), there is a >95% chance of observing at least 1 serious adverse event if the true rate of such an event is at least 0.10. Conversely, there is a <1% chance that we would observe 3 or more serious adverse events if the true rate is less than 0.01. Probabilities of observing 0 or 3 or more serious adverse events among a sample size of n=30 are presented in the Table below. Although we believe that the true event rate is likely to be quite low (between 0.010 and 0.100), this table presents a range of true event rates in an attempt to illustrate the sensitivity of this study to identify potential safety problems with the personalized polyepitope DNA vaccines.

Probability of detecting SAE for different safety scenarios

True Event rate	Pr(0/30)	Pr(3+/30)
0.005	0.860	<0.001
0.010	0.740	0.003
0.035	0.353	0.086
0.050	0.215	0.188
0.100	0.042	0.589
0.150	0.008	0.849
0.200	0.001	0.956
0.300	<0.001	0.998
0.400	<0.001	> 0.999

14.2.2 Sample Size Calculations for Immunogenicity

The secondary objective of this trial is to evaluate the immunogenicity of the personalized polyepitope DNA vaccines. We have chosen to power the study to provide a reasonably reliable estimate of the ability of the DNA vaccines to induce an immune response as measured by ELISPOT analysis. The Table below gives the exact 95% confidence interval for any possible number of responses out of the 30 subjects in the trial. For example, if we observe 5 responses out of 30 subjects in the trial, our 95% exact binomial confidence interval for the true rate will range from 0.056 to 0.347. This table is an attempt to illustrate the reliability of the estimate of immunogenicity given a sample size of 30 subjects.

95% Confidence Intervals for all possible observed rates of immune responses

	95% Confidence Interval	
Observed Proportion	Lower Bound	Upper Bound
0/30	0	0.116
1/30	0.001	0.172
2/30	0.008	0.221
3/30	0.021	0.265
4/30	0.038	0.307
5/30	0.056	0.347
6/30	0.077	0.386
7/30	0.099	0.423
8/30	0.123	0.459
9/30	0.147	0.494
10/30	0.173	0.528
11/30	0.199	0.561
12/30	0.227	0.594
13/30	0.255	0.623
14/30	0.283	0.657
15/30	0.313	0.687

14.3 Data Analysis

14.3.1 Primary objective: safety analysis

Toxicity evaluation is the primary objective of this trial. The data will be descriptive, and standard toxicity definitions and criteria will be used as outlined in the National Cancer Institute Common Terminology Criteria for Adverse Events version 4.0.

Since enrollment will be concurrent with the first dose of the personalized breast cancer DNA vaccines, all subjects will have received at least one vaccination, and all subjects will provide at least some safety data.

The number and percentage of subjects experiencing each type of adverse event will be tabulated by severity, and relationship to treatment. If appropriate, confidence intervals will be used to characterize the precision of the estimate. A complete listing of adverse events will also be tabulated, and will provide details including severity, relationship to treatment, onset, duration, and outcome.

Laboratory data measured on a continuous scale will be characterized by summary statistics (mean and standard deviation). Boxplots of laboratory data will be generated for baseline values and for values measured during and after protocol therapy at each specific time point. Each boxplot will show the median, 1st and 3rd quartiles. Outliers will be individually plotted in a separate graph, as appropriate.

14.3.2 Secondary objective: immune response as measured by ELISPOT analysis and multiparametric flow cytometry

Immune response as measured by ELISPOT analysis and multiparametric flow cytometry is the secondary objective of this trial. The frequency of antigen-specific CD8 T cells at each time will be summarized using means, standard deviations and medians, and the change over time will also be compared using two-ANOVA for repeated measurement data. The immunogenicity of the personalized polyepitope DNA vaccine will also be analyzed qualitatively by summarizing the phenotypic and functional characteristics of epitope-specific CD8 T cells. Responses will be considered positive if the number of T cells after vaccination is greater than two standard deviations above the mean before vaccination [72]. The frequency of positive responses at each time point will be assessed and binomial response rates with 95% confidence interval estimates will be presented. In addition to presenting the binomial response rates, graphical and tabular summaries of the underlying distributions will be made.

The safety and immune response data from this study will also be compared to data from an ongoing phase 1 clinical trial of MAM-A DNA vaccine in breast cancer patients with stable metastatic disease (Clinical trials.gov identifier NCT00807781). To determine whether the observed difference is larger than might be expected by chance, a permutation test will be used to compare the observed test statistic to the distribution of test statistics that would be seen if there were no difference between the two studies. Specifically, we will randomly shuffle the data and calculate the test statistic from the shuffled data. This procedure will be repeated 10,000 times and the resultant testing statistics will provide an accurate representation of the null distribution. The observed test statistics of between-study differences will be compared to the null distributions. For each outcome, the permuted p-value will be the fraction of permuted samples that resulted in a small statistic than the original sample (Westfall and Young, 1993).

14.3.3 Exploratory objectives

Blood samples will be obtained at multiple time points (prior to vaccination, during, and post-vaccination) in order to procure PBMC for correlative studies. Functional studies will include ELISPOT, T cell poly-functionality by intra-cellular cytokine and degranulation analysis using multi-parametric flow cytometry, and cytotoxicity assays as detailed in Section 10.

Time to disease progression will be evaluated with physical examination and diagnostic imaging as clinically indicated. The time to disease progression will be described using Kaplan-Meier product limited method. The median progression-free survival (PFS) and its 95% CI will be estimated. The association between clinical response and immunogenicity will be explored by comparing the differences in the frequency of antigen-specific CD8 T cells , as well as the phenotypic and functional characteristics, between responders versus non-responders, Permutation test will be used to determine whether the observed difference is larger than might be expected by chance, while the null distribution of test statistics will be generated using 10,000 permutation samples where the response status will be randomly re-shuffled. The observed test statistics will be compared to the null distributions. For each outcome, the permuted p-value will be the fraction of permuted samples that resulted in a small statistic than the original sample (Westfall and Young, 1993).

15 DATA MANAGEMENT

Data collected will be collected using paper case report forms or electronic data capture forms using REDCap or equivalent.

Case report forms with appropriate source documentation will be completed according to the schedule below.

**Case Report Form**	**Submission Schedule**
Original Consent Form	Prior to registration
Registration Form<br>Eligibility Form<br>Demographics Form<br>On-Study Form	Prior to starting treatment
Physical Exam Form	Baseline<br>Day 1<br>Day 15<br>Day 29<br>Day 43<br>Day 57<br>Day 71<br>Week 24
Vaccine Administration Form	Day 1<br>Day 29<br>Day 57
Immune Monitoring Form	Baseline<br>Day 1<br>Day 15<br>Day 29<br>Day 57<br>Day 71<br>Week 24<br>Week 52
Imaging Form	Baseline
Toxicity Form	Baseline<br>Day 1<br>Day 15<br>Day 29<br>Day 43<br>Day 57<br>Day 71<br>Week 24<br>Week 52
Concomitant Medications Form	Continuous
Follow Up Form	Week 52<br>Year 2<br>Year 3<br>Year 4
MedWatch Form	See Section 12.0 for reporting requirements

16 REGULATORY AND ETHICAL OBLIGATIONS

16.1 Informed consent

In accordance with US FDA regulations (21 CFR 50) and guidelines (Federal Register, May 9, 1997, Vol. 62, Number 90 - ICH Good Clinical Practice Consolidated Guideline) it is the investigator's responsibility to ensure that witnessed informed consent is obtained from the subject before participating in an investigational study, after an adequate explanation of the purpose, methods, risks, potential benefits and subject responsibilities of the study. Procedures that are to be performed as part of the practice of medicine and which would be done whether or not study entry was contemplated, such as for diagnosis or treatment of a disease or medical condition, may be performed and the results subsequently used for determining study eligibility without first obtaining consent. On the other hand, informed consent must be obtained prior to initiation of any screening procedures that are performed solely for the purpose of determining eligibility for research.

Each subject must be given a copy of the informed consent. The original signed consent must be retained in the institution's records and is subject to review by the sponsor, the HRPO and any other applicable regulatory agencies responsible for the conduct of the institution. All elements listed in the ICH Good Clinical Practice guidelines must be included in the informed consent.

Informed consent will be obtained by either the principal investigator or by individuals approved by the principal investigator and whose names have been submitted to the IRB. Informed consent will be obtained from the subject after the details of the protocol have been reviewed. The individual responsible for obtaining consent will assure, prior to signing of the informed consent, that the subject has had all questions regarding therapy and the protocol answered.

16.2 Institutional Review Board

In accordance with US FDA regulations (21 CFR 56) and guidelines (Federal Register, May 9, 1997 Vol. 62 Number 90 - ICH Good Clinical Practice Consolidated Guideline) all research involving human subjects must be reviewed and approved by the local IRB. All modifications to the protocol, consent forms, or other study documents must be reviewed and approved by the local IRB. At Washington University School of Medicine, the Human Research Protection Office serves as the local IRB.

16.3 Subject confidentiality

In order to ensure subject confidentiality, each subject will be assigned a study number. Subject samples and medical information will be de-identified and labeled with the study number. The link between subject identification and study number will be safeguarded in a secure file in a locked room, and access will be restricted to the principal investigator, study coordinator, and other co-investigators as necessary.

Collected data will be recorded on case report forms. Case report forms will be safeguarded in a locked cabinet and/or a password-protected secure computer drive and access will be restricted to the principal investigator, study coordinator, and other co-investigators as necessary. Subject medical information related to, or obtained for the purposes of this trial are confidential, and disclosure to third parties is prohibited. The exception is regulatory authorities including the FDA, NIH/OBA, and the local IRB. Data from this study must be available for inspection on request of regulatory authorities including the FDA and the local IRB.

17 ADMINISTRATIVE AND LEGAL OBLIGATIONS

17.1 Study documentation and retention of records

17.1.1 Study documentation

Source documents are original documents, data, and records from which the subject's data are obtained. These include but are not limited to hospital records, clinical and office charts, laboratory and pharmacy records, diaries, diagnostic imaging studies, and correspondence.

The principal investigator and staff are responsible for maintaining a comprehensive file of all study-related documents, suitable for inspection at any time by representatives from the PRMC, HRPO, FDA, and any other applicable regulatory agency.

Pertinent documents in the study file include:

(1) The original protocol with all amendments

(2) Curriculum vitae of principal investigator and co-investigators

(3) Approval notification and any other correspondence with the PRMC, HRPO, NIH RAC and FDA

Pertinent documents in each individual subject file include:

(1) Informed consent forms

(2) Case report forms

(3) Supporting copies of source documentation

All original source documentation must be readily available.

17.1.2 Retention of records

The principal investigator must retain records related to this study including protocols; amendments; IRB/IBC approvals; FDA IND records and other correspondence; completed, signed and dated consent forms; patient medical records; case report forms; drug accountability records and any other correspondence related to the conduct of the study.

U.S. FDA regulations (21 CFR 312.62[c]) require that all records pertaining to the conduct of this study, must be retained by the responsible investigator for a minimum of 2 years after marketing application approval. If no application is filed, these records must be kept 3 years after the investigation is discontinued and the U.S. FDA and the applicable local health authorities are notified.

17.2 Policy regarding research-related injuries

Washington University School of Medicine investigators and their staffs will try to reduce, control, and treat any complications from this research.

Any subjects who believe that they have been injured as a result of participation in this study will be instructed to contact the principal investigator, William E. Gillanders, M.D. at (314) 747-0072. Alternatively, they can contact Dr. Jonathan Green, Chairman of the Human Research Protection Office, at (800) 438-0445.

Decisions about payment for medical treatment for research-related injuries will be made by Washington University School of Medicine.

In general, Washington University School of Medicine will provide no long-term medical care or financial compensation for research-related injuries.

17.3 Study termination

The principal investigator and the Siteman Cancer Center reserve the right to terminate the study. The principal investigator will notify the PRMC and HRPO in writing of the study's completion or early termination.

18 REFERENCES

1. van der Bruggen P, Traversari C, Chomez P, Lurquin C, De Plaen E, Van den Eynde B, Knuth A, Boon T: **A gene encoding an antigen recognized by cytolytic T lymphocytes on a human melanoma**. *Science* 1991, **254**(5038):1643-1647.
2. Peshwa MV, Shi JD, Ruegg C, Laus R, van Schooten WC: **Induction of prostate tumor-specific CD8+ cytotoxic T-lymphocytes in vitro using antigen-presenting cells pulsed with prostatic acid phosphatase peptide**. *Prostate* 1998, **36**(2):129-138.
3. Peoples GE, Goedegebuure PS, Smith R, Linehan DC, Yoshino I, Eberlein TJ: **Breast and ovarian cancer-specific cytotoxic T lymphocytes recognize the same HER2/neu-derived peptide**. *Proc Natl Acad Sci U S A* 1995, **92**(2):432-436.
4. Parmiani G, De Filippo A, Novellino L, Castelli C: **Unique human tumor antigens: immunobiology and use in clinical trials**. *J Immunol* 2007, **178**(4):1975-1979.
5. Sensi M, Anichini A: **Unique tumor antigens: evidence for immune control of genome integrity and immunogenic targets for T cell-mediated patient-specific immunotherapy**. *Clin Cancer Res* 2006, **12**(17):5023-5032.
6. Wolfel T, Hauer M, Schneider J, Serrano M, Wolfel C, Klehmann-Hieb E, De Plaen E, Hankeln T, Meyer zum Buschenfelde KH, Beach D: **A p16INK4a-insensitive CDK4 mutant targeted by cytolytic T lymphocytes in a human melanoma**. *Science (New York, NY* 1995, **269**(5228):1281-1284.
7. Wang RF, Wang X, Atwood AC, Topalian SL, Rosenberg SA: **Cloning genes encoding MHC class II-restricted antigens: mutated CDC27 as a tumor antigen**. *Science (New York, NY* 1999, **284**(5418):1351-1354.
8. Echchakir H, Mami-Chouaib F, Vergnon I, Baurain JF, Karanikas V, Chouaib S, Coulie PG: **A point mutation in the alpha-actinin-4 gene generates an antigenic peptide recognized by autologous cytolytic T lymphocytes on a human lung carcinoma**. *Cancer research* 2001, **61**(10):4078-4083.
9. Brandle D, Brasseur F, Weynants P, Boon T, Van den Eynde B: **A mutated HLA-A2 molecule recognized by autologous cytotoxic T lymphocytes on a human renal cell carcinoma**. *The Journal of experimental medicine* 1996, **183**(6):2501-2508.
10. Ding L, Ellis MJ, Li S, Larson DE, Chen K, Wallis JW, Harris CC, McLellan MD, Fulton RS, Fulton LL *et al*: **Genome remodelling in a basal-like breast cancer metastasis and xenograft**. *Nature* 2010, **464**(7291):999-1005.
11. Ellis MJ, Ding L, Shen D, Luo J, Suman VJ, Wallis JW, Van Tine BA, Hoog J, Goiffon RJ, Goldstein TC *et al*: **Whole-genome analysis informs breast cancer response to aromatase inhibition**. *Nature* 2012, **486**(7403):353-360.
12. Sjoblom T, Jones S, Wood LD, Parsons DW, Lin J, Barber TD, Mandelker D, Leary RJ, Ptak J, Silliman N *et al*: **The consensus coding sequences of human breast and colorectal cancers**. *Science (New York, NY* 2006, **314**(5797):268-274.
13. Srivastava N, Srivastava PK: **Modeling the repertoire of true tumor-specific MHC I epitopes in a human tumor**. *PLoS One* 2009, **4**(7):e6094.
14. Parker KC, Bednarek MA, Coligan JE: **Scheme for ranking potential HLA-A2 binding peptides based on independent binding of individual peptide side-chains**. *J Immunol* 1994, **152**(1):163-175.
15. Rammensee H, Bachmann J, Emmerich NP, Bachor OA, Stevanovic S: **SYFPEITHI: database for MHC ligands and peptide motifs**. *Immunogenetics* 1999, **50**(3-4):213-219.
16. Gubin MM, Zhang X, Schuster H, Caron E, Ward JP, Noguchi T, Ivanova Y, Hundal J, Arthur CD, Krebber WJ *et al*: **Checkpoint blockade cancer immunotherapy targets tumour-specific mutant antigens**. *Nature* 2014, **515**(7528):577-581.
17. Matsushita H, Vesely MD, Koboldt DC, Rickert CG, Uppaluri R, Magrini VJ, Arthur CD, White JM, Chen YS, Shea LK *et al*: **Cancer exome analysis reveals a T-cell-dependent mechanism of cancer immunoediting**. *Nature* 2012, **482**(7385):400-404.
18. Cox GJ, Zamb TJ, Babiuk LA: **Bovine herpesvirus 1: immune responses in mice and cattle injected with plasmid DNA**. *J Virol* 1993, **67**(9):5664-5667.

19. Davis HL, Michel ML, Whalen RG: **DNA-based immunization induces continuous secretion of hepatitis B surface antigen and high levels of circulating antibody**. *Hum Mol Genet* 1993, **2**(11):1847-1851.
20. Fynan EF, Webster RG, Fuller DH, Haynes JR, Santoro JC, Robinson HL: **DNA vaccines: protective immunizations by parenteral, mucosal, and gene-gun inoculations**. *Proc Natl Acad Sci U S A* 1993, **90**(24):11478-11482.
21. Tang DC, DeVit M, Johnston SA: **Genetic immunization is a simple method for eliciting an immune response**. *Nature* 1992, **356**(6365):152-154.
22. Ulmer JB, Donnelly JJ, Parker SE, Rhodes GH, Felgner PL, Dwarki VJ, Gromkowski SH, Deck RR, DeWitt CM, Friedman A *et al*: **Heterologous protection against influenza by injection of DNA encoding a viral protein**. *Science* 1993, **259**(5102):1745-1749.
23. Wang B, Ugen KE, Srikantan V, Agadjanyan MG, Dang K, Refaeli Y, Sato AI, Boyer J, Williams WV, Weiner DB: **Gene inoculation generates immune responses against human immunodeficiency virus type 1**. *Proc Natl Acad Sci U S A* 1993, **90**(9):4156-4160.
24. Donnelly JJ, Ulmer JB, Shiver JW, Liu MA: **DNA vaccines**. *Annu Rev Immunol* 1997, **15**:617-648.
25. Gurunathan S, Klinman DM, Seder RA: **DNA vaccines: immunology, application, and optimization***. *Annual review of immunology* 2000, **18**:927-974.
26. Stevenson FK, Rice J, Ottensmeier CH, Thirdborough SM, Zhu D: **DNA fusion gene vaccines against cancer: from the laboratory to the clinic**. *Immunol Rev* 2004, **199**:156-180.
27. Dupuis M, Denis-Mize K, Woo C, Goldbeck C, Selby MJ, Chen M, Otten GR, Ulmer JB, Donnelly JJ, Ott G *et al*: **Distribution of DNA vaccines determines their immunogenicity after intramuscular injection in mice**. *J Immunol* 2000, **165**(5):2850-2858.
28. Aihara H, Miyazaki J: **Gene transfer into muscle by electroporation in vivo**. *Nat Biotechnol* 1998, **16**(9):867-870.
29. Mathiesen I: **Electropermeabilization of skeletal muscle enhances gene transfer in vivo**. *Gene Ther* 1999, **6**(4):508-514.
30. Mir LM, Bureau MF, Gehl J, Rangara R, Rouy D, Caillaud JM, Delaere P, Branellec D, Schwartz B, Scherman D: **High-efficiency gene transfer into skeletal muscle mediated by electric pulses**. *Proc Natl Acad Sci U S A* 1999, **96**(8):4262-4267.
31. Widera G, Austin M, Rabussay D, Goldbeck C, Barnett SW, Chen M, Leung L, Otten GR, Thudium K, Selby MJ *et al*: **Increased DNA vaccine delivery and immunogenicity by electroporation in vivo**. *J Immunol* 2000, **164**(9):4635-4640.
32. Boyer JD, Robinson TM, Kutzler MA, Parkinson R, Calarota SA, Sidhu MK, Muthumani K, Lewis M, Pavlakis G, Felber B *et al*: **SIV DNA vaccine co-administered with IL-12 expression plasmid enhances CD8 SIV cellular immune responses in cynomolgus macaques**. *J Med Primatol* 2005, **34**(5-6):262-270.
33. Chong SY, Egan MA, Kutzler MA, Megati S, Masood A, Roopchard V, Garcia-Hand D, Montefiori DC, Quiroz J, Rosati M *et al*: **Comparative ability of plasmid IL-12 and IL-15 to enhance cellular and humoral immune responses elicited by a SIVgag plasmid DNA vaccine and alter disease progression following SHIV(89.6P) challenge in rhesus macaques**. *Vaccine* 2007, **25**(26):4967-4982.
34. Hirao LA, Wu L, Khan AS, Hokey DA, Yan J, Dai A, Betts MR, Draghia-Akli R, Weiner DB: **Combined effects of IL-12 and electroporation enhances the potency of DNA vaccination in macaques**. *Vaccine* 2008, **26**(25):3112-3120.
35. Hirao LA, Wu L, Khan AS, Satishchandran A, Draghia-Akli R, Weiner DB: **Intradermal/subcutaneous immunization by electroporation improves plasmid vaccine delivery and potency in pigs and rhesus macaques**. *Vaccine* 2008, **26**(3):440-448.
36. Luckay A, Sidhu MK, Kjeken R, Megati S, Chong SY, Roopchand V, Garcia-Hand D, Abdullah R, Braun R, Montefiori DC *et al*: **Effect of plasmid DNA vaccine design and in vivo electroporation on the resulting vaccine-specific immune responses in rhesus macaques**. *J Virol* 2007, **81**(10):5257-5269.
37. Otten G, Schaefer M, Doe B, Liu H, Srivastava I, zur Megede J, O'Hagan D, Donnelly J, Widera G, Rabussay D *et al*: **Enhancement of DNA vaccine potency in rhesus macaques by electroporation**. *Vaccine* 2004, **22**(19):2489-2493.

38. Otten GR, Schaefer M, Doe B, Liu H, Megede JZ, Donnelly J, Rabussay D, Barnett S, Ulmer JB: **Potent immunogenicity of an HIV-1 gag-pol fusion DNA vaccine delivered by in vivo electroporation**. *Vaccine* 2006, **24**(21):4503-4509.
39. Schadeck EB, Sidhu M, Egan MA, Chong SY, Piacente P, Masood A, Garcia-Hand D, Cappello S, Roopchand V, Megati S *et al*: **A dose sparing effect by plasmid encoded IL-12 adjuvant on a SIVgag-plasmid DNA vaccine in rhesus macaques**. *Vaccine* 2006, **24**(21):4677-4687.
40. Rice J, Ottensmeier CH, Stevenson FK: **DNA vaccines: precision tools for activating effective immunity against cancer**. *Nat Rev Cancer* 2008, **8**(2):108-120.
41. Weiner DB: **DNA vaccines: Crossing a line in the sand Introduction to special issue**. *Vaccine* 2008.
42. Rosenberg SA, Yang JC, Restifo NP: **Cancer immunotherapy: moving beyond current vaccines**. *Nature medicine* 2004, **10**(9):909-915.
43. Finn OJ: **Cancer vaccines: between the idea and the reality**. *Nat Rev Immunol* 2003, **3**(8):630-641.
44. Liyanage UK, Moore TT, Joo HG, Tanaka Y, Herrmann V, Doherty G, Drebin JA, Strasberg SM, Eberlein TJ, Goedegebuure PS *et al*: **Prevalence of regulatory T cells is increased in peripheral blood and tumor microenvironment of patients with pancreas or breast adenocarcinoma**. *JImmunol* 2002, **169**(5):2756.
45. Wolf AM, Wolf D, Steurer M, Gastl G, Gunsilius E, Grubeck-Loebenstein B: **Increase of regulatory T cells in the peripheral blood of cancer patients**. *Clin Cancer Res* 2003, **9**(2):606-612.
46. Almand B, Clark JI, Nikitina E, van Beynen J, English NR, Knight SC, Carbone DP, Gabrilovich DI: **Increased production of immature myeloid cells in cancer patients: a mechanism of immunosuppression in cancer**. *J Immunol* 2001, **166**(1):678-689.
47. Almand B, Resser JR, Lindman B, Nadaf S, Clark JI, Kwon ED, Carbone DP, Gabrilovich DI: **Clinical significance of defective dendritic cell differentiation in cancer**. *Clin Cancer Res* 2000, **6**(5):1755-1766.
48. Hoos A, Parmiani G, Hege K, Sznol M, Loibner H, Eggermont A, Urba W, Blumenstein B, Sacks N, Keilholz U *et al*: **A clinical development paradigm for cancer vaccines and related biologics**. *J Immunother* 2007, **30**(1):1-15.
49. Schlom J, Arlen PM, Gulley JL: **Cancer vaccines: moving beyond current paradigms**. *Clin Cancer Res* 2007, **13**(13):3776-3782.
50. Simon RM, Steinberg SM, Hamilton M, Hildesheim A, Khleif S, Kwak LW, Mackall CL, Schlom J, Topalian SL, Berzofsky JA: **Clinical trial designs for the early clinical development of therapeutic cancer vaccines**. *Journal of clinical oncology : official journal of the American Society of Clinical Oncology* 2001, **19**(6):1848-1854.
51. Balko JM, Giltnane JM, Wang K, Schwarz LJ, Young CD, Cook RS, Owens P, Sanders ME, Kuba MG, Sanchez V *et al*: **Molecular Profiling of the Residual Disease of Triple-Negative Breast Cancers after Neoadjuvant Chemotherapy Identifies Actionable Therapeutic Targets**. *Cancer discovery* 2014.
52. Bowne WB, Srinivasan R, Wolchok JD, Hawkins WG, Blachere NE, Dyall R, Lewis JJ, Houghton AN: **Coupling and uncoupling of tumor immunity and autoimmunity**. *The Journal of experimental medicine* 1999, **190**(11):1717-1722.
53. Hawkins WG, Gold JS, Dyall R, Wolchok JD, Hoos A, Bowne WB, Srinivasan R, Houghton AN, Lewis JJ: **Immunization with DNA coding for gp100 results in CD4 T-cell independent antitumor immunity**. *Surgery* 2000, **128**(2):273-280.
54. Prazeres DM, Monteiro GA, Ferreira GN, Diogo MM, Ribeiro SC, Cabral JM: **Purification of plasmids for gene therapy and DNA vaccination**. *Biotechnol Annu Rev* 2001, **7**:1-30.
55. Manoj S, Babiuk LA, van Drunen Littel-van den Hurk S: **Approaches to enhance the efficacy of DNA vaccines**. *Crit Rev Clin Lab Sci* 2004, **41**(1):1-39.
56. Donnelly J, Berry K, Ulmer JB: **Technical and regulatory hurdles for DNA vaccines**. *Int J Parasitol* 2003, **33**(5-6):457-467.
57. Scheerlinck JP, Karlis J, Tjelle TE, Presidente PJ, Mathiesen I, Newton SE: **In vivo electroporation improves immune responses to DNA vaccination in sheep**. *Vaccine* 2004, **22**(13-14):1820-1825.

58. Babiuk S, Baca-Estrada ME, Foldvari M, Storms M, Rabussay D, Widera G, Babiuk LA: **Electroporation improves the efficacy of DNA vaccines in large animals**. *Vaccine* 2002, **20**(27-28):3399-3408.
59. Luxembourg A, Hannaman D, Ellefsen B, Nakamura G, Bernard R: **Enhancement of immune responses to an HBV DNA vaccine by electroporation**. *Vaccine* 2006, **24**(21):4490-4493.
60. Dunn GP, Bruce AT, Sheehan KC, Shankaran V, Uppaluri R, Bui JD, Diamond MS, Koebel CM, Arthur C, White JM *et al*: **A critical function for type I interferons in cancer immunoediting**. *Nat Immunol* 2005, **6**(7):722-729.
61. Dunn GP, Koebel CM, Schreiber RD: **Interferons, immunity and cancer immunoediting**. *Nat Rev Immunol* 2006, **6**(11):836-848.
62. Dunn GP, Old LJ, Schreiber RD: **The three Es of cancer immunoediting**. *Annu Rev Immunol* 2004, **22**:329-360.
63. Shankaran V, Ikeda H, Bruce AT, White JM, Swanson PE, Old LJ, Schreiber RD: **IFNgamma and lymphocytes prevent primary tumour development and shape tumour immunogenicity**. *Nature* 2001, **410**(6832):1107-1111.
64. Diamond MS, Kinder M, Matsushita H, Mashayekhi M, Dunn GP, Archambault JM, Lee H, Arthur CD, White JM, Kalinke U *et al*: **Type I interferon is selectively required by dendritic cells for immune rejection of tumors**. *The Journal of experimental medicine* 2011, **208**(10):1989-2003.
65. Dunn GP, Bruce AT, Ikeda H, Old LJ, Schreiber RD: **Cancer immunoediting: from immunosurveillance to tumor escape**. *Nat Immunol* 2002, **3**(11):991-998.
66. Vesely MD, Kershaw MH, Schreiber RD, Smyth MJ: **Natural innate and adaptive immunity to cancer**. *Annual review of immunology* 2011, **29**:235-271.
67. Vesely MD, Schreiber RD: **Cancer immunoediting: antigens, mechanisms, and implications to cancer immunotherapy**. *Ann N Y Acad Sci* 2013, **1284**:1-5.
68. Jaramillo A, Majumder K, Manna PP, Fleming TP, Doherty G, Dipersio JF, Mohanakumar T: **Identification of HLA-A3-restricted CD8+ T cell epitopes derived from mammaglobin-A, a tumor-associated antigen of human breast cancer**. *IntJCancer* 2002, **102**(5):499.
69. Jaramillo A, Narayanan K, Campbell LG, Benshoff ND, Lybarger L, Hansen TH, Fleming TP, Dietz JR, Mohanakumar T: **Recognition of HLA-A2-restricted mammaglobin-A-derived epitopes by CD8+ cytotoxic T lymphocytes from breast cancer patients**. *Breast Cancer ResTreat* 2004, **88**(1):29.
70. Manna PP, Jaramillo A, Majumder K, Campbell LG, Fleming TP, Dietz JR, Dipersio JF, Mohanakumar T: **Generation of CD8+ cytotoxic T lymphocytes against breast cancer cells by stimulation with mammaglobin-A-pulsed dendritic cells**. *Breast Cancer ResTreat* 2003, **79**(1):133.
71. Narayanan K, Jaramillo A, Benshoff ND, Campbell LG, Fleming TP, Dietz JR, Mohanakumar T: **Response of established human breast tumors to vaccination with mammaglobin-A cDNA**. *JNatlCancer Inst* 2004, **96**(18):1388.
72. Hobeika AC, Morse MA, Osada T, Ghanayem M, Niedzwiecki D, Barrier R, Lyerly HK, Clay TM: **Enumerating antigen-specific T-cell responses in peripheral blood: a comparison of peptide MHC Tetramer, ELISpot, and intracellular cytokine analysis**. *J Immunother* 2005, **28**(1):63-72.
73. Janetzki S, Britten CM, Kalos M, Levitsky HI, Maecker HT, Melief CJ, Old LJ, Romero P, Hoos A, Davis MM: **"MIATA"-minimal information about T cell assays**. *Immunity* 2009, **31**(4):527-528.
74. Janetzki S, Panageas KS, Ben-Porat L, Boyer J, Britten CM, Clay TM, Kalos M, Maecker HT, Romero P, Yuan J *et al*: **Results and harmonization guidelines from two large-scale international Elispot proficiency panels conducted by the Cancer Vaccine Consortium (CVC/SVI)**. *Cancer Immunol Immunother* 2008, **57**(3):303-315.

19 APPENDICES

19.1 Abbreviations

AE	Adverse Event
CBER	Center for Biologics Evaluation and Research
CFR	Code of Federal Regulations
CPA	Cooperative Project Assurance
CR	Complete response
CRA	Clinical Research Associate
CRF	Case Report Form
CT	Computed Tomography
CTCAE	Common Terminology Criteria for Adverse Events
CTEP	Cancer Therapy Evaluation Program
DNA	Deoxyribonucleic acid
DCTD	Division of Cancer Treatment and Diagnosis
ELISPOT	Enzyme-linked immunospot assay
ECOG	Eastern Cooperative Oncology Group
FDA	Food and Drug Administration
FWA	Federal-wide Assurance
GLP	Good Laboratory Practice
GMP	Good Manufacturing Practice
GPC	Gel permeation chromatography
HIPAA	Health Insurance Portability and Accountability Act
HLA	Human leukocyte antigen
HRPO	Human Research Protection Office (Institutional Review Board at WUSM)
IBC	Institutional Biosafety Committee
ICH	International Conference of Harmonization
IDB	Investigational Drug Branch
IRB	Institutional Review Board
LD	Longest diameter
MPA	Multiple Project Assurance
MRI	Magnetic Resonance Imaging
NCI	National Cancer Institute
NIH	National Institutes of Health
OBA	Office of Biotechnology Activities
OHRP	Office for Human Research Protection
PBMC	Peripheral blood mononuclear cell
PD	Progressive disease
PHI	Protected Health Information
PR	Partial response
RAC	Recombinant DNA Advisory Committee
RECIST	Response Evaluation Criteria in Solid Tumors
QASMC	Quality Assurance and Safety Monitoring Committee
SAE	Serious Adverse Event
SAS	Statistical Analysis System; Analytical software from the SAS Institute, Cary, NC
SCC	Siteman Cancer Center
SCIP	Siteman Cancer Information Portal
SD	Stable disease
SD	Standard Deviation
THF	Tetrahydrofuran
UPN	Universal Product Number
WUSM	Washington University School of Medicine
WUSTL	Washington University in Saint Louis

19.2 ECOG/Zubrod performance status scale

ECOG/Zubrod Score	Performance Status
0	Asymptomatic
1	Symptomatic, fully ambulatory
2	Symptomatic, in bed < 50% of the day
3	Symptomatic, in bed > 50% of the day but not bedridden
4	Bedridden
5	Dead

19.3 National Cancer Institute Common Terminology Criteria for Adverse Events

This study will collect adverse events using the NCI Common Terminology Criteria for Adverse Events v4.0 (CTCAE), if applicable. The CTCAE provides a descriptive terminology that is to be used for adverse event reporting. A grading (severity) scale is also provided in the CTCAE for each adverse event term. An electronic version of the CTCAE may be accessed through the web at http://ctep.cancer.gov. Alternatively, a full copy is available from the principal investigator.
