# Supplementary material for: Neoantigen DNA vaccines are safe, feasible, and induce neoantigen-specific immune responses in triple-negative breast cancer patients
Source: Genome Med. 2024 Nov 14;16:131. doi: 10.1186/s13073-024-01388-3 (PMC11562513; doi:10.1186/s13073-024-01388-3)
Supplement: Supplementary file 4 — Additional file 4: Consort check list: consort 2010 check list A (pdf): CONSORT 2010 checklist of information to include when reporting a randomised trial. [file 13073_2024_1388_MOESM4_ESM.pdf]

CONSORT 2010 checklist of information to include when reporting a randomised trial*

Section/Topic	Item No	Checklist item	Reported on page No
**Title and abstract**			
	1a	Identification as a randomised trial in the title	Not applicable. The trial is not a randomized trial. The title has been revised to indicate that the trial is a phase 1 clinical trial.
	1b	Structured summary of trial design, methods, results, and conclusions (for specific guidance see CONSORT for abstracts)	The abstract is structured and includes all of the information recommended in the CONSORT 2010 reporting guidelines.
**Introduction**			
Background and objectives	2a	Scientific background and explanation of rationale	Pages 3-4 of the manuscpript.
	2b	Specific objectives or hypotheses	Page 4 of the manuscript.
**Methods**			
Trial design	3a	Description of trial design (such as parallel, factorial) including allocation ratio	Page 5 of the manuscript.
	3b	Important changes to methods after trial commencement (such as eligibility criteria), with reasons	NA.
Participants	4a	Eligibility criteria for participants	Page 5 of the manuscript.
	4b	Settings and locations where the data were collected	Page 5 of the manuscript.
Interventions	5	The interventions for each group with sufficient details to allow replication, including how and when they were actually administered	Page 5 of the manuscript.
Outcomes	6a	Completely defined pre-specified primary and secondary outcome measures, including how and when they were assessed	Page 5 of the manuscript.
	6b	Any changes to trial outcomes after the trial commenced, with reasons	Not applicable.
Sample size	7a	How sample size was determined	The trial was a phase 1 clinical trial. The sample size was primarily based on clinical feasibility.

	7b	When applicable, explanation of any interim analyses and stopping guidelines	Not applicable.
Randomisation:			
Sequence generation	8a	Method used to generate the random allocation sequence	Not applicable.
	8b	Type of randomisation; details of any restriction (such as blocking and block size)	Not applicable.
Allocation concealment mechanism	9	Mechanism used to implement the random allocation sequence (such as sequentially numbered containers), describing any steps taken to conceal the sequence until interventions were assigned	Not applicable.
Implementation	10	Who generated the random allocation sequence, who enrolled participants, and who assigned participants to interventions	Not applicable.
Blinding	11a	If done, who was blinded after assignment to interventions (for example, participants, care providers, those assessing outcomes) and how	Not applicable.
	11b	If relevant, description of the similarity of interventions	Not applicable.
Statistical methods	12a	Statistical methods used to compare groups for primary and secondary outcomes	Not applicable. Statistical methods are described on Page 11.
	12b	Methods for additional analyses, such as subgroup analyses and adjusted analyses	Not applicable. Statistical methods are described on Page 11.
**Results**			
Participant flow (a diagram is strongly recommended)	13a	For each group, the numbers of participants who were randomly assigned, received intended treatment, and were analysed for the primary outcome	Not applicable. Please see Figure 1B for the number of patients consented, the number of patients who withdrew prior to vaccination, and the number of patients who received the vaccine.
	13b	For each group, losses and exclusions after randomisation, together with reasons	Not applicable. Please see Figure 1B for the number of patients consented, the number of patients who withdrew prior to vaccination, and the number of patients who received the vaccine.
Recruitment	14a	Dates defining the periods of recruitment and follow-up	Page 5 of the manuscript.
	14b	Why the trial ended or was stopped	The target enrollment was completed.
Baseline data	15	A table showing baseline demographic and clinical characteristics for each group	Table 1 includes baseline demographic and clinical characteristics for the cohort.

Numbers analysed	16	For each group, number of participants (denominator) included in each analysis and whether the analysis was by original assigned groups	Not applicable. Please see Figure 1B for the number of patients consented, the number of patients who withdrew prior to vaccination, and the number of patients who received the vaccine. All patients who received the vaccine were included in the safety and immunogenicity analyses.
Outcomes and estimation	17a	For each primary and secondary outcome, results for each group, and the estimated effect size and its precision (such as 95% confidence interval)	Given that this is a phase 1 clinical trial, the results are primarily descriptive as described in the protocol and the manuscript. 95% confidence interval was included for recurrence-free survival.
	17b	For binary outcomes, presentation of both absolute and relative effect sizes is recommended	Not applicable.
Ancillary analyses	18	Results of any other analyses performed, including subgroup analyses and adjusted analyses, distinguishing pre-specified from exploratory	Not applicable.
Harms	19	All important harms or unintended effects in each group (for specific guidance see CONSORT for harms)	Page 15 of the manuscript and Figure 6A describe the safety of the vaccine and all adverse events
**Discussion**			
Limitations	20	Trial limitations, addressing sources of potential bias, imprecision, and, if relevant, multiplicity of analyses	Page 17-19 of the manuscript.
Generalisability	21	Generalisability (external validity, applicability) of the trial findings	Page 18-19 of the manuscript.
Interpretation	22	Interpretation consistent with results, balancing benefits and harms, and considering other relevant evidence	Page 19-20 of the manuscript.
**Other information**			
Registration	23	Registration number and name of trial registry	ClinicalTrials.Gov registration number: NCT02348320
Protocol	24	Where the full trial protocol can be accessed, if available	Attached. Not currently publically available.
Funding	25	Sources of funding and other support (such as supply of drugs), role of funders	Page 20-21 of the manuscript.

*We strongly recommend reading this statement in conjunction with the CONSORT 2010 Explanation and Elaboration for important clarifications on all the items. If relevant, we also recommend reading CONSORT extensions for cluster randomised trials, non-inferiority and equivalence trials, non-pharmacological treatments, herbal interventions, and pragmatic trials. Additional extensions are forthcoming: for those and for up to date references relevant to this checklist, see www.consort-statement.org.
